# Isotopically Dimethyl Labeling-Based Quantitative Proteomic Analysis of Phosphoproteomes of Soybean Cultivars

**DOI:** 10.3390/biom11081218

**Published:** 2021-08-16

**Authors:** Atieh Moradi, Shuaijian Dai, Emily Oi Ying Wong, Guang Zhu, Fengchao Yu, Hon-Ming Lam, Zhiyong Wang, Al Burlingame, Chengtao Lin, Alireza Afsharifar, Weichuan Yu, Tingliang Wang, Ning Li

**Affiliations:** 1 Division of Life Science, The Hong Kong University of Science and Technology, Hong Kong, China; amoradi@connect.ust.hk (A.M.); oywongaa@connect.ust.hk (E.O.Y.W.); gzhu@ust.hk (G.Z.); 2 Institute of Biotechnology, School of Agriculture, Shiraz University, Shiraz 71946-84471, Iran; 3 Department of Chemical and Biological Engineering, The Hong Kong University of Science and Technology, Hong Kong, China; sdaiad@connect.ust.hk; 4 Department of Electronic and Computer Engineering, The Hong Kong University of Science and Technology, Hong Kong, China; fyuab@connect.ust.hk; 5 Centre for Soybean Research of the State Key Laboratory of Agrobiotechnology and School of Life Sciences, The Chinese University of Hong Kong, Hong Kong, China; honming@cuhk.edu.hk; 6 Department of Plant Biology, Carnegie Institution for Science, Stanford, CA 94305, USA; zwang@carnegiescience.edu; 7 Department of Pharmaceutical Chemistry, University of California, San Francisco, CA 94143, USA; alb@cgl.ucsf.edu; 8 Department of Molecular, Cell & Developmental Biology, University of California, Los Angeles, CA 90095, USA; clin@mcdb.ucla.edu; 9 Plant Virology Research Centre, School of Agriculture, Shiraz University, Shiraz 71946-84471, Iran; afshari@shirazu.ac.ir; 10 Tsinghua-Peking Joint Centre for Life Sciences, Centre for Structural Biology, School of Life Sciences and School of Medicine, Tsinghua University, Beijing 100084, China; 11 The HKUST Shenzhen Research Institut, Shenzhen 518057, China

**Keywords:** *Glycine max*, isotopically dimethyl labeling, soybean drought-tolerant cultivar, quantitative PTM proteomics, phosphoproteomics, overly post-translationally modified protein (OPP)

## Abstract

Isotopically dimethyl labeling was applied in a quantitative post-translational modification (PTM) proteomic study of phosphoproteomic changes in the drought responses of two contrasting soybean cultivars. A total of 9457 phosphopeptides were identified subsequently, corresponding to 4571 phosphoprotein groups and 3889 leading phosphoproteins, which contained nine kinase families consisting of 279 kinases. These phosphoproteins contained a total of 8087 phosphosites, 6106 of which were newly identified and constituted 54% of the current soybean phosphosite repository. These phosphosites were converted into the highly conserved kinase docking sites by bioinformatics analysis, which predicted six kinase families that matched with those newly found nine kinase families. The overly post-translationally modified proteins (OPP) occupies 2.1% of these leading phosphoproteins. Most of these OPPs are photoreceptors, mRNA-, histone-, and phospholipid-binding proteins, as well as protein kinase/phosphatases. The subgroup population distribution of phosphoproteins over the number of phosphosites of phosphoproteins follows the exponential decay law, Y = 4.13e^−0.098X^ − 0.04. Out of 218 significantly regulated unique phosphopeptide groups, 188 phosphoproteins were regulated by the drought-tolerant cultivar under the water loss condition. These significantly regulated phosphoproteins (SRP) are mainly enriched in the biological functions of water transport and deprivation, methionine metabolic processes, photosynthesis/light reaction, and response to cadmium ion, osmotic stress, and ABA response. Seventeen and 15 SRPs are protein kinases/phosphatases and transcription factors, respectively. Bioinformatics analysis again revealed that three members of the calcium dependent protein kinase family (CAMK family), GmSRK2I, GmCIPK25, and GmAKINβ1 kinases, constitute a phosphor-relay-mediated signal transduction network, regulating ion channel activities and many nuclear events in this drought-tolerant cultivar, which presumably contributes to the development of the soybean drought tolerance under water deprivation process.

## 1. Introduction

Soybean (*Glycine Max* (L.) Merr), similar to maize, wheat, rice, and cotton crops, is an important agricultural crop traded in global commodity markets [1]. Soybean seeds are widely consumed as human food, animal feed, and industrial material supplies [2]. The productivity of this cash crop has a profound and direct impact not only on the market price of this legume crop, and consumption behaviors of people, but also on the trading activities between countries. The high contents of premier protein (40%), nutraceutical compounds, and dietary vegetable oil (20%) of soybean seed and, especially, soybean plant’s capability in nitrogen fixation from the atmosphere, make it a unique economic crop among globally cultivated agricultural crops [3,4,5,6,7,8].

However, the production of soybean has been curtailed by numerous harsh environmental stresses, especially drought, a water deficit state of soil [9]. The increasing dryness in the semiarid lands is considered a predominant environmental stress [10,11], which has been estimated to cause 20–49% of the soybean production variance in yield [9]. Thus, how the drought stress influences the plant cell division and expansion, as well as the plant growth, development, and reproduction has become an interesting yet important research topic for plant biologists [12]. Besides the secondary messengers, such as calcium, reactive oxygen species (ROS), and inositol phosphates, which are well-known secondary messengers in mediating the drought stress-responsive gene expression [13,14], the plant hormone abscisic acid (ABA), in particular, is well-known to play an essential role in plant drought response [15]. Application of molecular biology and genetics studies in the model plant *Arabidopsis* have contributed much of our understanding of the molecular basis of plant drought response and have revealed a network of signaling components and transcription factors regulating drought tolerance [15,16]. Microarray-based gene expression profiling, cDNA sequencing, and forward genetics have unraveled hundreds of regulatory genes, such as SnRK2s, MAPKs, DREBs, AREBs, and mRNA decapping factors, that participate in development of plant drought tolerance when exposed to drought treatment [17,18,19,20]. Development of genetic engineering technologies in making transgenic plants of modified drought-tolerant genes has led to the successful open field tests for the genetically modified (GM) soybean cultivars with desirable agronomic performance traits [21,22].

With the advancement of genomics technology, the genomes of hundreds of wild type and domesticated soybean plants have been sequenced [23,24,25,26]. The available DNA sequence databases of soybean genomes provide an extensive molecular biological information for application of proteomics into the study of the soybean drought response. To that end, proteomics work has been performed in the past decade [27]. As a result, a soybean proteome database (SPD) was established, and a total of 44,704 proteins, accounting for about 80% of deduced soybean proteins from genomic sequences, were found [27,28]. Application of both label-free and stable isotope labeling-based quantitative proteomics and subcellular proteomics on drought-treated soybean plants have revealed that the osmotic adjustment, redox signaling, calcium signaling, biotinylation-regulated energy management, and the programmed cell death are all involved in the drought response [28,29], where S-adenosylmethionine (SAM) synthetase plays an important role in the redox signaling and polyamine oxidation [30] under water stress, and that the protein abundances of peroxidase and aldehyde dehydrogenase increase during drought treatment [31]. These findings are consistent with the general drought-responsive mechanisms found from proteomics studies of model plants, important crops and woody plants exposed to drought, in which major changes occur in cell sensing, signaling, reactive oxygen species scavenging, osmotic regulation, gene expression, protein synthesis/turnover, cell structure modulation, and carbohydrate/energy metabolism [32]. Integration of both drought-tolerant and -sensitive cultivars into the quantitative and differential proteomics study of various crop drought response has recently provided a new perspective to understand the molecular mechanisms underlying drought tolerance [33,34,35]. For example, a proteomics study performed on two soybean cultivars found that the tolerant cultivar expresses an RNA polymerase transcription II protein-like protein and has elevated antioxidant enzymes [36].

Phosphoproteomics, as one of the powerful PTM proteomics, have been applied into investigation of phosphorylation-mediated stress signaling as protein phosphorylation plays a key role in protein–protein interaction, enzyme activity, and protein stability [37,38,39]. The large scale of phosphoproteomic profiling of soybean phosphoproteins and phosphosites have expanded our knowledge on the overall landscape of phosphorylation sites in soybean [40,41,42,43]. Specific application of iTRAQ-based quantitative phosphoproteomics in the study of soybean and rhizobium interaction has revealed a total of 1659 non-redundant phosphorylation sites [44]. In another investigation of soybean’s stress response, a quantitative phosphoproteomics was used to find the key signaling components in soybean roots under stress treatment. In total, 11,259 phosphosites on 3747 leading phosphoproteins have been identified thus far, as shown in the Eukaryotic Phosphorylation Sites Database (EPSD, http://epsd.biocuckoo.cn/, accessed on 15 August 2020), which leads to the discovery of phosphorylation of several transcription factors, such as GmMYB173, connecting the upstream stress signals to the transcriptional control of the stress-responsive genes [45].

In this study, we chose the isotopically dimethyl labeling-based quantitative PTM proteomics approach to identify the drought-tolerant cultivar significantly regulated phosphoprotein (SRP) groups. The isotopic dimethyl labeling of peptides is one of the cost-effective, highly efficient, and reliable in vitro chemical-labeling methods applied in quantitative PTM proteomics, proteomics, and interactomics [46,47,48,49,50]. This chemical labeling approach utilizes formaldehyde and sodium cyanoborohydride to react with the primary amines of the N-terminal residues of peptides as well as with lysine side chains to generate dimethylamines [48]. The light and heavy isotope-coded dimethyl chemicals are often mixed with two groups of proteolytically digested peptides derived from the total cellular proteomes of two plant samples [49]. This in vitro chemical labeling technique also requires relatively fewer technical replicates during the identification of the significantly regulated peptides as compared to that of the label-free approach because the plant peptides are labeled and mixed before peptide enrichment that often generates large variations in amounts of targeted peptides, compromising peptide quantitation and statistical evaluation.

To understand how protein phosphorylation mediates the soybean drought response, in this study, we selected a pair of drought-tolerant and -sensitive soybean cultivars [51]. As compared to the control drought-sensitive cultivars, where the rate of leaf area expansion of the sensitive cultivars declined dramatically due to the water constraint, the tolerant cultivar, however, is able to maintain a greater leaf area expansion rate [51]. Since Zhang and Davies [52] have postulated before that the leaf expansion and stomatal behavior respond directly to soil drying before the occurrence of any detectable shoot water deficit, we therefore decided to choose the aerial part of soybean tissues for quantitative phosphoproteomic analysis.

## 2. Materials and Methods

### 2.1. Chemicals

All chemicals were ordered from Sigma Aldrich (Sigma Aldrich, St Louis, MO, USA) if not otherwise specified.

### 2.2. Plant Growth and Drought Treatment

The soybean seeds of both drought-sensitive (*Glycine max* L. cv. Union) and drought-tolerant soybean cultivars (*Glycine max* L. cv. Longhuang #3) used in this experiment were placed on the Milli-Q water-wetted tissue papers for five days to germinate. The soybean seedlings were consequently planted in soil within 40 × 20 × 8 cm plastic trays (35 plants per tray). Soil bought from Jiffy Products International BV (Zwijndrecht, The Netherlands) and that from Plantmate (Hong Kong, China) were mixed together at a ratio of 2:1 to fill the plant growth trays. Each tray contained 4 kg of soil mixture. The chamber temperature was set at 23.5 ± 1 °C and humidity was 45–65%. The light intensity of 140–180 μE/m^2^ s^1^ was applied constantly onto soybean plants. Plants grown in trays were irrigated daily (about 600 mL of water per tray) for 21 days. By 21 days old, the irrigation was stopped for 10 days on the two greenhouse-grown cultivars (Figures S1 and S2I; [51,53]). The water loss rate of the soybean leaves was measured on days 0, 2, 4, 6, 8, and 10 according to the following formulas: (fresh weight − dry weight)/fresh weight × 100 and (fresh weight-dry weight)/(fresh weight (0th day) − dry weight (0th day)) × 100) during the drought treatment [51,54]. The aerial parts of plants above cotyledons were harvested by 31 days, frozen in liquid nitrogen immediately, and stored at −80 °C for later protein extraction.

### 2.3. Protein Extraction under Denaturing Condition

The frozen soybean seedlings were ground into fine powders in liquid nitrogen pre-cold mortar and pestle. A total of 45 g of frozen tissue powders prepared from each soybean cultivar were extracted for the total cellular protein. The frozen tissue powers were mixed well with urea-based protein extraction buffer (UEB; [55]) at a ratio of 1:4 (*w/v*), which consisted of 150 mM Tris (pH 7.6), 20 mM ethylenediaminetetraacetic acid (EDTA), 0.8% sodium dodecyl sulfate (SDS), 8 M Urea, 1.2% Triton X-100, 20 mM ethylene glycol-bis(β-aminoethyl ether)-N,N,N′,N′-tetraacetic acid (EGTA), 50 mM NaF, 1% glycerol 2-phosphotase disodium hydrate, 5 mM dithiothreitol (DTT), 1 mM phenylmethylsulfonyl fluoride (PMSF), 0.5% phosphatase cocktail 2, complete EDTA free protease inhibitors cocktail, 5 mM ascorbic acid, and 2% polyvinylpolypyrrolidone (PVPP). The initial protein extract was centrifugated at 141,000× *g* for 2 h at 10 °C, and the supernatant containing the total cellular protein was precipitated using a cold solution of acetone to methanol at 12:1 (*v/v*) and stored at −20 °C for overnight. The protein precipitates were centrifugated at 18,542× *g* for 20 min, at 16 °C. The protein pellets were air-dried and re-suspended in a resuspension buffer containing 8 M urea, 50 mM Tris-HCl (pH 8.0), 0.2% sodium dodecanoate and 5 mM DTT. To remove oil present in the soybean protein pellets, the acetone precipitation was repeated for 3 times. DC protein assay (Bio-Rad DC Protein Assay Kit, Budapest, Hungary) was used to quantify the protein concentration according to a standard curve made from bovine serum albumin (BSA). A total of 6 protein samples were prepared from two soybean cultivars in three biological replicates.

### 2.4. In-Solution Trypsin Digestion and Dimethyl Labeling

The total cellular proteins were first dissolved in 5 mM DTT and incubated at room temperature for an hour to reduce protein disulfide bonds. Iodoacetamide powder was consequently added to the protein solution to make a final concentration of 50 mM, which was incubated in darkness for an hour to allow cysteine to be alkylated. Finally, DTT was added to the solution to make it a final concentration of 5 mM, which was incubated in darkness at room temperature for 30 min to quench the unreacted iodoacetamide. The protein solution was subsequently mixed with 9× volumes of pre-warmed (37 °C) trypsin digestion buffer (25 mM ammonium bicarbonate) and incubated at 37 °C overnight. The ratio of trypsin to protein was 1:20 (*w/w*). The second digestion performed by adding one part of fresh trypsin to 40 parts of protein/peptide substrates was incubated for additional 6 h. The peptides were acidified consequently with 0.2–0.4% formic acid, the precipitates of which was centrifugated at 141,000× *g* for 20 min at 14 °C. The peptide solution was further filtered through 0.22 mM cellulose acetate membrane filter (Sartorius, Goettingen, Germany). The digested peptides were desalted and enriched by C18 Sep-Pak cartridge (Waters, Manchester, UK). The peptide concentration was determined using DC assay kit (Bio-Rad DC Protein Assay Kit, Budapest, Hungary).

Before isotopic dimethyl labeling, the two peptide samples prepared from both drought-sensitive and drought-tolerant cultivar were dissolved separately into 100 mM sodium acetate (pH 5.5) with a final concentration of 1 mg/mL. Each one of the peptide samples were further divided into two equal fractions, one of which was labeled with the light isotope-coded formaldehyde chemical, ^12^CH_2_O, while the other fraction with the heavy isotope-coded formaldehyde ^13^CD_2_O. The ^12^CH_2_O-labeled peptide sample from the drought-sensitive soybean seedlings was mixed with the ^13^CD_2_O-labeled peptide sample from the drought-tolerant plants to produce forward (F) mixing peptides. On the other hand, the light isotope-labeled drought-tolerant peptides mixed with the heavy isotope-labeled peptides from the sensitive plants to produce a reciprocal (R) mixing sample. Both F and R samples were considered to be two experimental replicates of a single biological replicate. All six experimental peptide mixings (F1, F2, F3, R1, R2, and R3) derived from three biological replicates were desalted using C18 Sep-Pak cartridge (Waters, Manchester, UK). The concentration of peptides was determined using the DC assay kit (Bio-Rad DC Protein Assay Kit, Budapest, Hungary).

### 2.5. TiO_2_/Fe^3+^IMAC Phosphopeptide Enrichment

The TiO_2_ enrichment was achieved using TiO_2_ beads (Titansphere, GL Sciences, Tokyo, Japan). Before loading the peptides onto TiO_2_ resins, the resins were equilibrated once with five resin volumes of TiO_2_-washing buffer consisting of 80% acetonitrile (ACN) and 0.5% trifluoroacetic acid (TFA), followed by five resin volumes of TiO_2_-binding, and equilibrating solution (300 mg/mL lactic acid in washing buffer). The differentially dimethyl-labeled and mixed peptides of 400 mg were resuspended in TiO_2_-binding and equilibrating buffer and mixed with pre-equilibrated TiO_2_ resins at a ratio of 1 mg of peptide to 4 mg of TiO_2_. The flow-through peptides in the equilibrating buffer were removed by centrifugation in Sorvall™ ST 8 Small Benchtop centrifuge (Thermo Scientific Inc., Waltham, MA, USA) for 1 min after being incubated for an hour at room temperature with several rotations. The resins were rinsed with 5 volumes of TiO_2_-washing buffer once and with 5 volumes of TiO_2_-binding and equilibrating buffer subsequently. The resin-bound phosphopeptides were eluted off using 1.5 resin volumes of 5% ammonium aqueous solution twice, followed by once incubation of the sample in 1.5 resin volumes of 5% pyrrolidine for 5 min at room temperature. The eluted phosphopeptides were acidified with the same volume of 20% TFA immediately. The flow-through peptides sample in equilibrating buffer (300 mg/mL lactic acid in washing buffer) was desalted with Oasis HLB Cartridge (Waters, Manchester, UK) and loaded onto TiO_2_ resins again to repeat the second round of binding and elution. The two batches of eluted phosphopeptides were consequently combined and desalted using Oasis HLB Cartridge (Waters, Manchester, UK). They were then subjected to the final round of TiO_2_ enrichment.

At the immobilized metal affinity chromatography (IMAC) enrichment step, Fe^3+^NTA resins were prepared by mixing NTA agarose resins (QIAGEN, Hulsterweg, The Netherlands) with 0.1 M FeCl_3_ solution for two hours. The resulting Fe^3+^-IMAC beads were re-suspended in IMAC binding buffer (30% ACN and 250 mM acetic acid). The peptides combined from two batches of flow-through samples were mixed with Fe^3+^IMAC beads of a ratio of 1 mg of peptide to 10 µL of beads slurry and incubated for 1 h at room temperature with gentle rotations. The supernatant was removed using centrifugation, and the phosphopeptides bound on IMAC beads were washed with IMAC binding buffer twice and water once. The IMAC resins-bound phosphopeptides were eluted off using 500 mL of the elution buffer (5% ammonium hydroxide) on a shaker for 5 min at room temperature. The eluate was then acidified with 100 mL of formic acid immediately. The elution steps were repeated for two more times and each acidified with formic acid immediately. The phosphopeptides enriched from both TiO_2_ and Fe^3+^-IMAC resins were combined and desalted on Oasis HLB Cartridge (Waters, Manchester, UK). The concentration of phosphopeptides were determined thereafter and air-dried on Speed Vac (Thermo Scientific Inc., Waltham, MA, USA).

### 2.6. Fractionation of Phosphopeptides on Both WAX and SCX Columns

The double resins-enriched phosphopeptides were fractionated into a total of 74 fractions from 6 experimental replicates using both BioPureSPN™ MIDI strong cation exchange (SCX) column (100–500 mg binding capacity) and BioPureSPN™ MINI weak anion exchange (WAX) column (40–200 mg binding capacity, The Nest Group, Southborough, MA, USA). The SCX column was first equilibrated with 200 mL of 100% methanol or 100% ACN solution and centrifuged for 1 min at 110× *g* and conditioned for an hour with 400 mL of buffer consisting of 0.2 M monosodium phosphate and 0.3 M sodium acetate, which was followed by three rounds of washing with 200 mL of loading buffer (10 mM ammonium formate, 20% ACN, and 0.1% FA), and immediately centrifuged for 1.5 min at 110× *g*. The combined phosphopeptides were resuspended in 50 mL of loading buffer and loaded onto the prepared SCX column for 5 times. After washing the peptide-bound column with 200 mL of loading buffer for three times, the phosphopeptides were eluted off using 200 mL of elution buffer (500 mM ammonium formate, 20% ACN and 3% FA) of a gradient of 10–100%. The flow-through peptide solution from SCX column was desalted using HLB cartridges (Waters), dried on Speed Vac (Thermo Scientific Inc., Waltham, MA, USA) and dissolved in WAX loading buffer (5 mM Tris, pH 8.0, and 20% ACN). The WAX column was pre-conditioned with 200 mL of 100% methanol or 100% ACN solution, followed by 400 mL of buffer containing 0.2 M monosodium phosphate and 0.3 M sodium acetate for one hour. The pre-conditioned column was equilibrated using the loading buffer (5 mM Tris, pH 8.0, and 20% ACN). The phosphopeptides were finally eluted off using an elution buffer (400 mM NaCl, 30% ACN and 0.05% FA). All of the fractions eluted off from both SCX and WAX columns were air-dried on Speed Vac (Thermo Scientific Inc., Waltham, MA, USA) and desalted using ZipTip (Millipore), and desiccated on Speed Vac (Thermo Scientific Inc., Waltham, MA, USA).

### 2.7. Liquid Chromatography–Tandem Mass Spectrometry (LC–MS/MS) Analysis

The phosphopeptides were resuspended with solvent A (0.1% formic acid) and analyzed on an Easy nLC system (Thermo Fisher Scientific, Waltham, MA, USA) coupled to an Orbitrap Fusion™ Lumos™ Tribrid™ Mass Spectrometer (Thermo Fisher Scientific, Waltham, MA, USA). For each LC-MS/MS run, phosphopeptide sample was loaded onto an analytic column (Thermo Scientific EasySpray C18 column, 3 um, 15 cm × 75 um) with a flow of 0.6 or 0.3 μL/min. The phosphopeptides were separated on a 95-min gradient of 2 to 50% solvent B (ACN mix with 0.1% formic acid): 0 to 2% solvent B, 0–10 min, 0.6 μL/min; 2–5% solvent B, 10–13 min, 0.6 mL/min; 5–30% solvent B, 13–85 min, 0.6 mL/min; 30–50% solvent B, 85–87 min, 0.6 mL/min; 50–5% solvent B, 87–89 min, 0.6 mL/min; 5–5% solvent B, 89–94 min, 0.6 mL/min; 5–2% solvent B, 94–95 min, 0.6 mL/min. MS1 mass resolution was set at 120 K with *m*/*z* of 375–1500 for a maximum injection time of 50 ms while MS2 resolution at 30 K under HCD collision mode with a normalized collision energy at 30% for a maximum injection time of 100 ms. Filter type has an intensity threshold of 2.0 × 10^−4^, while the isolation mode was set as quadrupole and isolation window (*m*/*z*) as 1.6.

### 2.8. Quantitative Phosphoproteomic Analysis

The raw data files from LC–MS/MS analysis were firstly converted into the mzXML and MGF format using MS convert GUI (version: 3.0.18353 64-bit; Figure S2; [56]). The MGF files were searched against the soybean NCBI database (https://www.ncbi.nlm.nih.gov/, accessed on 29 July 2019) using a Mascot Daemon (Version 2.6.0 64-bit, Matrix Science; Figure S2; [56]) with a target-decoy strategy as previously described [57,58]. The parameters were set as follows: protease was trypsin, the maximum missed cleavage two, the peptide tolerance ±10 ppm, the MS/MS tolerance ±0.02 Da, the variable modifications were oxidation on Methionine, light dimethyl labeling was on lysine residue and N terminus of a peptide, heavy dimethyl labeling on lysine residue and N-terminus of a peptide, phosphorylation on serine, threonine and tyrosine residues of a peptide, and finally the fixed modification carbamidomethyl on cysteine. The output of the Mascot was consequently subjected to SQUA-D software (Stable isotope-based Quantitation-Dimethyl labeling, Version 1.0; [46,47]) for further XIC-based quantification. The MascotPercolator (Version 3; Figure S3) was used to estimate the false discovery rate (FDR) of PSMs, which was subsequently converted into q-value. All PSMs of a *q*-value ≤ 0.01 would be considered for a further selection for quantification. Moreover, the leading protein of the protein group is selected by default manner of the percolator. The selection criteria for quantifiable phosphopeptides to be processed by SQUA-D software was the followings: the PSM number of heavy dimethyl-labeled PTM peptides was ≥1, the PSM number of light dimethyl labeled PTM peptides PSMs ≥1, the number of different experimental replicates ≥4, the number of PSMs identified from either forward or reciprocal experiment divided by the total number of PSMs ≥0.2, the Mascot delta score ≥10 and the MS1 tolerance <0.05 Da. To eliminate the variances resulted from in tissue harvesting, protein extraction, peptide digestion, chemical labeling and sample mixing, the batch effect adjustment was also applied to the quantification of the 6 batches of phosphopeptides to finally modify the log2 ratios of UPAs (F1, F2, F3, R1, R2, and R3; Supplementary Table S5b; [46,47,59])

Eventually, those drought-tolerant genotype significantly regulated phosphopeptide groups (or called unique PTM peptide arrays, UPAs) were selected using the following criteria: (1) |log2 ratio| ≥0.75 (0.5 × SD) or ≤ −0.75 (−0.5 × SD), (2) biological replicate = 3, (3) experimental replicates ≥4, (4) the number of log2 ratios of heavy and light dimethyl-labeled phosphopeptides ≥5, (5) the number of high-quality extracted ion chromatography (XIC) from forward and reciprocal replicate ≥1, respectively (Figure S3; Supplementary Table S6a). The workflow of computational analysis of MS/MS data and XIC-based quantification is summarized in Supplemental Figure S3.

### 2.9. Kinase and Substrates Analysis

The protein kinases were fetched out and classified into 9 families from the leading proteins using iTAK (Plant Transcription factor and Protein Kinase Identifier and Classifier; [60]). The 9 kinase families consist of AGC: the cAMP-dependent protein kinase, the cGMP-dependent protein kinase and the protein kinase C family; CK1: casein kinase I family; CAMK: Ca^2+^/calmodulin-dependent protein kinase family; TKL represents tyrosine kinase-like kinase family; plant specific: plant-specific kinase family; CMGC: cyclin-dependent kinase (CDK), mitogen-activated protein kinase (MAPK), glycogen synthase kinase (GSK) and CDC-like kinase family; STE: Steril kinase family; RLK: receptor-like kinase family; others: a group of diverse kinase families except the above families. The dendrogram of the kinase classification was generated using MEGAX (Molecular Evolutionary Genetics Analysis across Computing Platforms; [61]) with the maximum likelihood estimation. The dendrogram was then exported as nwk format and reorganized by ggtree package of R.

The PSM counts of each kinase was calculated by the sum of PSMs, which had the leading protein of this kinase. The phosphosites were converted into 13-mer oligopeptide sequences with the phosphorylated amino acid located in the center. The possible kinase family for the specific substrates were predicted using GPS (Group-based Prediction System; [62]). Then, the results of prediction GPS and kinase classification were matched based on the kinase families and filtered by protein–protein interactions recorded at STRING (Search Tool for the Retrieval of Interacting Genes/Proteins; [63]) or BioGRID (Biological General Repository for Interaction Datasets, https://thebiogrid.org/, accessed on 8 September 2020) databases. The motifs for each kinase family were analyzed by Motif-ALL (version 1.0) and Motif -X (version 1.2) software [64,65] against the soybean background.

### 2.10. InterPro Protein Domain and Gene Ontology Analysis

With the limitation of annotated soybean proteins, the protein IDs in the experimental datasets were firstly converted into *Arabidopsis* homologs using NCBI Blast (https://blast.ncbi.nlm.nih.gov/Blast.cgi, accessed on 4 May 2020). The *Arabidopsis* proteins with the highest score were chosen as the homologs to those of soybean proteins. InterPro protein domain enrichment was generated online using a Database for Annotation, Visualization, and Integrated Discovery (DAVID v.6.8, https://david.ncifcrf.gov/ accessed on 5 May 2020) with the following thresholds: Gene counts ≥2 and *p*-value ≤0.05. Functional analysis including Gene Ontology Analysis (molecular functions enrichment, cellular component enrichment and biological processes enrichment) were performed using the GENEONTOLOGY (http://geneontology.org/, accessed on 5 May 2020) and the figure was drawn using R with ggplot2 packages.

### 2.11. Protein Structure Prediction and Visualization

Protein 3D structures were predicted using I-Tasser (Iterative Threading ASSEmbly Refinement, https://zhanglab.ccmb.med.umich.edu/I-TASSER/, accessed on 8 May 2020; [66]) with the default setting. ICM-Browser 3.8 and PyMOL were used for the visualization of protein 3D structure and phosphosites.

### 2.12. RT-qPCR Quantification of Gene Expression Level

The total cellular RNA was isolated using a PureLink RNA mini kit (Thermo Fisher Scientific, Waltham, MA, USA). The RNA sample was further treated with DNase to remove DNA contamination and reversely transcribed into cDNA using a Maxima First Strand cDNA Synthesis Kit (Thermo Fisher Scientific, Waltham, MA, USA). The qPCR was performed on a LightCycler^®^ 480 instrument II (384-well plate, Roche, Basel, Switzerland) using LightCycler 480 SYBR Green I Master (Roche, Switzerland) in a 384 Multiwell plate with 20 μL-reaction solution in each well (2 μL of 10× cDNA reaction mixture, 10 μL of 2× SYBR Master Mix, 0.7 μL each of 10 μM gene-specific primers and 6.6 μL of PCR-grade water). The qPCR program conditions included a pre-incubation step for 10 min at 95 °C, followed by 45 cycles of denaturation at 95 °C for 10 s, annealing at 60 °C for 15 s, and a final extension at 72 °C for 15 s. A melting curve procedure was then performed at 95 °C for 5 s with a ramp rate of 4.8 °C/s, 65 °C for 1min with a ramp rate of 2.5 °C/s and 97 °C, using a continuous acquisition mode with 5 acquisitions/°C. A final procedure of cooling was performed at 40 °C for 10 s with a ramp rate of 2 °C/s. The online program of the NCBI primer design (https://www.ncbi.nlm.nih.gov/tools/primer-blast/, accessed on 17 April 2020) was used for the gene-specific primer designing. The primers used for the validation is listed in the Supplementary Table S6a. The gene ACT6 and Fbox were used as an internal control according to previous studies [67,68]. The relative expression level of each gene was calculated by the 2-△△Ct method [69].

### 2.13. Volvox Plot Conduction

The drought-tolerant genotype significantly regulated phosphoproteins were submitted to STRING (https://string-db.org/; accessed on 10 May 2020 [63]) to perform protein interaction analysis with a moderate confidence (0.40) as well as all of the available interaction sources to build a putative protein–protein interaction network. The network was then exported as a tsv file and visualized by Cytoscape (Version 3.80; [70]). The drought regulatory and response genes were separated based on the *Arabidopsis* homolog gene annotation on *TAIR* (https://www.arabidopsis.org/index.jsp, accessed on 4 May 2020). The functional classification of genes was conducted based on the Gene Ontology analysis using the GENEONTOLOGY (http://geneontology.org/, accessed on 5 May 2020).

### 2.14. Signal Pathway Conduction

The kinase and substrates map were eventually overlapped with those significantly regulated phosphopeptide groups (as indicated in Figure S7) to generate the signal pathway model and the molecular function of each kinase or substrates were annotated by Gene Ontology. The subcellular localization of the kinase or substrates were predicated by SUBA4 (Subcellular Localization Database for *Arabidopsis* Proteins 4 [71]), using the *Arabidopsis* ortholog.

### 2.15. Statistical Analysis

The statistical significance of the results of morphological analysis and RT-qPCR was assessed using two-tailed student’s t-test, with significance represented by *, **, and *** at *p* < 0.05, *p* < 0.01, and *p* < 0.001, respectively. Quantitative data were represented as mean ± SE. Statistical methods used by SQUA-D to quantify the phosphopeptide groups include *t*-test, BH-FDR, and the batch effect adjustment [46,47].

## 3. Results

### 3.1. Preparation of Isotopic Phosphopeptides and LC–MS/MS Analysis

To investigate the phosphoproteomic changes associated with the drought-tolerant cultivar, both the drought-tolerant and -sensitive cultivars were subjected to a mild drought treatment (60% humidity in soil; Figure S1A; [51,53]), during which, both the soil humidity level and plant tissue water content were measured every two days. It was found that the drought-tolerant soybean cultivar had a significantly higher level (*p* ≤ 0.05) of water content as comparing to that of the drought-sensitive plants grown on days 8, 10, and 12 under drought treatment (Figures S1B,C and S2I). The total cellular proteins were extracted separately from both soybean cultivars at day 10 drought-treatment using a urea-based, PVPP-containing and protein-denaturing extraction buffer (Figure S2II; [55]). From each biological replicate, 45 g of aerial tissues were harvested separately from each of the two cultivars used for protein extraction (Figure S2I,II). The total cellular protein of 197.8–310 mg was isolated from each batch of plant tissues and further digested in-solution into the total cellular peptides (Figure S2II; Supplementary Table S1). A portion of the trypsin-digested peptides (150–180 mg) from the drought-tolerant soybean plant was chemically labeled using the heavy (H) isotope-coded formaldehyde ^13^CD_2_O, while the digested peptides from the drought-sensitive soybean was labeled with the light (L) isotope-coded formaldehyde ^12^CH_2_O instead (Figure S2III; Supplementary Table S1). Mixing of equal amount of two dimethyl labeled peptide samples generated a forward (F1) mixing sample. Conversely, the heavy isotope-coded formaldehyde ^13^CD_2_O was applied to label the drought-sensitive cultivar peptide sample while the light isotope-coded formaldehyde ^12^CH_2_O was used to label the drought-tolerant soybean peptide sample. The two peptide samples were mixed together to generate a reciprocal (R1) mixing sample, which was defined to be the second experimental replicate. As a result, all three biological replicates performed herein generated a total of 6 experimental replicates (Figure S2III). These L/H isotope-coded total cellular peptide samples were further enriched tandemly for phosphopeptides using TiO_2_ and Fe^3+^-IMAC affinity beads (Figure S2IV; See Material and Methods for details). The yield of phosphopeptide enrichment varied from 0.05 to 0.15% among three biological replicates (Supplementary Table S1). The phosphopeptide mixtures prepared from 6 experimental replicates were defined as F1, R1, F2, R2, F3, and R3, and further fractionated on both strong cation exchange (SCX) column and weak anion exchange (WAX) column in a consecutive order (Figure S2V; see Material and Methods for details). The amount of phosphopeptides harvested from each fraction for LC–MS/MS analysis ranged from 0.8 to 32 µg (Figure S2VI). The specification and conditions for the consequent LC–MS/MS analysis of phosphopeptides are described in Materials and Methods.

### 3.2. Profiling and Bioinformatics Analysis Phosphopeptides, Phosphosites, and Phosphoproteins

**Figure 1:**
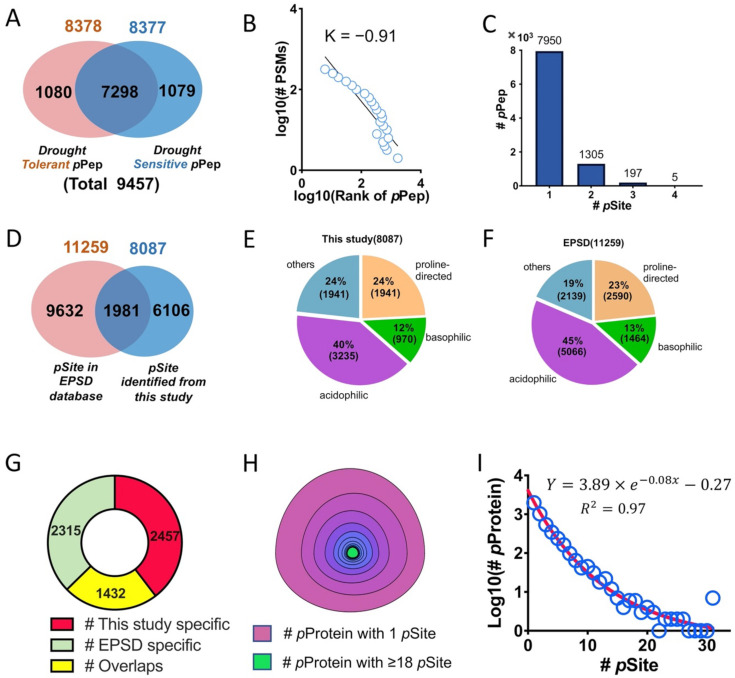
Phosphoproteomic analysis of the drought-treated soybean plants. (**A**) A Venn diagram shows the numbers of non-redundant, repeatable, and label-independent phosphopeptides (9457) of both drought-tolerant (8378) and -sensitive cultivars (8377, Supplementary Table S2d, Figure S4A). (**B**) Distribution of the PSM numbers of each soybean phosphopeptide over the rank (or index) of entire population of phosphopeptides (9457). The k (−0.91) represents the slope of fitting curve of the rank-frequency distribution by Zipf’s law (Supplementary Table S2d). (**C**) Distribution of subgroups of phosphopeptides over the specific number of phosphosite on a phosphopeptide. The pSite stands for phosphosite(s) (Supplementary Table S2d). (**D**) A Venn diagram shows the overall 11,259 phosphosites deposited in the repository database of EPSD and 8087 phosphosites identified from this study (Supplementary Table S2e). These phosphosites were derived from the 3889 leading proteins (Supplementary Table S2g). (**E**,**F**) Classification of 8087 phosphorylation sites from the present study (**E**) and 11,259 phosphorylation sites from the Eukaryotic Phosphorylation Sites Database (EPSD, http://epsd.biocuckoo.cn/, accessed on 15 August 2020) (**F**) into the three general categories according to the amino acid sequence specificity of a kinase docking site (Supplementary Table S2e). (**G**) The number of the leading proteins identified from this study (3889), which include both unique and razor proteins (Supplementary Table S2g) and the EPSD database (3747). The red- (2457), green- (2315), and yellow- (1432) colored part represents the number of proteins specific to this study, proteins specific to EPSD database, overlapping phosphoproteins between this study and EPSD, respectively. (**H**) The simulated Tree-ring plot [72] represents the relationship between the number of phosphoproteins and the number of phosphosite. The area of each tree ring represents the number of phosphoproteins sharing the same number of phosphosite, whereas the color palette stands for various number of phosphosites on a phosphoprotein. The purple-colored ring 1 to 4 represents the phosphosite number per phosphoprotein varies from 1 to 4, while the blue-colored ring 5 to 17 represents the phosphosite number per phosphoprotein varies from 5 to 17. The green-colored represents those phosphoproteins of phosphosites larger than and equal to 18. The data used in this figure are from the unique phosphoprotein data of this study and those of the EPSD database (Supplementary Table S2i). (**I**) The power law equation of the fitting curve describes the correlation in between the total number of phosphoproteins (4694) and the specific number of phosphosite (in total). *R*^2^ represents the coefficient of the curve determination. The data used in this figure are from the unique phosphoprotein data of this study (1808) and those of the EPSD database (3747, Supplementary Table S2i).

The isotopically labeled phosphopeptides were identified using the Mascot search engine according to methods previously described (see Materials and Methods for details; Figure S3; [46,47]). The intermediate PTM proteomic analysis results are summarized in Figure S4 and listed in the Supplementary Table S2a–c. After removing the influence of the dimethyl labeling on the peptide identification, we have obtained a total of 12,824 non-repeatable (PSM > 1) phosphopeptides and 9457 repeatable phosphopeptides (PSM > 1; Figure S4A; Supplementary Table S2d). Only these repeatable phosphopeptides are used for the bioinformatic analysis in this paper. Although the number of phosphopeptides discovered is positively correlated to the number of experimental replicates (Figure S4B), however, only 2–3% increment of phosphopeptides discovery rate was achieved from the fourth to sixth experimental replicates, suggesting a lower cost effectiveness beyond the third experimental replicate. The distribution of the number of phosphopeptides over the length of detected phosphopeptides indicated that a majority (90.6%, 7662) of phosphopeptides had a sequence length ranging from 7 to 25 amino acids long (Figure S4C; Supplementary Table S2d), which are consistent with what we expected from the in-silico digestion of these *Arabidopsis* proteins, suggesting a successful trypsin digestion of the total soybean protein from both cultivars. Among these 9457 phosphopeptides, 1080 phosphopeptides were found to be drought-tolerant cultivar-specific while 1079 phosphopeptides drought-sensitive cultivar-specific under water deprivation treatment (Figure 1A; Supplementary Table S2d). These cultivar-specific phosphopeptides may result either from the variation in genotypes of two cultivars or from the combinatorial effects of both genotypes and drought treatment.

The relationship between the frequency of PSMs of each phosphopeptide and the ranking of the 9457 phosphopeptides can be described by Zipf’s law with a slope of –0.91 (Figure 1B; Supplementary Table S2d) as does the *Arabidopsis* proteome [59]. The rate of slope reflects the combinatorial state of the extent of protein phosphorylation and the coverage of phosphoproteomics. Further investigation of the phosphopeptides reveals that there are 7950 singly, 1305 doubly, 197 triply, and 5 quadrupled phosphorylated peptides. (Figure 1C; Supplementary Table S2d). As the primary sequence of one phosphopeptide may be shared by several deduced soybean proteins, the corresponding phosphoprotein is therefore defined as protein groups regardless of the “protein group” contains either one or more individual proteins. A leading protein from each protein group was subsequently selected using Percolator [73,74]. On the other hand, the tryptic digestion of a phosphoprotein may generate numerous phosphorylated phosphopeptides due to the presence of the multiple phosphosites on a phosphoprotein. It was therefore that these 9457 phosphopeptides (Supplementary Table S2d) were eventually found to derive from 4571 protein groups (Supplementary Table S2f), among which there exist 3889 leading proteins (Supplementary Table S2g). In fact, 1808 of leading phosphoproteins (Supplementary Table S2h) came from protein groups containing a single unique phosphoprotein while 2081 leading proteins were from protein groups containing more than one phosphoprotein (Supplementary Table S2f).

Further analysis showed that these 3889 leading phosphoproteins contain a total of 8087 phosphosites (Supplementary Table S2e), among which 6106 of phosphosites have not been reported before as compared to 11,259 phosphosites deposited in the Eukaryotic Phosphorylation Site Database (EPSD, http://epsd.biocuckoo.cn/index.php, accessed on 15 August 2020; [75]) (Figure 1D; Supplementary Table S2e). These novel phosphosites increases the current soybean phosphosite repository 54%. Analysis of the substrate docking sequence specificity of Ser/Thr protein kinases on these 8087 phosphosites revealed that 40% (3235), 12% (970), and 24% (1941) of these phosphosites have acidophilic (p[S/T][D/E] or p[S/T]xx[D/E]), basophilic (Rxxp[S/T]), and proline-directed (p[S/T]P) motifs, respectively (Supplementary Table S2e). Although the basophilic and proline-directed motifs show similar percentages to those deposited in EPSD database, i.e., 13% (1464) in basophilic and 23% (2590) in proline-directed motifs, the acidophilic motifs of these newly measured phosphosites (40%, 3235) showed a 5% difference from that deposited in the EPSD database (45%, 5066).

When 3889 of leading phosphoproteins identified from this study (Supplementary Table S2g) were compared with those phosphoproteins deposited in the EPSD database, there were 1432 of overlapping phosphoproteins and 2457 phosphoproteins unique to this study (Figure 1G), which increases the size of soybean phosphoprotein database 65.6%. Among those 3889 leading proteins, 65.32% (2540) proteins have a molecular weight ranging from10 to 50 kDa (Figure S4D; Supplementary Table S2g).

To model the relationship between phosphosite and phosphoprotein mathematically, we selected 1808 unique phosphoproteins from this study (Supplementary Table S2h) and those unique phosphoproteins from the EPSD database (Supplementary Table S2i) to assimilate an equation. As a result, we found that there were 1990 phosphoproteins containing one phosphosites while the largest number of phosphosite present on a single protein (Nucleolin 1) is 62 (Figure 1H; Supplementary Table S2i). Curve fitting of the subgroup population distribution of phosphoproteins against the number of phosphosite on phosphoprotein produced an exponential decay equation (Y = 3.89e^−0.08X^ − 0.27) (Figure 1I; Supplementary Table S2i). The correlation between the number of phosphosite and the number of serine, threonine and tyrosine residues in these phosphoproteins (Figure S4E; Supplementary Table S2i), the protein length of phosphoprotein (Figure S4F; Supplementary Table S2i) and the protein mass of phosphoproteins (Figure S4G; Supplementary Table S2i), were calculated, respectively. All of these results showed a Pearson correlation coefficient within 0.14 and 0.16, suggesting that there is no correlation between the number of measured phosphosites and the subgroup population of phosphoproteins classified according to the number of their Ser/Thr/Tyr contents, their lengths and masses. The biological impact of the modeling equation is unclear at this stage.

To understand the molecular functions of those overly post-translationally-modified proteins (OPP), which was defined as such that an OPP should contain more than 10 phosphosites, the 3889 leading proteins (Supplementary Table S2g) were further classified into 3 subgroups according to the number of phosphosite on each phosphoprotein. Group A, B and C contains phosphoproteins of 1–4 phosphosites, 5–10 phosphosites and OPPs, respectively. The identifiers of three subgroups of phosphoproteins together with the total phosphoproteins were converted to *Arabidopsis* orthologs, which were subsequently subjected to the Gene Ontology analysis (GO; see Material and Methods for details). Similar GO enrichments were observed among the total phosphoprotein (T), Group A and B, whereas distinct GO enrichments were observed in between OPPs and the rest (Figures S5 and S6). By the molecular function enrichment, the OPPs were found to be mostly enriched in phosphatase/kinase including protein tyrosine phosphatase, protein serine/threonine/tyrosine kinase and photoreceptor kinase, and in enzyme activities, including phosphoenolpyruvate carboxylase and sucrose-phosphate synthase, as well as in the binding events including mRNA-, histone-, clathrin-, phosphatidic acid-, and phospholipid-binding (Figure S5). By the biological processes classification, the functions of OPPs were enriched to the nuclei-related processes including chromatin remodeling, chromatin assembly, regulating of RNA splicing, production of miRNAs involved in gene silencing, primary miRNA processing, and regulation of double-strand break repair (Figure S6A). By the cellular component classification, we also observed the highly enrichment of OPPs in the categories of nuclear subunits that contained nuclear dicing body, nucleoplasm, nuclear body, U2-type spliceosome complex and chromatin. These findings suggested these OPPs may play an important role in nuclear activities and mRNA processing events, both of which contribute to the regulation of gene expression (Figure S6B).

### 3.3. Mapping the Putative Kinase-Substrate Interactome

**Figure 2:**
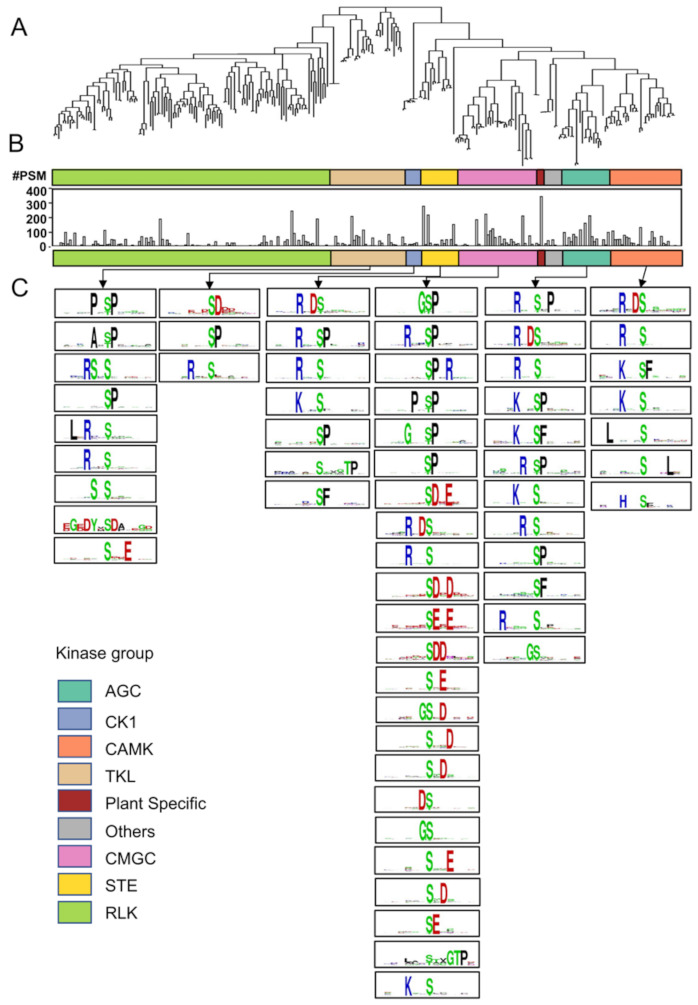
Overview of kinase and substrates under drought tolerance. (**A**) The dendrogram of kinase families were generated by MEGAX (Molecular Evolutionary Genetics Analysis across Computing Platforms [61]). The colored boxes show the classification of the phosphorylated kinases identified from soybean in this study (Supplementary Table S2g) using the software iTAK (iTAK (http://itak.feilab.net/cgi-bin/itak/index.cgi, accessed on 7 April 2021, Supplementary Table S3a). AGC, cAMP-dependent protein kinase, cGMP-dependent protein kinase and protein kinase C family; CK1, casein kinase I family; CAMK, Ca^2+^/calmodulin-dependent protein kinase family; TKL, tyrosine kinase-like kinase family; The Plant Specific represents plant-specific kinase family; CMGC, the kinase family of cyclin-dependent kinase (CDK), mitogen-activated protein kinase (MAPK), glycogen synthase kinase (GSK) and CDC-like kinase; STE, sterile kinase family; RLK, receptor-like kinase family; Others represents a group of other kinase families instead of those mentioned above. (**B**) The bar plot represents the PSM count of each kinase among kinase families (data were generated by SQUA-D, Supplementary Table S3a, [46,47]). (**C**) The lowest panel shows the motifs (61 in total) of each kinase family found from this study using both the software Motif-X (Version 1.0) and Motif-All (Version 1.2, Supplementary Table S3f, [64,65]). The details of the data processing are described in Material & Methods and Figure S7.

Out of 3889 leading proteins (Supplementary Table S2g), we firstly identified 279 annotated kinases. These kinases were classified into 9 kinase families (Figure 2A and Figure S4; Supplementary Table S3a; Supplementary Table S4) using the bioinformatics tool of iTAK (Plant Transcription factor and Protein Kinase Identifier and Classifier; [60]), among which RLK family has the largest number of kinase members (120), followed by CMGC (34), TKL (32), CAMK (31), AGC (20), STE (15), others (9), CK1 (7), and plant-specific (4) kinase families (Supplementary Table S3a).

By counting the PSM of individual constituent kinase from each kinase family using an in-house made software SQUA-D [46,47], we found that there were three kinase families, i.e., plant-specific kinase, AGC, and CMGC, which have an average of PSM count of 95.5, 60.2, and 46.3, respectively (Figure 2B; Supplementary Table S3a). In fact, it is the GmSTN7 (State transition 7) kinase, a member of the plant-specific kinase family, that has the largest number of PSMs (344) among all these putative kinases identified (Figure 2; Supplementary Table S3a). Similarly, GmPHOT2 (phototropin-2), GmCKL6 (casein kinase 1-like protein 6), GmCRK5 (CDPK-related kinase 5-like), GmDDB_G0282963 (probable serine/threonine-protein kinase), GmCDKG-2 (cyclin-dependent kinase G-2-like), GmBLUS1 (blue light signaling 1), GmRLK1 (receptor-like kinase 1) and GmWNK7 (with no lysine kinase 7) is the representative kinase having the largest number of PSM count in AGC, CK1, CAMK, TKL, CMGC, STE, RLK, and “other” kinase family, respectively (Figure 2; Supplementary Table S3a). These spectral counts data suggest that these kinase families or members are more abundant or have a higher protein phosphorylation level than the rest, assuming that the spectra counting (Supplementary Table S3a) is able to roughly estimate the level of a PTM protein [76]. These relatively more abundant and highly phosphorylated kinases may in turn have a higher kinase activity output under the drought condition.

To find the possible substrates of these putative kinases, all 8087 phosphosites were converted into 13-mer oligopeptide sequences with the phosphorylated amino acid located at the center (Supplementary Tables S2e and S3b). Consequently, the putative kinase families corresponding to these phosphosite-containing substrates were predicted using a GPS software (Group-based Prediction System; [62]), resulting in 81,402 substrate–kinase family pairing relationships (one phosphosite could be mapped to multiple kinase families), which contain 10 kinase families, AGC, Atypical, CAMK, CK1, CMGC, Dual, STE, TK, TKL, and other (Supplementary Table S3b).

Since this soybean phosphoproteomics had identified nine putative kinase families (Figure 2A), merging with the GPS-predicted 10 kinase families subsequently resulted in 20,357 substrate–kinase family pairing relationships and six overlapping kinase families, AGC, CK1, CAMK, TKL, CMGC, and STE (Figure 2B,C and Figure S7; Supplementary Table S3c). The substrates–kinase pairs within these six kinase families were then filtered by the protein-protein interactions recorded either in STRING (Search Tool for the Retrieval of Interacting Genes/Proteins; [63]) or in BioGRID (Biological General Repository for Interaction Datasets; https://thebiogrid.org/, accessed on 8 September 2020) database. As a result, 4227 substrate–kinase family relationship pairs were eventually obtained for the members of these six kinase families (Figure S7; Supplementary Table S3d), among which AGC, CK1, CAMK, TKL, CMGC, and STE kinase family contains 692, 167, 412, 757, 1935, and 264 substrates, respectively (Figures S7 and S8; Supplementary Table S3d). Furthermore, these 4227 substrate–kinase family relationship pairs can be extended into 20,550 substrate–kinase pairs (protein-protein interactions; Supplementary Table S3e).

The phosphosite motifs (Figure 2C and Figure S7; Supplementary Table S3f) were subsequently generated among the phosphosite substrates of each of the six kinase families using Motif-ALL (Version 1.0) and Motif -X software (Version 1.2) [64,65]. Consequently, 12, 3, 7, 9, 23 and 7 different phosphosite motifs were enriched for AGC, CK1, CAMK, TKL, CMGC, and STE kinase family, respectively (Figure 2C; Supplementary Table S3f).

### 3.4. Quantitative Phosphoproteomic and Bioinformatic Analysis of the Regulated Phosphoproteins

**Figure 3:**
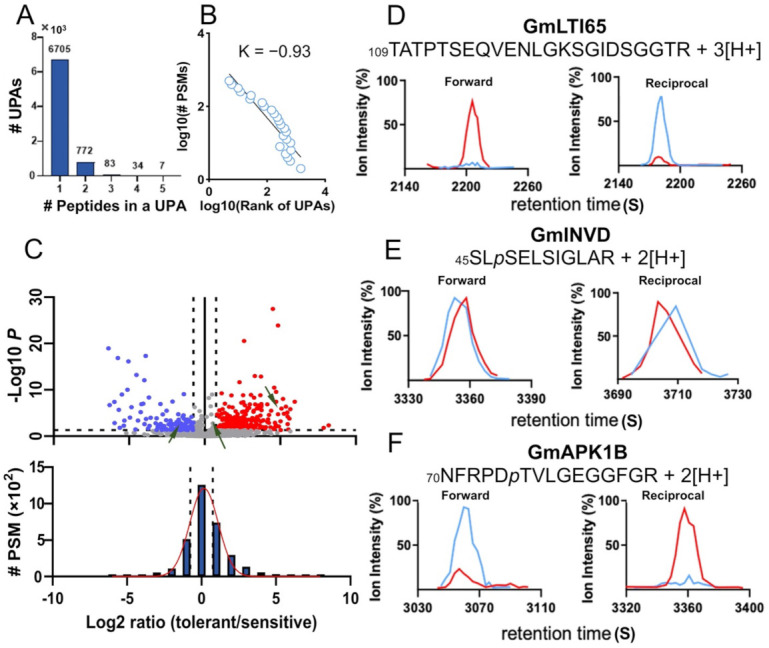
Quantitative proteomic analysis of the drought-tolerant soybean cultivar significantly regulated phosphopeptide groups under drought treatment. (**A**) Distribution and classification of phosphopeptide groups (or called UPAs, unique PTM peptide array, 7601 in total) according to the number of phosphopeptides present an UPA (Supplementary Table S5a). (**B**) The rank-frequency distribution of the number of PSMs over the index of UPAs (7601) according to Zipf’s law (Supplementary Table S5a). (**C**) Volcano plot (upper) and histogram (lower) of quantitative phosphoproteomics. The Log2 ratio is the average binary logarithmic ratio of MS1 isotopologue areas of phosphopeptides, and *p* is the *p*-value determined from Student’s *t*-test. The histogram of Log2 ratios are fitted using a normal distribution (red curve). The vertical and horizontal dashed line indicates the mean ± SD (standard deviation) of the distribution and the cut-off of Benjamini–Hochberg multiple hypothesis test corrected FDR (≤0.05), respectively (Supplementary Table S5b). (**D**–**F**) The red and blue circle represents significantly up- and downregulated UPAs, respectively. (**D**–**F**) shows the XIC of increased, unchanged and decreased phosphopeptide ratio of drought-tolerant sample to the drought-sensitive sample, respectively. Forward and Reciprocal stands for the experimental replicate of F and R mixing of differentially isotope-labeled total cellular peptides, respectively. Subscript p marks the phosphosite within a measured phosphopeptide. Red lines represent the heavy isotope-labeled phosphopeptides while the blue lines represent the light isotope-labeled phosphopeptides. The number at the N-terminal end of phosphopeptide indicates the amino acid position within a phosphoprotein. The phosphosite and XIC shown in (**D**–**F**) are reproduced from soybean protein GmLTI65 (low-temperature-induced 65 kDa Protein, XP_003556105.1-S123), GmINVD (alkaline/neutral invertase D, XP_003536372.1-S47) and GmAPK1B (protein kinase 1B, XP_003523529.1-T75) listed in the Supplementary Table S5b, respectively.

Due to the incomplete proteolytic digestion of proteins and the existence of isoforms of a protein, various lengths of PTM peptides sharing the same PTM site are frequently produced in peptide samples. The concept of unique PTM peptide array (UPA) was introduced to describe the peptide group that share the same PTM site or the unique PTM site pattern (UPSP; [46,47]). Thus, the 9457 repeatable phosphopeptides (Supplementary Table S2d) were consequently converted into 7601 UPAs (Supplementary Table S5a), in which the majority of UPAs (6705 out of 7601, 88%) contained only one phosphopeptide while 7 UPAs consisted of 5 phosphopeptides (Figure 3A; Supplementary Table S5a). The PSM counts of UPAs distributed against each of 7601 phosphopeptide groups (Supplementary Table S5a) still obeys the Zipf’s law, and the combination of these phosphosites to a UPA slightly changed the exponent of the power-law fitting from −0.91 to −0.93. (Figure 1C and Figure 3B). Out of these 7601 UPAs, 2538 (Supplementary Table S5b) were selected as the quantifiable UPAs (see Material and Methods for the selection criteria) and quantified by SQUA-D software [46,47]. As a result, 260 and 118 phosphopeptide groups (or UPAs) were found to be the tolerant cultivar significantly (BH-FDR ≤ 0.05) up- and downregulated, respectively, under drought treatment (Figure 3C; Supplementary Table S5b). The number of drought-tolerant cultivar significantly regulated phosphoproteins were further trimmed according to an empirically determined standard (see Materials and Methods).

In the end, we have determined 218 drought-tolerant cultivar significantly regulated UPAs, corresponding to 188 protein groups, among which there were 145 UPAs derived from the significantly upregulated 120 phosphoprotein groups, whereas 73 UPAs derived from 68 significantly downregulated protein groups (Figure 3D–F; Supplementary Table S6a). Moreover, 87 out of 188 (46%) phosphoprotein groups were found to be novel in relation to drought tolerance, among which 51 out 87 (59%) and 36 out of 87 (41%) were up- and downregulated by drought-tolerant cultivar, respectively (Supplementary Table S6a). Further analysis of phosphoprotein subcellular localization revealed that in the drought-tolerant cultivar-enhanced phosphoprotein groups, 49 (41%), 27 (22%), 19 (16%), 12 (10%), and 13 (11%) of which locate to nucleus, cytosol, plasma membrane, plastid and others (Golgi, vacuole, mitochondrion, extracellular, endoplasmic reticulum, and peroxisome), respectively. In contrast, there were 31 (46%), 14 (20%), 11 (16%), 4 (6%), and 8 (12%) drought-tolerant cultivar significantly suppressed phosphoproteins locating in nucleus, cytosol, plasma membrane, plastid and others (endoplasmic reticulum, vacuole, Golgi, mitochondrion and extracellular), respectively (Supplementary Table S6a).

Among these SRPs (significantly regulated phosphoproteins; Supplementary Table S6a), the GmLTI65 protein, a low-temperature-induced protein 65 kDa, is an ortholog of the *Arabidopsis* abscisic acid (ABA)-responsive protein RD29B (Responsive to Desiccation 29B). It was found to have a total of twelve phosphosites (Supplementary Table S6a), including S123 site, some of which were coordinately enhanced at the phosphorylation level under drought treatment. Together with this overly post-translationally modified protein (OPP), there were nine kinases/phosphatases (GmBLUS1, GmSRK2I, GmBSL1, GmCIPK25, GmPERK1, GmAKINb1, GmMKKK, GmSTN7, GmMKK4) and 10 phosphorylated transcription factors (GmHB1, GmHB12, GmILP1, GmGRP2, GmNAP1;2, GmTPL, GmPAT1, GmSPEN2, GmATRX, GmPDS5A) being upregulated in the drought-tolerant cultivar under drought treatment (Supplementary Table S6a). In contrast, there were 8 kinases/phosphatases (GmRAF20, GmLYK4, GmPK, GmPP2C, GmLRR-RKs, GmKinX, GmNFs, GmAPK1B) and five transcription factors (GmARID5, GmTRP3, GmCOP1IP, GmTCF25 and GmSPT6) being downregulated by drought-tolerant cultivar (Supplementary Table S6a). These findings suggest a complex phosphor-relay signaling network mediating a diverse transcription activity that might be involved in the soybean drought tolerance.

In fact, by the quantitative phosphoproteomics analysis, 9 out of 15 (60%) of transcription factors (GmILP1, GmNAP1;2, TPL, HB1, GmSPEN2, GmPDS5A, GmARID5, GmCOP1IP, and GmSPT6) and 5 out of 17 (29%) of kinases/phosphatases (GmBLUS1, GmBSL1, GmKinX, GmNFs, and GmAPK1B) were found for the first time to be involved in drought tolerance (Supplementary Table S6a). In addition, there were 16 nuclear regulators (GmSR45a, GmGCT, GmFREE1, GmEIFSO4G1, GmG3BP, GmMET, GmSERBP1, GmEIN2, GmRSZ32, GmKHDR8, GmRH20, GmC3H17, GmAML5, GmBTR1, GmCBE1, and GmEFTs) being upregulated in the drought-tolerant cultivar, eight of which (53%) were in fact novel drought-related phosphoproteins. On the other hand, we also found that 18 nuclear regulators (GmLIG1, GmCMT2, GmDEF1, GmUBA2B, GmDRP, GmDDX31, GmSYF1, GmASIL2, GmRING1, GmHMT, GmIDM, GmRH32, GmC3H13, GmEIF4E1, GmRPM1, GmEF1B, GmRAD21.3, and GmSCL30) being downregulated by drought-tolerant cultivar (Supplementary Table S6a), 12 of which (75%) were found for the first time to be drought tolerance-related.

Moreover, four (GmPIP2;2, GmPIP2;1, GmPIP2-7, and ABCG22) and three (GmDEP1, GmARD2, and GmCIMS) of the upregulated phosphoproteins were involved in water transport process and methionine metabolic process, respectively (Supplementary Table S6a). Among the photosynthesis system-related phosphoproteins (GmLHCB1.3, GmLHCB4.2, GmLHCB2, GmPSBR, GmPSBS, GmPSBQ, and GmLHCB6), which were all upregulated, GmLHCB4.2 was discovered for the first time to be associated with drought tolerance (Supplementary Table S6a). Eleven of the upregulated phosphoproteins (GmSRK2I, GmCDSP32, GmLTI65, GmHB-12, GmDI8, Gm PIP2;1 GmLHCB2, GmPIP2;2, GmABCG22, GmCOR47, and GmCCYS6) were related to water deprivation (Supplementary Table S6a). Apart from these, nine upregulated phosphoproteins (GmCYS-3A, GmGPX6, GmAMT, GmNAP1;2, GmDTSP3, GmGST8, GmDTSP4, GmTUA5, and GmCIMS) were found to responsive to cadmium ion. It was interesting to find that GmNAP1;2, GmDTSP3, GmGST8, and GmDTSP4 phosphoproteins were found to be associated with the drought tolerance for the first time (Supplementary Table S6a). Twelve (GmAMPD, GmDI8, GmHB-12, GmLTI65, GmP5CS2, GmSRK2I, GmCOR47, GmPIP2;2, GmGMP, GmLOX1, GmPIP2;1, and GmPIP2-7) and 20 (GmDI8, GmHB-12, GmLTI65, GmCDSP32, GmCYS3A, GmGPX6, GmSRK2I, GmAMT, GmNAP1;2, GmCYS6, GmCOR47, GmDTSP3, GmABCG22, GmGST8, GmPIP2;2, GmDTSP4, GmTUA5, GMCIMS, GMLHCB2, and GmPIP2;1) of the upregulated phosphoproteins were classified into the abscisic acid response and inorganic substance response, respectively. Among them, both GmDTSP3 and GmDTSP4 proteins were novel drought-tolerance-related phosphoproteins (Supplementary Table S6a). Furthermore, nine osmotic stress response proteins (GmLTI65, GmP5CS2, GmSRK2I, GmCYS6, GmCOR47, GmEIN2, GmDTSP4, GmNCL, and GmREM) were upregulated, among which both GmDTSP4 and GmNCL were novel (Supplementary Table S6a). Sixteen oxygen-containing component response proteins (GmAMPD, GmDI8, GmHB-12, GmLTI65, GmCNX1, GmP5CS2m GmSRK2I, GmCOR47, GmTPL, GmPEX5, GmBSL1, GmEIN2, GmGMP, GmLOX1, GmPIP2;1, and GmPIP2-7) were up-regulated. Among them, GmTPL and GmBSL1 were newly identified drought-tolerant phosphoproteins (Supplementary Table S6a). There were 22 upregulated phosphoproteins (GmAMPD, GmDI8, GmHB-12, GmLTI65, GmCDSP32, GmP5CS2, GmSRK2I, GmCYS6, GmREM1.2, GmCOR47, GmTPL, GmABCG22, GmBSL1, GmPUB4, GmPIP2;2, GmEIN2, GmGMP, GmLOX1, GmREM, GmLHCB2, GmPIP2;1, and GmPIP2-7) being classified into hormone response, among which the phosphorylation of GmREM1.2, GmTPL, GmBSL1, and GmPUB4 phosphoproteins was found for the first time to be associated with the drought-tolerance (Supplementary Table S6a).

**Figure 4:**
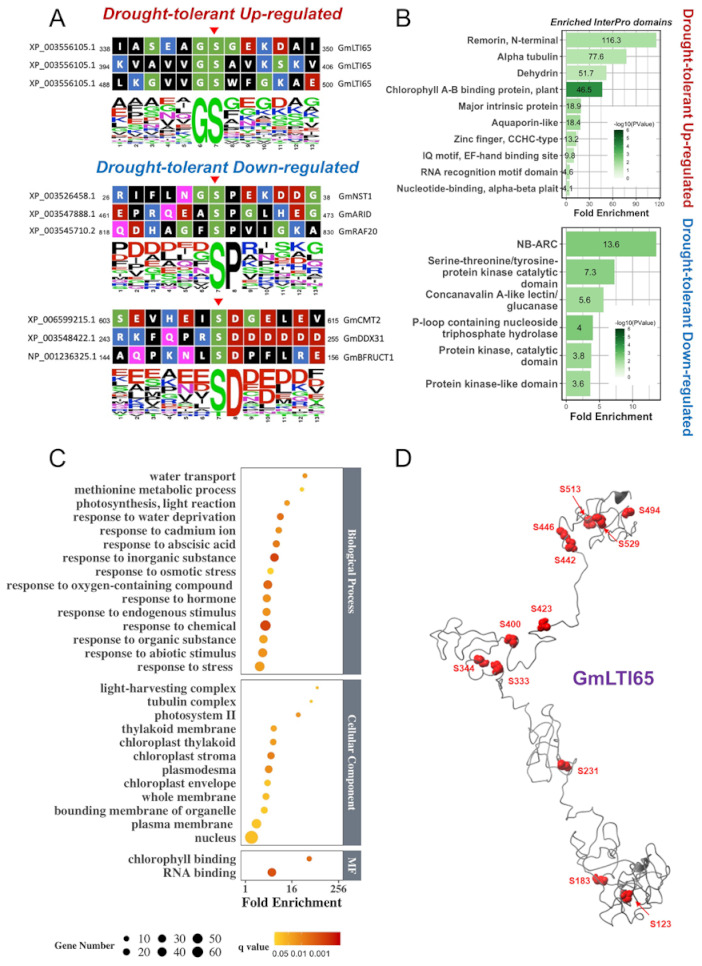
Bioinformatic analysis of the drought-tolerant cultivar significantly regulated protein phosphorylation. (**A**) Phosphosite motifs constructed using the Motif-X and Motif-All [64,65] from the highly selected and the significantly regulated phosphopeptide groups (Supplementary Table S6a). (**B**) The InterPro domains enriched from significantly up- (upper panel) and downregulated (lower panel) leading phosphoproteins (Supplementary Table S6a). The number in each bar indicates the fold enrichment (observed vs. expected). The color in each bar indicates the negative logarithm of *p* value. (**C**) GO enrichment analysis of the significantly regulated phosphoproteins (Supplementary Table S6a). The biological processes, cellular component and molecular function of significance (FDR ≤ 0.05) are shown. The color of dot indicates the significance of the enrichment from 0.05 (yellow) to 0.00000001 (deep red). The size of the dot represents the number of phosphoproteins in a specific GO category. (**D**) The predicted 3D structure of the overly phosphorylated protein (OPP) GmLTI65 using software I-Tasser (Version 5.1) [66] with the phosphosites indicated by red dots.

The phosphosites of the significantly regulated phosphopeptide groups are converted into the 13-mer oligopeptide sequences and analyzed using Motif-ALL (Version 1.0) and Motif -X software (Version 1.2) [64,65], resulting in an enrichment of a GpS motif in the drought-tolerant cultivar and pSD and pSP motifs in the drought-sensitive cultivar (Figure 4A; Supplementary Table S6a). Interestingly, the drought-tolerant cultivar-specific GpS motif comprised of 3 of the 12 unique phosphosites discovered from GmLTI65 protein (Figure 4B). The phosphorylation levels of these phosphosites increased to 2.3- to 5.5-fold in drought tolerant-cultivar over the drought-sensitive cultivar upon drought treatment (Supplementary Table S6a). Based on the structural prediction of GmLTI65 phosphoprotein (see Material and Methods), these phosphosites are clustered in three regions: (1) the N-term region containing two phosphosites (S123 and S183); (2) the middle region containing five phosphosites (S231, S333, S344, S400, and S423); and (3) the C-terminal region containing five phosphosites (S442, S446, S494, S513, and S529) (Figure 4B; Supplementary Table S6a). The three phosphosites comprising the drought-tolerant-specific GpS motif (S344, S400, and S494) locate in both the middle and the C-terminal region.

To find the functional domains of these drought-tolerant cultivar significantly regulated phosphoproteins (Supplementary Table S6a) via bioinformatics, these soybean SRPs (significantly regulated phosphoproteins) were first converted into the corresponding *Arabidopsis* orthologs using the online BLAST (https://blast.ncbi.nlm.nih.gov/Blast.cgi, accessed on 4 May 2020). Consequently, these corresponding *Arabidopsis* orthologs (Supplementary Table S6a) were searched against the protein domain database deposited in the InterPro database (https://www.ebi.ac.uk/interpro/, accessed on 5 May 2020). It was found that the N-terminal domain of remorin, alpha-tubulin domain, dehydrin domain, chlorophyll A/B binding protein domain, and major intrinsic protein domain were enriched as the top five domains for the drought-tolerant cultivar, whereas NB-ARC (nucleotide-binding adaptor shared by APAF-1, R proteins, and CED-4) domain, serine-threonine/tyrosine-protein kinase catalytic domain, concanavalin A-like lectin/glucanase domain, P-loop-containing nucleoside triphosphate hydrolase domain and protein kinase catalytic domain were the five highly enriched in the drought-sensitive cultivar (Figure 4C).

Gene ontology (GO, http://geneontology.org/, accessed on 5 May 2020) enrichment was performed on these SRPs (Supplementary Table S6a) to reveal the biological relevance (Figure 4D). Both drought-tolerant cultivar significantly up- and down-regulated phosphoproteins were used for the GO analysis. To our surprise, not a single category of protein was enriched for the drought-tolerant downregulated phosphoproteins. As a result, in the drought-tolerant cultivar up-regulated phosphoproteins, both chlorophyll-binding and RNA-binding phosphoproteins were enriched under the molecular function, the light-harvesting complex, tubulin complex, photosystem II and thylakoid membrane, and chloroplast thylakoid enriched as the top five categories under the cellular component, and finally response to various stimuli and stresses (e.g., water deprivation, cadmium ion, abscisic acid, osmotic stress, hormone and chemical etc.) were significantly enriched within five categories of biological processes. Taken together, this quantitative phosphoproteomics study has found that the phosphorylation level of phosphoproteins related to water transport, methionine metabolic process, photosynthesis/light reaction, response to water deprivation, and response to cadmium ion were significantly enhanced in the drought-tolerant cultivar under water deprivation treatment (Figure 4D).

### 3.5. Transcriptional Analysis of the Regulated Soybean Phosphoproteins

Soybean genes encoding these significantly regulated phosphoproteins were randomly selected to study their drought-induced transcriptional activities using the RT-qPCR analysis (Figure S10A–H; Supplementary Table S7). The purpose of this study is to confirm the measured phosphorylation alteration occurring on so many soybean phosphoproteins result from the enhancement in both gene expression and post-translational modification by kinases/phosphatases. These soybean phosphoprotein-encoding genes are *GmGRP2*, *GmDEP1*, *GmP5CS2*, *GmLTI65*, *GmPPDK*, *GmAPK1b*, *GmSR45a*, *GmHOS1*, and *GmCHUP1*. The total cellular RNA samples were isolated from both drought-tolerant and -sensitive plant tissues at four time points of drought treatment, day 6, day 8, day 10, and day 12. Quantitative reverse transcription polymerase chain reaction (RT-qPCR) was performed on the RNA samples. *GmGRP2* gene, as a member of CSD transcription factor family, has been reported to respond to cold and salt stress in plants [77]. The ratio of the relative transcript expression level of *GmGRP2* in between the drought-sensitive and the drought-tolerant cultivar was 0.61 ± 0.16 vs. 4.94 ± 0.92, 1.63 ± 0.21 vs. 2.42 ± 0.63, 1.03 ± 0.17 vs. 3.75 ± 0.58 and 0.98 ± 0.12 vs. 2.94 ± 0.17 on day 6, day 8, day 10, and day 12 of drought treatment, respectively (Figure S10A; Supplementary Table S7). As to the *GmDEP1* gene, which was reported to affect osmotic stress resistance in *Arabidopsis* [78], the relative transcript expression of this gene was higher in the drought-tolerant cultivar under drought treatment at all four time points (0.68 ± 0.12 vs. 1.98 ± 0.18 at day 6, 0.91 ± 0.1 vs. 2.00 ± 0.23 at day 8, 1.46 ± 0.18 vs. 4.81 ± 0.46 at day 10, and 1.59 ± 0.10 vs. 5.62 ± 0.64 at day 12, Figure S10B; Supplementary Table S7). In the case of *GmP5CS2* gene, which was rapidly induced by drought and salt stress with abscisic acid as the key transducing signal [79,80], the ratio of relative transcript expression level in between the drought-tolerant and -sensitive cultivar was 1.05 ± 0.23 vs. 2.32 ± 0.10, 1.15 ± 0.10 vs. 13.72 ± 1.72, 23.43 ± 5.39 vs. 575.92 ± 131.06 and 61.34 ± 16.53 vs. 587.59 ± 116.90 at day 6, day 8, day 10, and day 12, respectively, under drought treatment (Figure S10C; Supplementary Table S7). Similarly, the *GmLTI65* gene is known to be abscisic acid-dependent and drought-inducible [81]. The pattern of its relative transcript expression is similar to that of GmP5CS2, i.e., 0.67 ± 0.04 vs. 6.74 ± 2.10, 7.61 ± 1.61 vs. 863.64 ± 99.62, 1153.01 ± 35.40 vs. 5314.56 ± 283.76, and 1834.84 ± 191.88 vs. 5367.18 ± 424.47 at day 6, day 8, day 10, and day 12, respectively, under drought treatment (Figure S10D; Supplementary Table S7). Moreover, the phosphorylation level of kinase GmAPK1B was measured to be down-regulated in the drought-tolerant cultivar whereas its RT-qPCR result seemed to be consistent with the quantitative phosphorylation data, in which a significant reduction in the transcript level on day 10 and day 12 was found in the drought-tolerant cultivar as compared to that of the drought-sensitive cultivar under treatment (i.e., 14.78 ± 1.83 vs. 5.91 ± 1.00 and 9.25 ± 0.46, and 4.43 ± 0.39; Figure S10E; Supplementary Table S7). Apart from the above five genes, the transcription level of which is consistent with the phosphorylation level of theirs, there are another three genes, *GmSR45a*, *GmHOS1*, and *GmCHUP1*, which have different expression patterns (Figure S10F–H; Supplementary Table S7). These three genes have no significant transcription differences between the drought-tolerant and -sensitive cultivars, suggesting that regulation of protein phosphorylation could either be a result of increase in gene expression or from the phosphorylation of the protein itself or both.

### 3.6. Bioinformatics Analysis of the Function and Network of the Regulated Phosphoproteins

**Figure 5:**
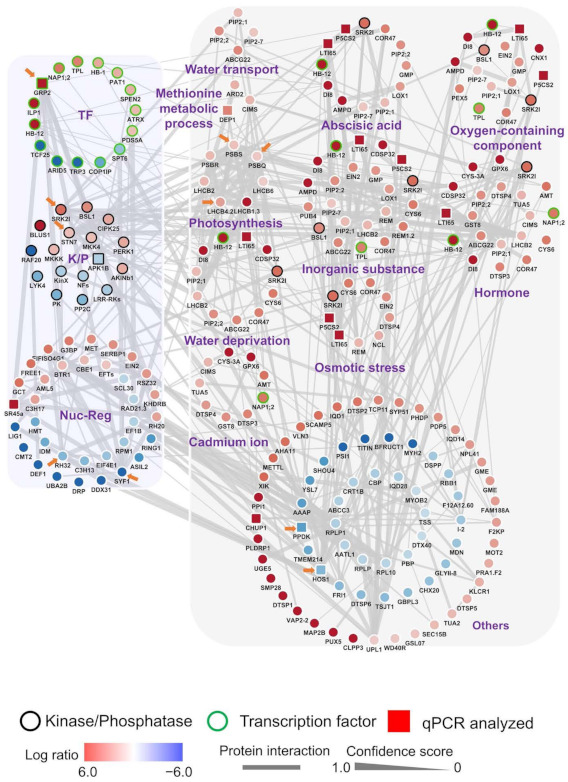
Volvox graphic representation of the putative protein-protein interaction. Both circular and squared node represents a significantly regulated phosphoprotein. The red and blue node stands for the significantly up- and downregulated phosphoprotein, respectively. The color palette indicates the level of protein phosphorylation (Supplementary Table S6a). The squared nodes represent those phosphoproteins analyzed by RT-qPCR (Figure S9). The nodes of black and green border stand for kinase/phosphatase and transcription factor, respectively. The edge represents a probable protein-protein interaction. The interaction is predicted by STRING database [63]. The thickness represents the confidence score (≥0.4) of interactions (Supplementary Table S6b). The groups of phosphoproteins of the same function classified according to GO analysis are depicted into Volvox colony-like circle(s). The leftmost three Volvox colonies represent three groups of regulatory phosphoproteins, whereas the rest Volvox colonies represent the functional groups with fold change larger than 3.57 and *p* value smaller than 0. The TF, K/P and Nuc-Reg represents transcription factor, kinase/phosphatase and nuclear regulator, respectively. The phosphoproteins enriched by multiple functional groups from GO analysis are dispersed simultaneously and in parallel into multiple Volvox colonies. The number of enrichments into each functional group is indicated in the Supplementary Table S6. The arrows point at the top five connected phosphoproteins of both regulatory and response phosphoproteins.

To identify the putative phosphor-relay networks in the drought-tolerant cultivar, the phosphorylation levels and the functional clustering results of the cultivar 120 upregulated and the 68 down-regulated phosphoproteins (Supplementary Table S6a) were subjected to both GO analysis (Supplementary Table S6a) and STRING [63] analysis (Figure 5; Supplementary Table S6b). As a result, we obtained a comprehensive functional interactome representing 248 nodes and 347 edges. Each individual gene encoding one of these phosphoproteins may have multiple repetitions in different gene function clusters (defined by GO analysis; Figure 5). During the analysis of the network (Figure 5; Supplementary Table S6), it is found that a few nodes have more edges connected than the average of the entire network (average = 2.8), indicating these network hubs may play key roles in regulating the drought tolerance in this cultivar. The regulatory phosphoproteins, GmSRK2I (or called GmSnRK2I), GmSTN7, GmGRP2, GmSYF1, and GmRH32, were found to have the highest connectivity while GmLHCB4.2, GmPSBS, GmHOS1, GmPSBQ, GmPPDK are the five top-ranking phosphoproteins in term of connectivity among phosphoproteins of response function (Figure 5).

**Figure 6:**
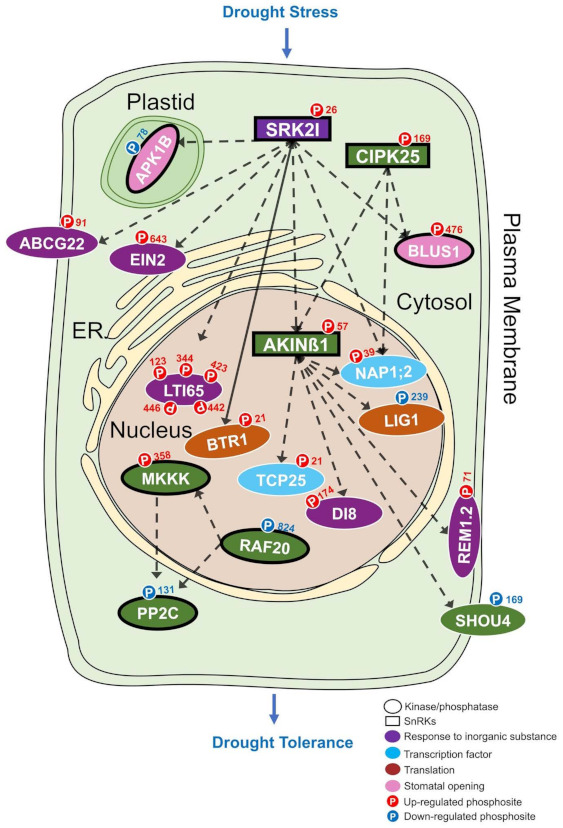
A model of phosphor-relay in the drought-tolerant cultivar under water loss. The double lines represent the plasma membrane consisting of bilayer lipids, which encloses the cytoplasm and some organelles, plastid, endoplasmic reticulum (ER), and nucleus. The colored ellipse and rectangle shaped nodes stand for various phosphoproteins. The circled small p and the numeric number beside represents the phosphoryl moiety and phosphosite on phosphoprotein, respectively. The red- and blue- colored circled p stands for up- and downregulated phosphorylation of the phosphosite, respectively. The purple, light blue, brown, and pink node represents candidates of proteins responsive to inorganic substance, transcription factor, proteins involved in translation and proteins regulating the stomatal opening, respectively (Supplementary Table S8). The black outline of the node marks kinase/phosphatase. APK1B, *Arabidopsis* protein kinase 1B; SRK2I, serine/threonine-protein kinase 2I; CPK25, calcium dependent protein kinase 25; ABCG22, ATP-binding cassette G22; EIN2, ethylene-insensitive 2; BLUS1, blue light signaling 1; AKINbeta 1, SNF1-related protein kinase 1; MKKK, mitogen-activated protein kinase kinase kinase; RAF20, rapidly accelerated fibrosarcoma 20; PP2C, protein phosphatase 2C. Black solid and dash arrow indicates the kinase –substrate relationship with the BioGRID and STRING record, respectively.

By integrating the significantly regulated phosphoprotein groups (SQUA-D; Supplementary Table S6a; [46,47]) with the substrates-kinase mapping (GPS 5.0; Figure 2; Supplementary Table S3d; [62]) and subcellular localization analysis (SUBA4; [71]), we are able to identify the regulated signaling pathways under drought stress (Supplementary Table S8). As a result, we have identified 18 signaling components from 24 substrates-kinase pairs (can be further combined into 20 phosphoprotein-kinase pairs), which contain one PP2C phosphatase and seven members of two kinase families, CAMK and TKL, as well as 10 members from the response function group (Figure 6 and Figure S7; Supplementary Table S8). In particular, the CAMK and TKL family contains 17 and 3 substrates-kinase pairs, respectively (Figure 6; Supplementary Table S8). Among these signaling components, 14 proteins contain drought tolerant cultivar up-regulated phosphosites, while four proteins contain down-regulated phosphosites (Figure 6; Supplementary Table S8). As predicted by SUBA4 [71], 9, 1, 4, 1, 3 of these proteins are located in nucleus, ER, cytosol, plastid and plasma membrane, respectively (Figure 6; Supplementary Table S8). According to the previously categorized four subgroups from the Gene Ontology analysis, six genes (GmSRK2I, GmABCG22, GmEIN2, GmLTI65, GmDI8, and GmREM1.2) are in response to inorganic substance, two proteins (GmTCP25 and GmNAP1;2) in transcription activity regulation group, two proteins (GmBTR1 and GmLIG1) in translation activity and two proteins (GmAPK1B and GmBLUS1) in stomatal opening (Figure 6; Supplementary Table S8). In fact, the pathway can be clustered into two distinct phosphor-relay network, one network contains GmSRK2I, GmCIPK25, GmAKINß1, GmAKP1B, GmBLUS1, GmABCG22, GmEIN2, GmLTI65, GmBTR1, GmTCP25, GmLIG1, GmNAP1;2, GmDI8, GmSHOU4 and GmREM1.2, whereas the other network contains GmMKKK, GmRAF20, and GmPP2C (Figure 6; Supplementary Table S8). Based on the degree of connectivity determined by Cytoscape [70], the three members of CAMK kinase family (Figure 2), GmAKINß1, GmSRK2I, and GmCIPK25 kinases, constitute major components of the phosphor-relay network during drought response, which are also members of plant SnRK1 (Snf1-related serine/threonine-protein kinase 1), SnRK2, SnRK3 subfamilies, respectively (Figure 6; Supplementary Table S8). Interestingly, these SnRKs are well-known major signaling components responsible for stress responses and metabolic adaption in plants [82].

## 4. Discussion

The distribution of the protein population abundance can be described by Zipf’s law, which is a discrete power law [83,84]. By Zipf’s law, a few protein species may have a large copy numbers while a large population of protein species have only a few copy numbers. However, in the case of PTM proteomes, we have previously reported that the correlation between the abundance of phosphoprotein and the number of phosphosite on phosphoprotein obeys a power law (Y(X) = a × X^−k^) based on the phosphoproteomics data collected from *Arabidopsis* [59]. However, in this soybean phosphoproteomics study, we found that this relationship between the abundance of a phosphoprotein and the number of phosphosite on a phosphoprotein is best fitted by an exponential decay law (Y = a × exp(−kX); Figure 1I) with a R square of 0.97, comparing with a power law fitting of a *R* square of 0.94. Moreover, the exponential decay law has a more precise fitness at the beginning point of the data in comparison with the power law (2094 vs. 7834 at first point, while the real data is 1990). Analysis of phosphoproteomic data deposited in the database (PhosphAT4.0, http://phosphat.uni-hohenheim.de/, accessed on 13 May 2020) revealed that this exponential decay equation is still suitable for describing the correlation between the abundance of a phosphoprotein and the number of phosphosite on a phosphoprotein in *Arabidopsis*. We hereby postulate that this exponential decay law (Y = a × exp(−kX); Figure 1I) may be applied universally to describe such a relationship for many different types of PTMs in a single organism or for the same PTM type in different organisms. The biological meaning of this exponential decay equation of the PTM protein population distribution over the PTM site number is still unknown so far. However, one argument claimed that it is the length and mass phosphoprotein and the number of serine, threonine, and tyrosine on phosphoprotein determines the exponential decay law. For that, we analyzed the correlation of the number of phosphosite over the phosphoprotein length (or mass) and the number of S, T, and Y sites and found that the correlation varies from 0.14 to 0.16 (Supplementary Figure S4), suggesting that the molecular weight of and the number of Ser, Thr and Tyr amino acids on a phosphoprotein do not affect the exponential decay law.

Bioinformatics analysis of the significantly up- and downregulated phosphoproteins (Supplementary Table S6a) by both Motif-X and Motif-All [64,65] has successfully identified both the G-S/T phosphosite motif and the S/T-P and S/T-D phosphosite motifs, respectively (Figure 4A). Based on the previously used bioinformatic method [58], it has been postulated that the G-S/T phosphosite motif may serve as a potential docking site for enzyme of AGC protein kinase family. Interestingly, both PHOT1 and PHOT2 photoreceptors and membrane-bound kinases are members of this AGC kinase family, which suggests a possible link between the chloroplast movement and the photosynthesis with the drought-tolerance [85]. GO analysis of these SRPs (significantly regulated phosphoproteins; Supplementary Table S6a) found that their cellular functions are also enriched in chloroplast, photosynthesis, cytoskeleton, and membrane systems, further suggesting a role for the increase of protein phosphorylation in chloroplast movement and photosynthesis during development of drought tolerance. In contrast, the S/T-P and the S/T-D phosphosite motif is probably the docking target of CDPKs (calcium-dependent protein kinases) and MAPKs (mitogen-activated protein kinases), respectively. The CDPKs, presenting in many phylum of plant [86], constitute a large and conserved kinase family, and they are commonly involved in plant hormone response and stress signaling pathways [87,88]. In this study, we found that out of nine kinase families identified from this phosphoproteomics study (Figure 2), only three members of CAMK (calcium dependent protein kinase) kinase family, GmSRK2I, GmCIPK25, and GmAKINβ1, constitute a cascade of phosphor-relay pathway in regulation of drought stress response (Figure 6), phosphorylating many downstream phosphoproteins including GmEIN2, a key stress hormone ethylene’s signaling regulator (Figure 6).

The MAPKs cascades, on the other hand, are well-known to be involved largely in the drought signaling pathways, and they normally trigger the expression of down-stream drought-related genes [89,90]. The bioinformatics analysis of those significantly down-regulated phosphoproteins revealed a large number of both cytosolic and membrane-bound kinases (Figure 4B), the downregulation of these kinases’ phosphorylation may influence the phosphorylation level of both S/T-P and the S/T-D phosphosites (Figure 4A; Supplementary Table S6a). These kinases of severely modified phosphorylation may participate in regulation of drought signaling and drought tolerance development in soybean [91]. Especially, our bioinformatics study found that a GmMKKK phosphorylates a GmPP2C phosphatase that has been implicated in plant stress response.

Further analysis of the significantly regulated phosphopeptide groups reveals some phosphorylated proteins found for the first time to be involved in drought treatment and considered to be novel in drought tolerance response. For example, BLUS1 (blue light signaling 1) gene encodes a putative Ser/Thr protein kinase, a member of STE Ser/Thr protein kinase family, and functions as a phototropin substrate and a primary regulator of stomatal control to enhance photosynthetic CO_2_ assimilation under the natural light conditions [92,93]. BLUS1 protein may work in concern with CIPK23 (CBL-interacting protein kinase 23) kinase to promote stomatal opening through activation of K^+^ in channels [94]. Under drought treatment, the higher phosphorylation level of BLUS1 in drought-tolerant cultivar might either come from the kinase activity of CIPK23 kinase or from PHOT1 (phototropin 1) and PHOT2 (phototropin 2) photoreceptors as it has been reported that the abundance of proteins, SnRK4 (Snf1-related serine/threonine-protein kinase), PHOT1, and PHOT2, are all associated with the stomatal conductance and the improvement of the drought stress tolerance [95]. The higher phosphorylation level of BLUS1 in drought-tolerant cultivar might contribute to the better performance in the photosynthetic CO_2_ assimilation in this cultivar under drought treatment. Our study revealed that the GmCIPK25 also participates in the stress response and probably is specific to drought stress response in soybean.

For another example, *Arabidopsis PUX5* gene encodes a serine/threonine protein phosphatase 2A 55 kDa regulatory subunit B prime Gamma. This phosphatase is involved in the Golgi organization, autophagosome assembly, membrane fusion, nuclear envelope reassembly, proteasome-mediated ubiquitin-dependent protein catabolic process and so on. Most importantly, it interacts with S6K1 kinase, which is involved in translational up-regulation of ribosomal proteins. The activity of S6K1 is affected by the osmotic stress. Plants overexpressing S6k1 are hypersensitive to osmotic stress [96]. It is therefore likely that the highly phosphorylated GmPUX5 phosphatase may dephosphorylate the GmS6K1 protein to confer plant cell resistant to the water loss and osmotic changes during drought treatment, which might lead to the better field performance under drought condition.

Furthermore, *Arabidopsis BSU1* (BRI1 SUPPRESSOR 1) gene family has four members, *BSU1*, *BSL1* (*BSU1-LIKE 1*), *BSL2*, and *BSL3*, which enhance the BR (brassinosteroid) signaling through hetero- and homo-oligomerization [97]. BSL1 (BSU1-LIKE 1), a putative phosphatase, is not only implicated in growth-promoting BR signaling, its phosphatase activity may contribute to the recognition of the *P. infestans* AVR2 effector by the plant host NB-LRR protein R2 [98] as well. This putative phosphatase has also been documented to interact with RLCK VII kinase during pattern-triggered immune signaling [99]. Especially, the abundance of BSL1 was enhanced under PEG treatment [100]. Taken together, the fact that the drought-tolerant cultivar increases GmBSU1 phosphorylation level under drought treatment allows us to speculate that GmBSL1 may participate in the development of drought tolerance in soybean plant.

During the network analysis (Figure 5), it was found that a few nodes have more edges connected than the average of the entire network (average = 2.8), indicating these network hubs may play key roles in regulating the drought tolerance in soybean. For example, the *GmSRK2I* or *GmSnRK2I* gene has the highest number of connections in the whole functional network. The *GmSRK2I* gene is a member of *GmSnRK2* gene family, and it is a key regulator in plant biotic and abiotic stress response, including ABA, salt, and drought responses [101,102,103]. Under drought stress, it was found that the *Arabidopsis SnRK2.8* gene is positively correlated with the drought tolerance of *Arabidopsis*. The overexpression of *AtSRK2.8-GFP* fusion gene was reported to increase the drought tolerance of *Arabidopsis* by up-regulation of the drought-responsive genes [103]. Besides, the activity of AtSnRK2 kinase can be increased by the loop auto-phosphorylation [104]. These findings were consistent with our quantitative phosphoproteomic results as the phosphorylation site in GmSnRK2I protein was also drought-tolerant cultivar up-regulated (Supplementary Table S6a).

In addition, the putative transcription factor GmGRP2 protein (glycine-rich protein 2-like) has the highest number of connected edges (9 of them) in the transcription factor cluster. GmGRP2 contains a well conserved S1-like cold-shock domain in plants, and it was found to be up-regulated by a temperature decrease [105,106]. In *Arabidopsis*, there were four cold shock domain proteins, AtCSP1, AtCSP2, AtCSP3, and AtCSP4. The earlier investigation has suggested that these cold shock domain-containing proteins, AtCSP1, AtCSP2 and AtCSP3, are involved in the drought tolerance of *Arabidopsis* [106,107,108]. According to well documented studies, these AtCSP proteins are presumably chaperones involved in the binding and the unwinding of both RNA and DNA molecules, thus probably participating in the regulation of the gene expression and further affecting the seeds’ germination under dehydration condition [107,109]. These results strongly suggest that the drought upregulated GmGRP2 phosphorylation may also participate in regulation of the drought-tolerant response in soybean.

Among all of the drought-tolerant cultivar significantly regulated phosphoproteins, GmLTI65, a low-temperature-induced protein 65, is the most interesting phosphoprotein. The *Arabidopsis* homolog (AT5G52300) of this gene encodes a well-known ABA (abscisic acid)-dependent, senescence, cold, salt, sterols accumulation, and drought-induced protein RD29B (responsive to desiccation 29B; [110,111]). Numerous transgenic soybean plants over-expressing transcription factors that up-regulate RD29 gene expression conferred drought tolerance phenotype to these soybean transgenics [112,113]. In this study, we found that LTI65 protein has a connectivity of 6, which ranks at the top 15% of the total nodes. In addition, the GmLTI65 phosphoprotein is classified into six biological function clusters, i.e., water deprivation, response to abscisic acid, inorganic substance, osmotic stress, oxygen-containing component and hormone (Figure 5), indicating the essential role of LTI65 phosphoprotein in the abiotic response tolerance [81,114,115]. Especially, the GmLTI65 protein has a total of 12 drought-responsive phosphosites, S123, S183, S231, S333, S344, S400, S423, S442, S446, S494, S513, and S529 cross the entire protein (Figure 4D) and is considered as one of the OPPs (overly phosphorylated proteins) found from this experimental dataset (Figure 2G–I; Supplementary Table S2e). By the GO analysis of those OPPs (Figures S5 and S6), it is speculated that this cytosolic OPP GmLTI65 may have a function in RNA-binding out of 10 categories of molecular functions for OPPs (Figure S5). Our finding is consistent with the postulation that the overall RBPs (RNA-binding proteins) abundance alteration at a proteomic scale is associated with the plant adaptation to drought stress and it may play role in the RNA metabolism [116].

Comparative proteomics has been applied before into the study of both drought-tolerant and drought-sensitive soybean seedlings under drought treatment [117]. Among these drought-regulated differentially abundant proteins (DAPs), nearly one-third of proteins were categorized into energy metabolism and photosynthetic functions, another one third into the defense response-related proteins and finally 25.2% of the proteins into metabolism-related ones. Especially, the tolerant soybean cultivar showed a higher capacity in the reactive oxygen species-scavenging and in maintaining the energy supply as compared to that of the sensitive cultivar. Similarly, when the similar differential and quantitative proteomics approach was adopted to investigate the two contrasting cultivars in drought tolerance from both monocot plants, rice [118], wheat [119], maize [120], dicot plants, potatoes [121], pea [122], Brassica [123], chickpea [124], tobacco [34], and tea plant [125], it was found that these drought-related DAPs in general play a role in regulating carbohydrate, glutathione, amino acids, sucrose and nitrogen metabolism, redox homeostasis and ROS-scavenging, protein synthesis and processing (or upregulation of ClpD1 protease), defense and stress-response processes, photosynthesis, cell wall biogenesis and degradation, cytoskeleton metabolism and energy production. However, in this quantitative phosphoproteomics study, we found that the drought-tolerant cultivar SRPs (significantly regulated phosphoproteins) are mostly related to water transport and deprivation, methionine metabolic process, photosynthesis/light reaction, response to cadmium ion, osmotic stress and ABA (hormone) under drought treatment (Figure 4D and Figure 5). The increase of phosphorylation level on several water channel proteins (Figure 5) strongly suggested that this drought-tolerant soybean cultivar may have developed more efficient water transport mechanism (Figure 5) to mobilize water from the root system to the water-deprived aerial part of soybean seedlings in response to the drought treatment. This speculation is supported by the earlier findings that the stress hormone ethylene upregulated phosphorylation of PIP2;1 plays a key role in regulating water influxes into plant cells [126].

## Data Availability

The mass spectrometry data have been deposited to the ProteomeXchange [127] Consortium via PRIDE [128] with dataset identifier PXD022794. Username: reviewer_pxd022794@ebi.ac.uk, Password: s2ViBvxf.

## Supplementary Materials

The following are available online at https://www.mdpi.com/article/10.3390/biom11081218/s1, Supplementary Table S1: Yield of protein and peptide preparation. Supplementary Table S2a: A table contains all PSMs (Peptide Spectrum Matches) with *q*-value ≤ 0.01. Supplementary Table S2b: A table contains all redundant PSMs with *q*-value ≤ 0.01, Mascot delta score ≥ 10. Supplementary Table S2c: A table contains all non-redundant L and H phosphopeptides with *q*-value ≤ 0.01, Mascot delta score ≥10. Supplementary Table S2d: A table contains all non-redundant and label-independent phosphopeptides. Supplementary Table S2e: A table contains all phosphosites identified from the repeatable phosphopeptides. Supplementary Table S2f: A table contains all phosphoprotein groups. Supplementary Table S2g: A table contains all leading proteins. Supplementary Table S2h. A table contains all unique proteins. Supplementary Table S2i: A table contains a combination of unique protein from this study and EPSD database. Supplementary Table S3a: Classification of kinase proteins. Supplementary Table S3b: GPS results of prediction of kinase families among all the phosphosites. Supplementary Table S3c: Mapping of the phosphosite with kinase proteins. Supplementary Table S3d: Mapping of the phosphosite with kinase families annotated in STRING or BioGRID database. Supplementary Table S3e: Mapping of the phosphosite with kinase proteins annotated in STRING or BioGRID database. Supplementary Table S3f: Motifs enriched from each kinase family. Supplementary Table S4a: Classification of kinase proteins (EPSD database). Supplementary Table S4b: GPS results of prediction of kinase families among all the phosphosites (EPSD database). Supplementary Table S4c: Mapping of the phosphosite with kinase proteins (EPSD database). Supplementary Table S4d: Mapping of the phosphosite with kinase families annotated in STRING or BioGRID database (EPSD database). Supplementary Table S4e: Mapping of the phosphosite with kinase proteins annotated in STRING or BioGRID database (EPSD database). Supplementary Table S4f: Motifs enriched from each kinase family (EPSD database). Supplementary Table S5a: A table contains all non-redundant UPA with *q*-value ≤ 0.01 and Mascot delta score ≥ 10 and repeat at least twice. Supplementary Table S5b: A table containing the quantification results of all quantifiable UPAs. Supplementary Table S6a: The drought tolerant significantly regulated phosphoproteins. Supplementary Table S6b: The STRING annotated interactions between the significantly regulated phosphoprotein. Supplementary Table S7: Primers designed for RT-qPCR. Supplementary Table S8: Overlap between the significantly regulated phosphopeptide groups and kinase-substrate map. Supplementary Figure S1: The drought treatment. Supplementary Figure S2: The quantitative phosphoproteomic workflow. Supplementary Figure S3: Mapping of the proteomic data processing and figure generation. Supplementary Figure S4: Computational analysis of LC–MS/MS data of phosphopeptides. Supplementary Figure S5: Molecular function analysis of overly post-translationally modified protein (OPP). Supplementary Figure S6: Biological process and cellular component analysis of overly post-translationally modified protein (OPP). Supplementary Figure S7: The mapping of the relationship in between kinase family and the kinase docking site motif. Supplementary Figure S8: Interactome of kinase and substrates with GPS predication and STRING/BioGRID filtering. Supplementary Figure S9: The extracted ion chromatograms (XIC) of the significantly regulated phosphopeptides. Supplementary Figure S10: The RT-qPCR validation of representatives of the drought-tolerant cultivar significantly regulated phosphoprotein groups.
