# Supplementary material for: Isotopically Dimethyl Labeling-Based Quantitative Proteomic Analysis of Phosphoproteomes of Soybean Cultivars
Source: Biomolecules. 2021 Aug 16;11(8):1218. doi: 10.3390/biom11081218 (PMC8393417; doi:10.3390/biom11081218)
Supplement: Supplementary file 1 [file biomolecules-11-01218-s001.zip › Supplementary Figures.pdf]

Supplemental Figures

Figure S1

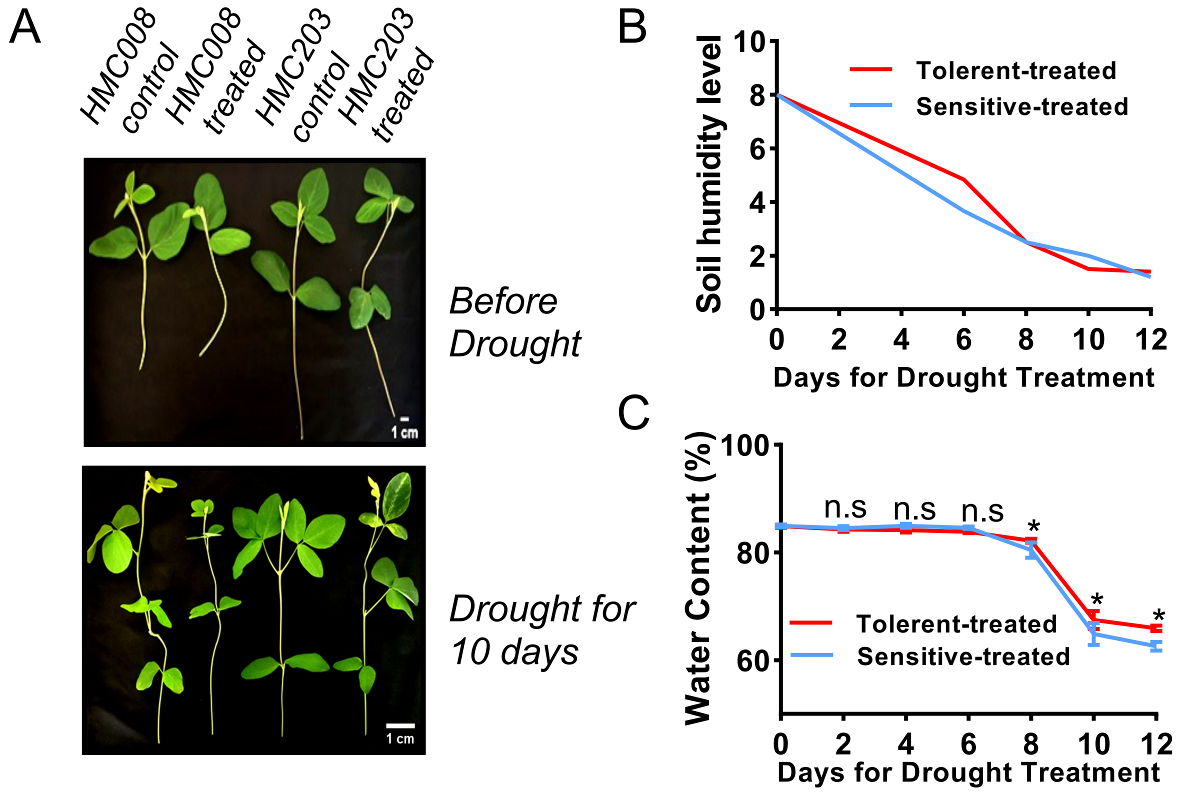

Figure S1. **The drought treatment**

(A) The representative photos of the control and the drought-treated soybean plants of both drought-tolerant (HMC 203) and -sensitive (HMC 008) cultivar.

(B) The soil humidity level during the 12-day period of drought treatment.

(C) The change of the water content percentage of drought-tolerant and -sensitive lines. The error bar stands for the SEM. Unpair, one-tail and unequal variance student's *t*-test was performed. The n.s stands for no significant difference while * stands for $p < 0.05$.

Figure S2

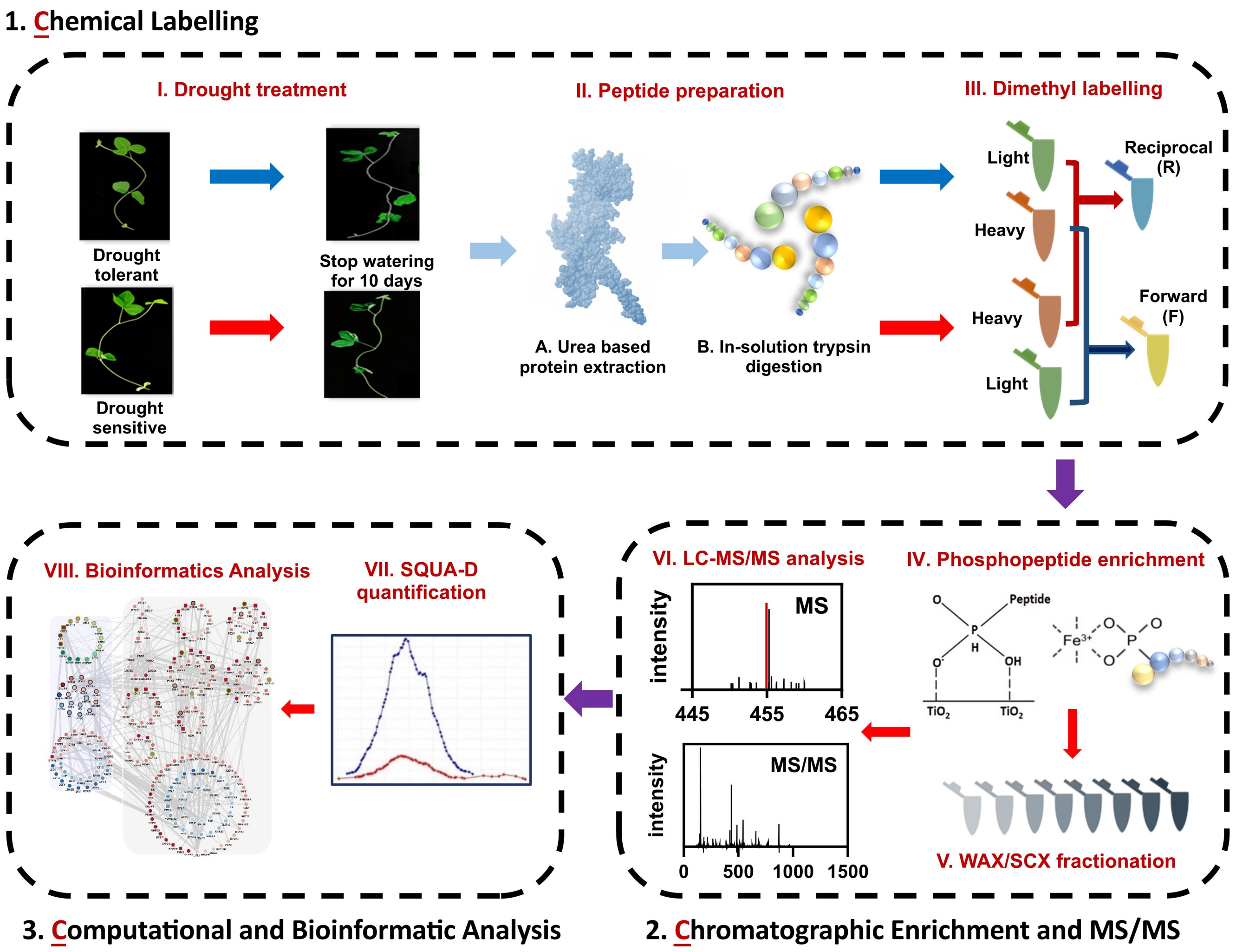
1. Chemical Labelling
I. Drought treatment
Drought tolerant
Drought sensitive
Stop watering for 10 days
II. Peptide preparation
A. Urea based protein extraction
B. In-solution trypsin digestion
III. Dimethyl labelling
Light
Heavy
Heavy
Light
Reciprocal (R)
Forward (F)
VIII. Bioinformatics Analysis
VII. SQUA-D quantification
VI. LC-MS/MS analysis
MS
intensity
445
455
465
MS/MS
0
500
1000
1500
IV. Phosphopeptide enrichment
Peptide
TiO_2
TiO_2
Fe^{3+}
V. WAX/SCX fractionation
3. Computational and Bioinformatic Analysis
2. Chromatographic Enrichment and MS/MS

Figure S2. The quantitative phosphoproteomic workflow.

The multiple steps of this quantitative PTM proteomics has been summarized into the **3C** proteomic workflow, which stands for (1) the c̲hemical labeling, (2) c̲hromatographic enrichment and MS/MS analysis and (3) c̲omputational and bioinformatic analysis. In 1st C step, two soybean cultivars, the drought-tolerant and the drought-sensitive, were treated under the drought condition for 10 days simultaneously (I). The total cellular protein was extracted from both cultivars (**Supplementary Table S0**) and subjected to trypsin digestion (II) . The isotopic dimethyl labeling was performed on two pairs of peptides using both light formaldehyde ($^{12}CH_2O$) and heavy formaldehyde chemical ($^{13}CD_2O$) to generate both forward (F) and reciprocal (R) mixings (III). In the chromatographic enrichment and MS/MS analysis, the phosphopeptides were enriched tandemly using both TiO_2 and Fe^{3+}-NTA IMAC resins (IV), followed by both WAX and SCX column fractionation (V). The fractionated phosphopeptide samples were analyzed consequently using LC-MS/MS (VI). Computational analysis of MS raw data was performed using Mascot and SQUA-D for PSM identification and quantitation, respectively. The identification and quantification data were subjected to bioinformatics analysis (VII) thereafter.

Figure S3

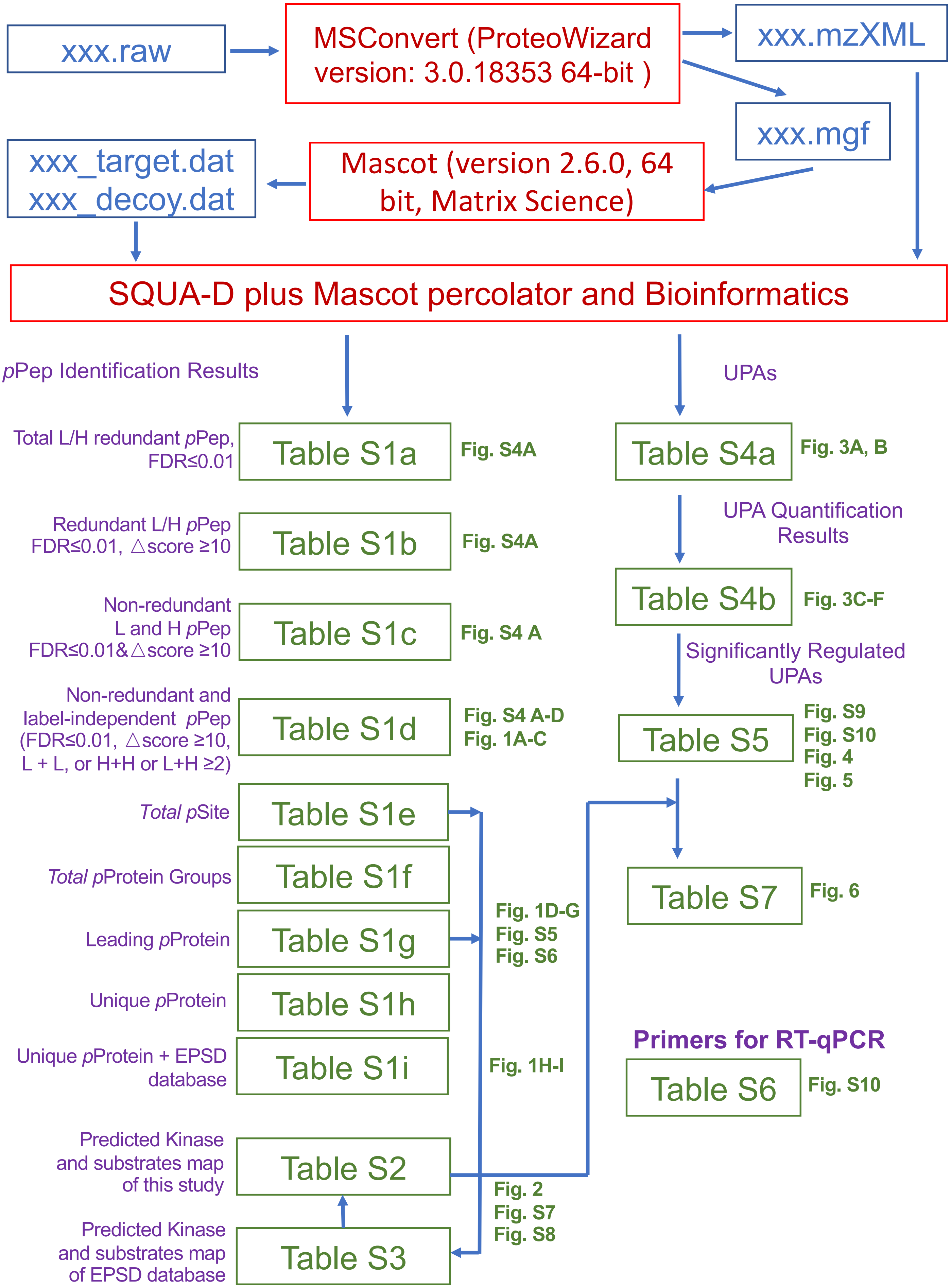

xxx.raw
MSConvert (ProteoWizard version: 3.0.18353 64-bit)
xxx.mzXML
xxx.mgf
xxx_target.dat
xxx_decoy.dat
Mascot (version 2.6.0, 64 bit, Matrix Science)
SQUA-D plus Mascot percolator and Bioinformatics
pPep Identification Results
UPAs
Total L/H redundant pPep, FDR≤0.01
Table S1a
Fig. S4A
Table S4a
Fig. 3A, B
UPA Quantification Results
Redundant L/H pPep FDR≤0.01, △score ≥10
Table S1b
Fig. S4A
Table S4b
Fig. 3C-F
Non-redundant L and H pPep FDR≤0.01&△score ≥10
Table S1c
Fig. S4 A
Significantly Regulated UPAs
Non-redundant and label-independent pPep (FDR≤0.01, △score ≥10, L + L, or H+H or L+H ≥2)
Table S1d
Fig. S4 A-D
Fig. 1A-C
Table S5
Fig. S9
Fig. S10
Fig. 4
Fig. 5
Total pSite
Table S1e
Total pProtein Groups
Table S1f
Table S7
Fig. 6
Leading pProtein
Table S1g
Fig. 1D-G
Fig. S5
Fig. S6
Unique pProtein
Table S1h
Primers for RT-qPCR
Unique pProtein + EPSD database
Table S1i
Fig. 1H-I
Table S6
Fig. S10
Predicted Kinase and substrates map of this study
Table S2
Fig. 2
Fig. S7
Fig. S8
Predicted Kinase and substrates map of EPSD database
Table S3

Figure S3. Mapping of the proteomic data processing and figure generation.

The raw data was converted to both mzXML and mgf files through MSConverter (version 3.0.18353). The mgf files were searched against the Arabidopsis target and decoy TAIR10 database using the Mascot (version 2.6.0). The output of the Mascot are pairs of target and decoy Dat. files. The paired target and decoy files with related mzXML files were used as the input of both Percolator (version 3.02) and SQUA-D (Stable isotope-based Quantitation-Dimethyl labeling, Version 2.0) to obtain both identification and quantification results. The data processing and figure generation procedure is depicted in the flowchart: Supplementary table S1a contains all the ambiguous, redundant, non-repeatable and label-dependent phosphopeptides of FDR <0.01. Supplementary Table S1b contains **unambiguous**, redundant, non-repeatable and label-dependent phosphopeptides of both FDR <0.01 and delta score ≥10. Supplementary table S1c contains **unambiguous, non-redundant**, non-repeatable and label-dependent phosphopeptides of both FDR <0.01 and delta score ≥10. Supplementary table S1d contains **unambiguous, non-redundant, repeatable and label-independent** phosphopeptides. Supplementary table S1e contains all the phosphosites of leading proteins. Supplementary table S1f contains all the phosphoprotein groups. Supplementary table S1g contains all leading proteins. Supplementary table S1h contains all unique proteins. Supplementary table S1i contains a combination of unique protein from this study and the EPSD database. Supplementary S2a contains the kinase classification results. Supplementary S2b contains the GPS prediction of kinase and phosphosite relationship. Supplementary S2c contains kinase and phosphosites relationship. Supplementary S2d contains kinase and phosphosites relationship filtered by STRING and BioGrid. Supplementary S2e contains motifs enrichment within each kinase families using both Motif-X and Motif-ALL software. Supplementary S3 contains the similar bioinformatic analysis results of phosphosites deposited in the EPSD database. Supplementary table S4a contains all the quantifiable UPAs while the supplementary table S4b contains the UPA quantification results. Supplementary table S5 contains significantly regulated UPAs. Supplementary S6 contains information of the primers used in RT-qPCR study. Supplementary S7 contains the overlapping results between the significantly regulated phosphopeptide groups and kinase-substrate prediction results.

Figure S4A was generated using Supplementary table S1a-d. Figure S4B, Figure 1A-C were generated using Supplementary table S1d. Figure 1D-G and Figure S5-S6 were generated using Supplementary table S1e-h; Figure 1H-I was generated using Supplementary table S1i. Figure 2 and Figure S7-S8 were generated using Supplementary table S2 and S3; Figure 3 was generated using Supplementary table S4. Figure 4-5 and Figure S9 were generated using Supplementary table S4 and S5. Figure S10 was generated using Supplementary table S5 and S6. Figure 6 was generated using Supplementary table S7.

Figure S4

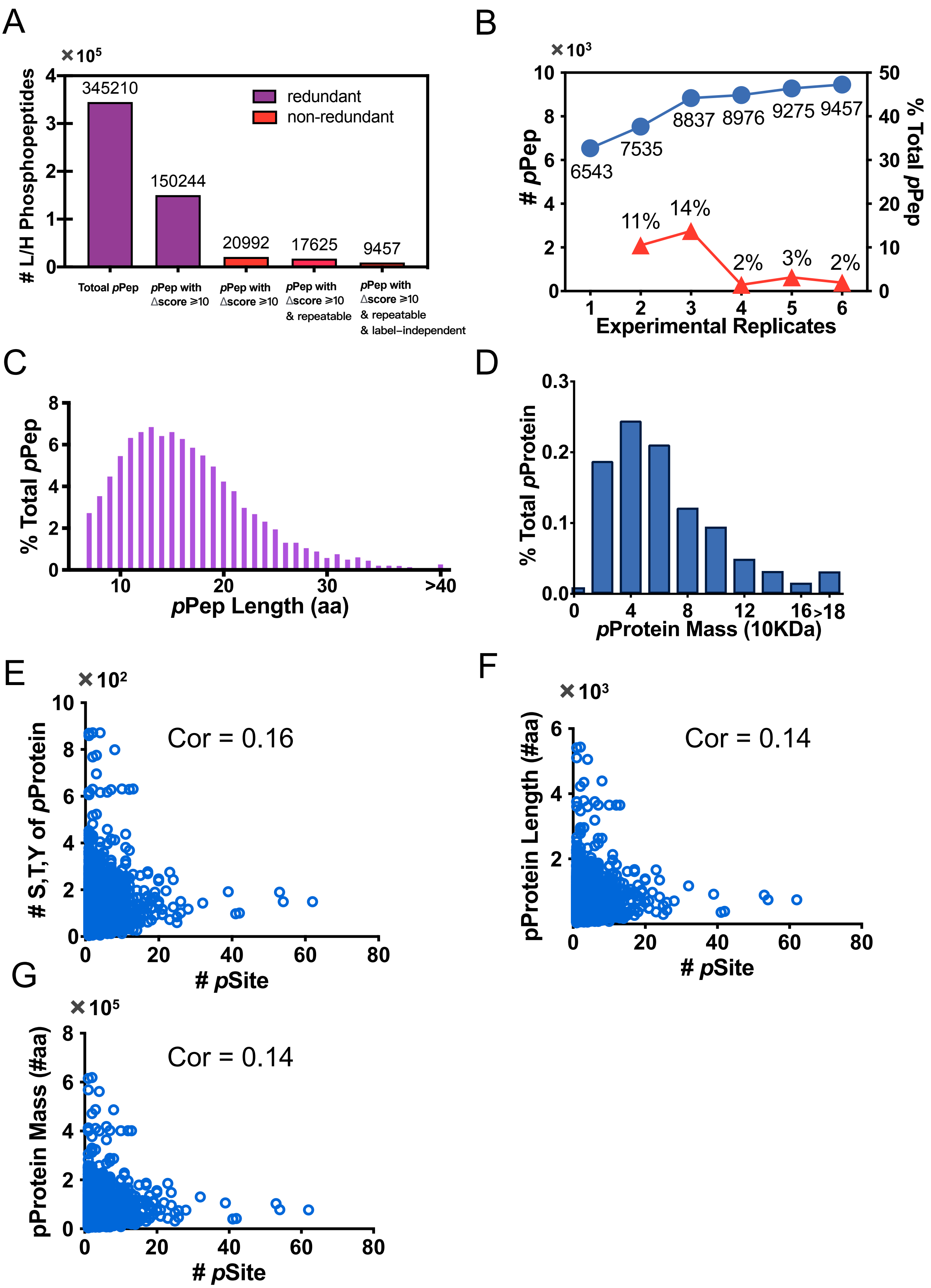

A
×10^5
L/H Phosphopeptides
345210
150244
20992
17625
9457
redundant
non-redundant
Totoal pPep
pPep with Δscore ⩾10
pPep with Δscore ⩾10
pPep with Δscore ⩾10 & repeatable
pPep with Δscore ⩾10 & repeatable & label-independent
B
×10^3
pPep
% Total pPep
6543
7535
8837
8976
9275
9457
11%
14%
2%
3%
2%
Experimental Replicates
C
% Total pPep
pPep Length (aa)
>40
D
% Total pProtein
pProtein Mass (10KDa)
16>18
E
×10^2
# S,T,Y of pProtein
Cor = 0.16
# pSite
F
×10^3
pProtein Length (#aa)
Cor = 0.14
# pSite
G
×10^5
pProtein Mass (#aa)
Cor = 0.14
# pSite

Figure S4. Computational analysis of LC-MS/MS data of phosphopeptides

(A) The bar represents the number (345,210) of ambiguous, redundant, non-repeatable and label-dependent phosphopeptides of a FDR <0.01 (**Supplementary Table S1a**), the number (150,244) of **unambiguous**, redundant, non-repeatable and label-dependent phosphopeptides of both FDR <0.01 and delta score ≥10 (**Supplementary Table S1b**), the number (20,992) of **unambiguous**, **non-redundant**, **non-repeatable** and label-dependent phosphopeptides of both FDR <0.01 and delta score ≥10 (**Supplementary Table S1c**), the number (17,625) of **unambiguous**, **non-redundant**, **repeatable** and label-dependent phosphopeptides of FDR <0.01, delta score ≥10 and the isotope-coding type of L + H ≥ 2, L + L ≥ 2 or H + H ≥ 2 (**Supplementary Table S1c**) and the number (9457) of **unambiguous**, **non-redundant**, **repeatable** and **label-independent** phosphopeptides (**Supplementary Table S1d**) of both FDR < 0.01 and delta score ≥ 10, respectively. This group of phosphopeptides are used for all of the rest phosphoproteomic analysis.

(B) The blue curve represents the accumulation of phosphopeptides while the red curve represents the cumulating contribution (or a fraction of the total) over 6 experimental replicates of all 3 biological replicates. All phosphopeptides used for this bioinformatic analysis and figure-making come from the repeatable ones (9457) in the **Supplementary Table S1d** as indicated in the **Fig. S4.** *p*Pep stands for phosphopeptide(s).

(C) The distribution of the identified phosphopeptides (9457) over their primary lengths (**Supplementary Table S1d**).

(D) The distribution of the molecular weight of identified leading phosphoproteins (3889). The *p*Protein stands for phosphoprotein(s) (**Supplementary Table S1g)**

(E) The correlation in between the number of amino acid (S, serine; T, threonine; Y, tyrosine) with a specific number of phosphosites of phosphoproteins. The Cor (0.16) represents the Pearson correlation number. The data used in this figure are from the unique phosphoprotein data of this study (1808) and those of the EPSD database (3747, **Supplementary Table S1i**).

(F) The correlation in between the primary aa sequence length and the specific number of phosphosite of a phosphoprotein. The Cor (0.14) represents the Pearson correlation number. The data used in this figure are from the unique phosphoprotein data of this study (1808) and those of the EPSD database (3747, **Supplementary Table S1i**).

(G) The correlation in between the molecular weight (monoisotopic mass) and the specific number of phosphosite of a phosphoprotein. The Cor (0.14) represents the Pearson correlation number. The data used in this figure are from the unique phosphoprotein data of this study (1808) and those of the EPSD database (3747, **Supplementary Table S1i**).

Figure S5

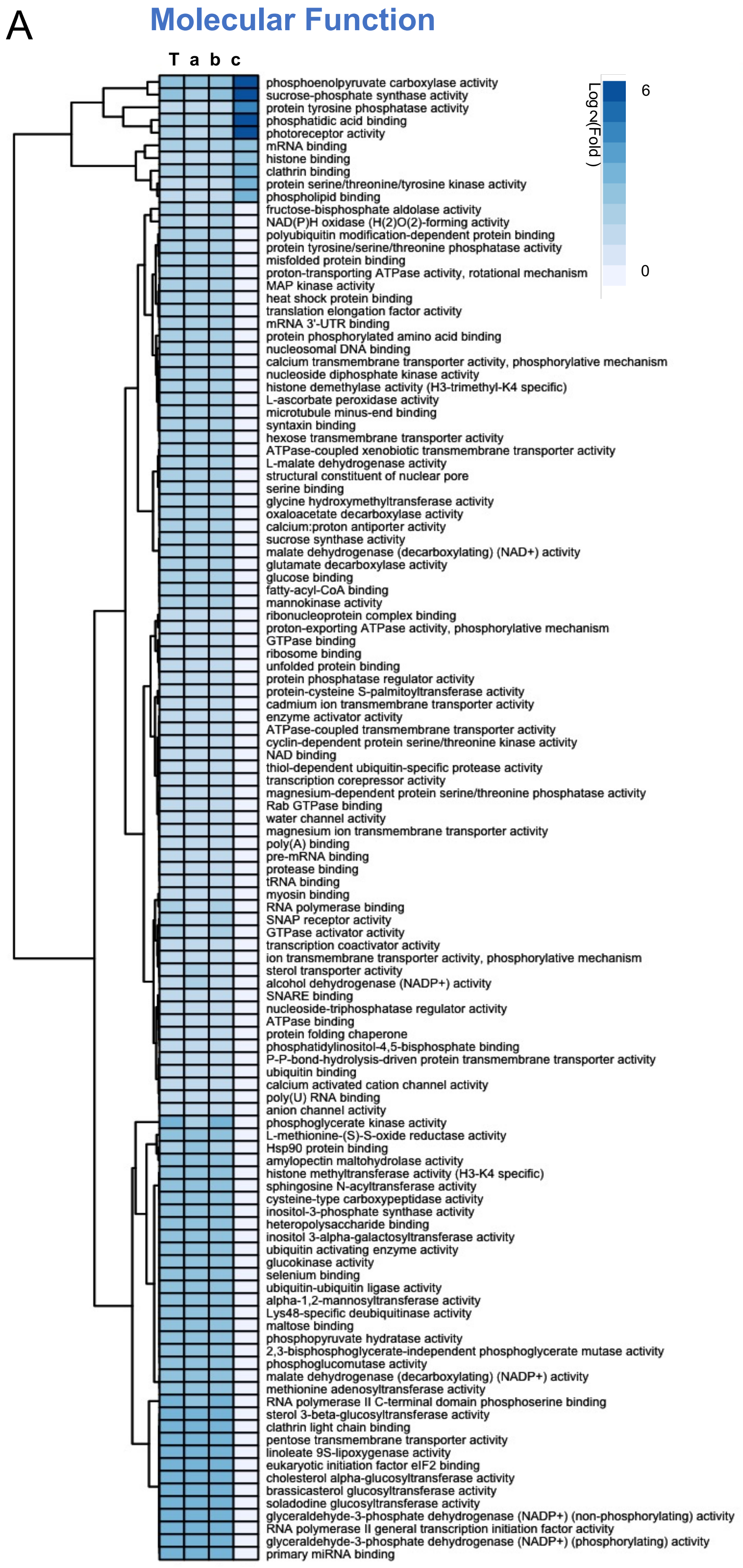
A
Molecular Function
T a b c
Log2(Fold)
6
0
phosphoenolpyruvate carboxylase activity
sucrose-phosphate synthase activity
protein tyrosine phosphatase activity
phosphatidic acid binding
photoreceptor activity
mRNA binding
histone binding
clathrin binding
protein serine/threonine/tyrosine kinase activity
phospholipid binding
fructose-bisphosphate aldolase activity
NAD(P)H oxidase (H(2)O(2)-forming activity
polyubiquitin modification-dependent protein binding
protein tyrosine/serine/threonine phosphatase activity
misfolded protein binding
proton-transporting ATPase activity, rotational mechanism
MAP kinase activity
heat shock protein binding
translation elongation factor activity
mRNA 3'-UTR binding
protein phosphorylated amino acid binding
nucleosomal DNA binding
calcium transmembrane transporter activity, phosphorylative mechanism
nucleoside diphosphate kinase activity
histone demethylase activity (H3-trimethyl-K4 specific)
L-ascorbate peroxidase activity
microtubule minus-end binding
syntaxin binding
hexose transmembrane transporter activity
ATPase-coupled xenobiotic transmembrane transporter activity
L-malate dehydrogenase activity
structural constituent of nuclear pore
serine binding
glycine hydroxymethyltransferase activity
oxaloacetate decarboxylase activity
calcium:proton antiporter activity
sucrose synthase activity
malate dehydrogenase (decarboxylating) (NAD+) activity
glutamate decarboxylase activity
glucose binding
fatty-acyl-CoA binding
mannokinase activity
ribonucleoprotein complex binding
proton-exporting ATPase activity, phosphorylative mechanism
GTPase binding
ribosome binding
unfolded protein binding
protein phosphatase regulator activity
protein-cysteine S-palmitoyltransferase activity
cadmium ion transmembrane transporter activity
enzyme activator activity
ATPase-coupled transmembrane transporter activity
cyclin-dependent protein serine/threonine kinase activity
NAD binding
thiol-dependent ubiquitin-specific protease activity
transcription corepressor activity
magnesium-dependent protein serine/threonine phosphatase activity
Rab GTPase binding
water channel activity
magnesium ion transmembrane transporter activity
poly(A) binding
pre-mRNA binding
protease binding
tRNA binding
myosin binding
RNA polymerase binding
SNAP receptor activity
GTPase activator activity
transcription coactivator activity
ion transmembrane transporter activity, phosphorylative mechanism
sterol transporter activity
alcohol dehydrogenase (NADP+) activity
SNARE binding
nucleoside-triphosphatase regulator activity
ATPase binding
protein folding chaperone
phosphatidylinositol-4,5-bisphosphate binding
P-P-bond-hydrolysis-driven protein transmembrane transporter activity
ubiquitin binding
calcium activated cation channel activity
poly(U) RNA binding
anion channel activity
phosphoglycerate kinase activity
L-methionine-(S)-S-oxide reductase activity
Hsp90 protein binding
amylopectin maltohydrolase activity
histone methyltransferase activity (H3-K4 specific)
sphingosine N-acyltransferase activity
cysteine-type carboxypeptidase activity
inositol-3-phosphate synthase activity
heteropolysaccharide binding
inositol 3-alpha-galactosyltransferase activity
ubiquitin activating enzyme activity
glucokinase activity
selenium binding
ubiquitin-ubiquitin ligase activity
alpha-1,2-mannosyltransferase activity
Lys48-specific deubiquitinase activity
maltose binding
phosphopyruvate hydratase activity
2,3-bisphosphoglycerate-independent phosphoglycerate mutase activity
phosphoglucomutase activity
malate dehydrogenase (decarboxylating) (NADP+) activity
methionine adenosyltransferase activity
RNA polymerase II C-terminal domain phosphoserine binding
sterol 3-beta-glucosyltransferase activity
clathrin light chain binding
pentose transmembrane transporter activity
linoleate 9S-lipoxygenase activity
eukaryotic initiation factor eIF2 binding
cholesterol alpha-glucosyltransferase activity
brassicasterol glucosyltransferase activity
soladodine glucosyltransferase activity
glyceraldehyde-3-phosphate dehydrogenase (NADP+) (non-phosphorylating) activity
RNA polymerase II general transcription initiation factor activity
glyceraldehyde-3-phosphate dehydrogenase (NADP+) (phosphorylating) activity
primary miRNA binding

Figure S5

Molecular function enrichment analysis of Arabidopsis ortholog of total phosphoproteins (T) and phosphoproteins with 1 to 4 phosphosites (a), 5 to 10 phosphostes (b) and larger than 10 phosphosites (c) listed in **Supplementary Table S1e**. The blue color palette of cell represents the logarithm of the fold enrichment.

Figure S6

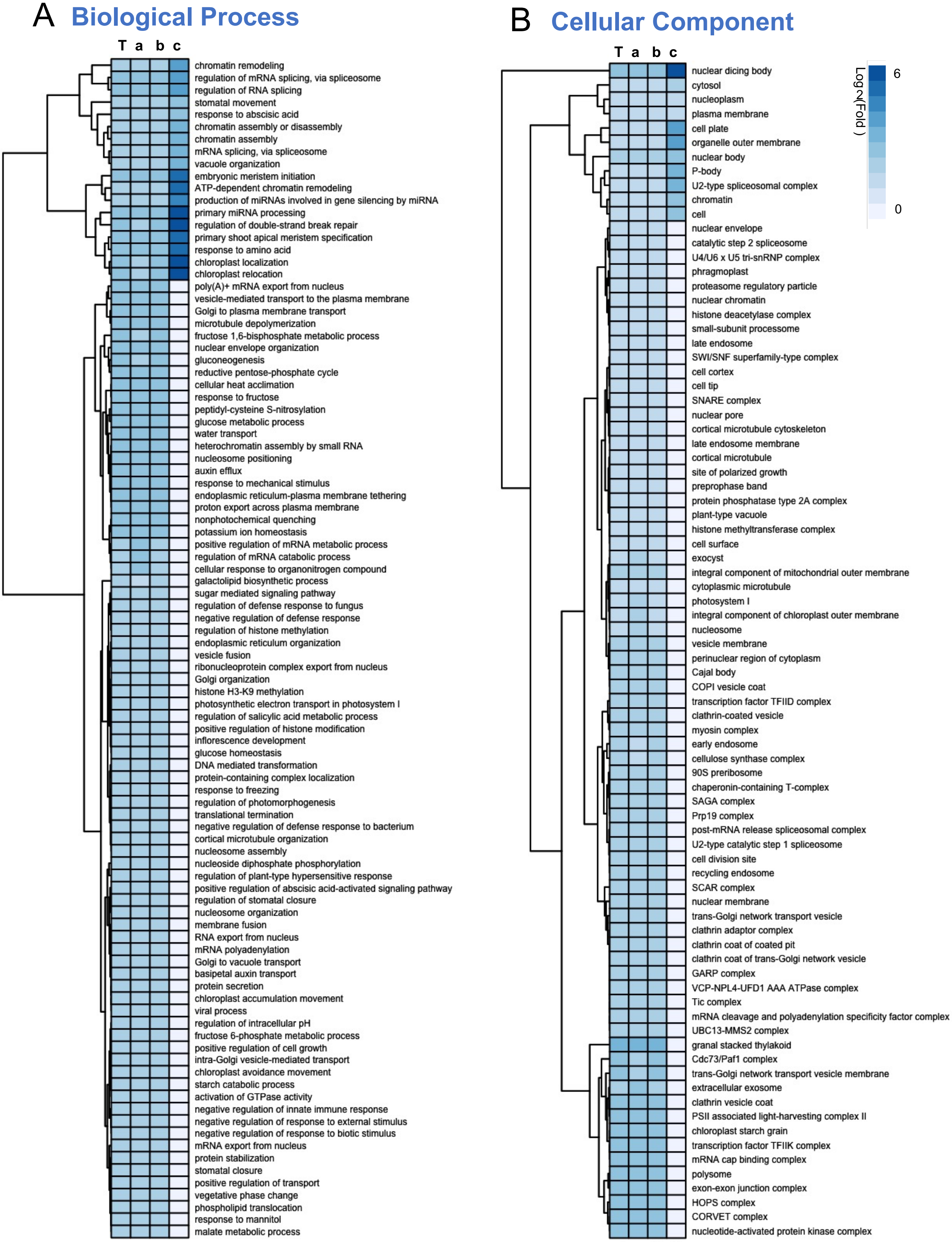
A
Biological Process
T a b c
chromatin remodeling
regulation of mRNA splicing, via spliceosome
regulation of RNA splicing
stomatal movement
response to abscisic acid
chromatin assembly or disassembly
chromatin assembly
mRNA splicing, via spliceosome
vacuole organization
embryonic meristem initiation
ATP-dependent chromatin remodeling
production of miRNAs involved in gene silencing by miRNA
primary miRNA processing
regulation of double-strand break repair
primary shoot apical meristem specification
response to amino acid
chloroplast localization
chloroplast relocation
poly(A)+ mRNA export from nucleus
vesicle-mediated transport to the plasma membrane
Golgi to plasma membrane transport
microtubule depolymerization
fructose 1,6-bisphosphate metabolic process
nuclear envelope organization
gluconeogenesis
reductive pentose-phosphate cycle
cellular heat acclimation
response to fructose
peptidyl-cysteine S-nitrosylation
glucose metabolic process
water transport
heterochromatin assembly by small RNA
nucleosome positioning
auxin efflux
response to mechanical stimulus
endoplasmic reticulum-plasma membrane tethering
proton export across plasma membrane
nonphotochemical quenching
potassium ion homeostasis
positive regulation of mRNA metabolic process
regulation of mRNA catabolic process
cellular response to organonitrogen compound
galactolipid biosynthetic process
sugar mediated signaling pathway
regulation of defense response to fungus
negative regulation of defense response
regulation of histone methylation
endoplasmic reticulum organization
vesicle fusion
ribonucleoprotein complex export from nucleus
Golgi organization
histone H3-K9 methylation
photosynthetic electron transport in photosystem I
regulation of salicylic acid metabolic process
positive regulation of histone modification
inflorescence development
glucose homeostasis
DNA mediated transformation
protein-containing complex localization
response to freezing
regulation of photomorphogenesis
translational termination
negative regulation of defense response to bacterium
cortical microtubule organization
nucleosome assembly
nucleoside diphosphate phosphorylation
regulation of plant-type hypersensitive response
positive regulation of abscisic acid-activated signaling pathway
regulation of stomatal closure
nucleosome organization
membrane fusion
RNA export from nucleus
mRNA polyadenylation
Golgi to vacuole transport
basipetal auxin transport
protein secretion
chloroplast accumulation movement
viral process
regulation of intracellular pH
fructose 6-phosphate metabolic process
positive regulation of cell growth
intra-Golgi vesicle-mediated transport
chloroplast avoidance movement
starch catabolic process
activation of GTPase activity
negative regulation of innate immune response
negative regulation of response to external stimulus
negative regulation of response to biotic stimulus
mRNA export from nucleus
protein stabilization
stomatal closure
positive regulation of transport
vegetative phase change
phospholipid translocation
response to mannitol
malate metabolic process
B
Cellular Component
T a b c
nuclear dicing body
cytosol
nucleoplasm
plasma membrane
cell plate
organelle outer membrane
nuclear body
P-body
U2-type spliceosomal complex
chromatin
cell
nuclear envelope
catalytic step 2 spliceosome
U4/U6 x U5 tri-snRNP complex
phragmoplast
proteasome regulatory particle
nuclear chromatin
histone deacetylase complex
small-subunit processome
late endosome
SWI/SNF superfamily-type complex
cell cortex
cell tip
SNARE complex
nuclear pore
cortical microtubule cytoskeleton
late endosome membrane
cortical microtubule
site of polarized growth
preprophase band
protein phosphatase type 2A complex
plant-type vacuole
histone methyltransferase complex
cell surface
exocyst
integral component of mitochondrial outer membrane
cytoplasmic microtubule
photosystem I
integral component of chloroplast outer membrane
nucleosome
vesicle membrane
perinuclear region of cytoplasm
Cajal body
COPI vesicle coat
transcription factor TFIID complex
clathrin-coated vesicle
myosin complex
early endosome
cellulose synthase complex
90S preribosome
chaperonin-containing T-complex
SAGA complex
Prp19 complex
post-mRNA release spliceosomal complex
U2-type catalytic step 1 spliceosome
cell division site
recycling endosome
SCAR complex
nuclear membrane
trans-Golgi network transport vesicle
clathrin adaptor complex
clathrin coat of coated pit
clathrin coat of trans-Golgi network vesicle
GARP complex
VCP-NPL4-UFD1 AAA ATPase complex
Tic complex
mRNA cleavage and polyadenylation specificity factor complex
UBC13-MMS2 complex
granal stacked thylakoid
Cdc73/Paf1 complex
trans-Golgi network transport vesicle membrane
extracellular exosome
clathrin vesicle coat
PSII associated light-harvesting complex II
chloroplast starch grain
transcription factor TFIIK complex
mRNA cap binding complex
polysome
exon-exon junction complex
HOPS complex
CORVET complex
nucleotide-activated protein kinase complex
Log2(Fold)
6
0

Figure S6

(A) Biological process and (B) cellular component enrichment analysis of Arabidopsis orthologs of total phosphoproteins (T) and phosphoproteins with 1 to 4 phosphosites (a), 5 to 10 phosphostes (b) and larger than 10 phosphosites (c) listed in **Supplementary Table S1e**. The blue color palette of cell represents the logarithm of the fold enrichment.

Figure S7

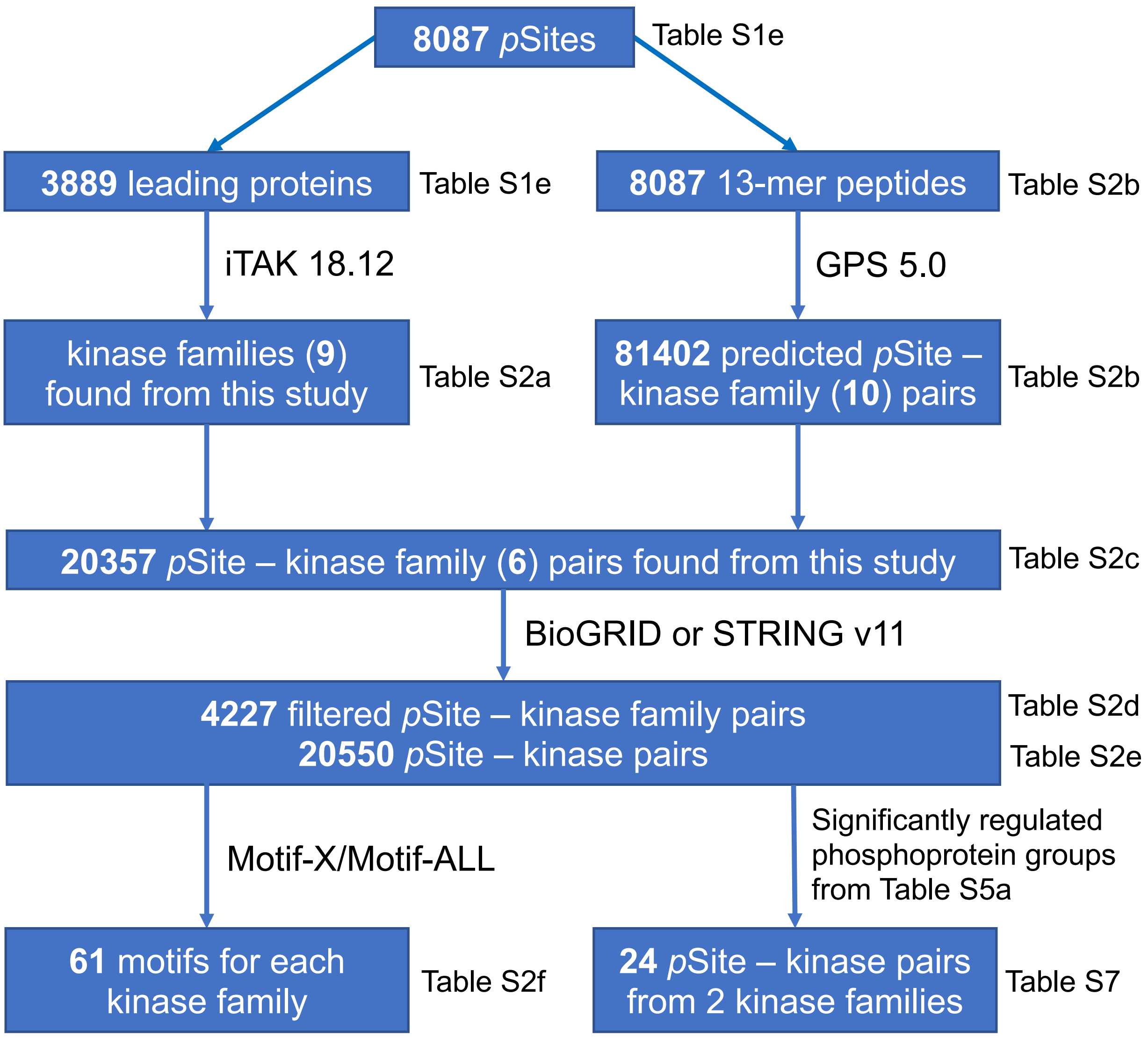

8087 pSites
Table S1e
3889 leading proteins
Table S1e
8087 13-mer peptides
Table S2b
iTAK 18.12
GPS 5.0
kinase families (9) found from this study
Table S2a
81402 predicted pSite – kinase family (10) pairs
Table S2b
20357 pSite – kinase family (6) pairs found from this study
Table S2c
BioGRID or STRING v11
4227 filtered pSite – kinase family pairs
20550 pSite – kinase pairs
Table S2d
Table S2e
Motif-X/Motif-ALL
Significantly regulated phosphoprotein groups from Table S5a
61 motifs for each kinase family
Table S2f
24 pSite – kinase pairs from 2 kinase families
Table S7

Figure S7. The mapping of the relationship in between kinase family and the kinase docking site motif.

From the 3889 leading proteins (**Supplementary Table S1g**), those annotated kinases were classified as 9 kinase families using software iTAK (http://itak.feilab.net/cgi-bin/itak/index.cgi, listed in **Supplementary Table S6a**). The 8087 amino acid sequences surrounding a phosphosite (**Supplementary Table S1e**) were extended to 6 aa long at both ends. The possible kinase families (10 in total) that may catalyze these phosphosites are predicated by GPS 5.0 (Group-based Phosphorylation Scoring 5.0, http://gps.biocuckoo.cn/, resulting in 81402 pairs listed in **Supplementary Table S2b**). Consequently, the kinase families grouped by iTAK and those kinase families - phosphosites pairing results predicted by **GPS 5.0** were integrated together to map the relationship of phosphosites-dependent kinase families (20357 pairs and 6 kinase families listed in **Supplementary Table S2c**). The total number of phosphosites-kinase family mapping results were further filtered with the protein-protein interaction data predicted either by STRING or BioGrid (Database of Protein, Genetic and Chemical Interactions, https://thebiogrid.org/, resulting in 4227 pairs listed in **Supplementary Table S2d**). These 4227 pairs can be further extended into 20550 phosphosites-kinase pairs (**Supplementary Table S2e**). The Motif-X (Chou and Schwartz, 2011) and Motif-All (He et al., 2011) were used to enrich the phosphosite motifs (61 in total) within each kinase families (**Supplementary Table S2f**). In addition, integrating of the substrates-kinase mapping (**Supplementary Table S2e)** with the significantly regulated phosphoprotein groups **(Supplementary table S5a)** results in 24 phosphosites-kinase mapping and 2 kinase families, which can be further combined into 20 phosphoprotein-kinase mapping (**Supplementary table S7**).

Figure. S8

A

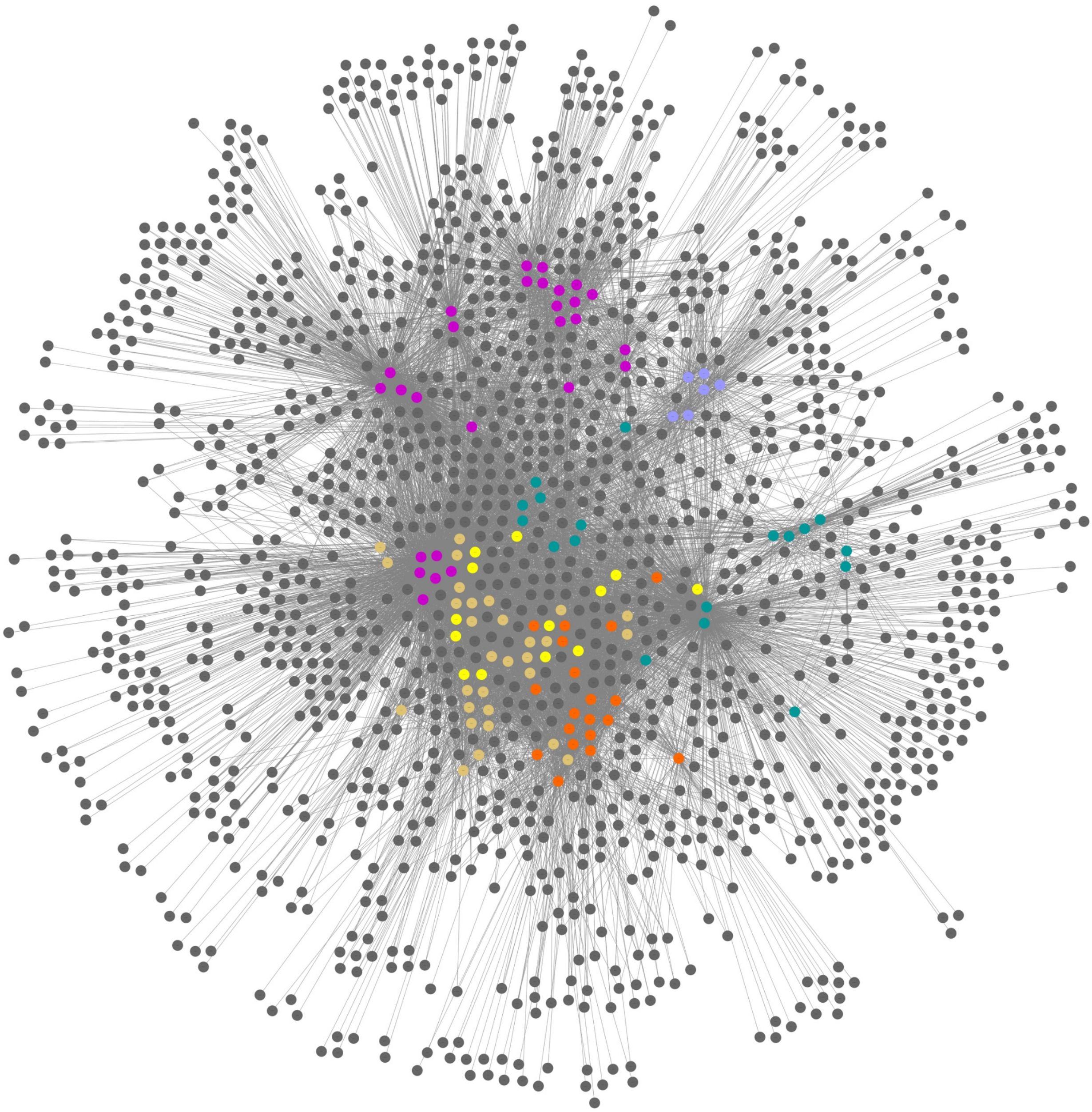

B

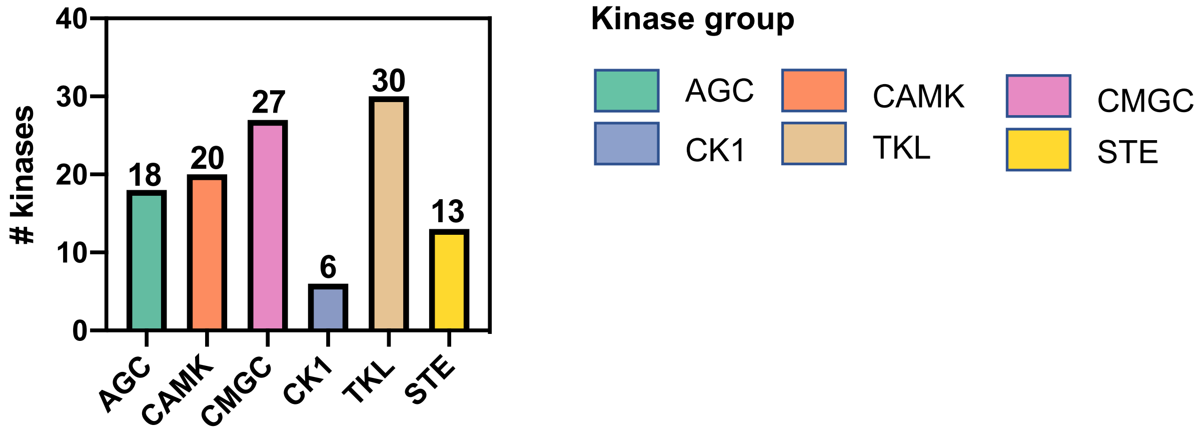

kinases
40
30
20
10
0
18
20
27
6
30
13
AGC
CAMK
CMGC
CK1
TKL
STE
Kinase group
AGC
CAMK
CMGC
CK1
TKL
STE

Figure S8. Interactome of kinase and substrates with GPS predication and STRING/BioGRID filtering.

(A) The edges (20550) represent the interactions between the substrates and kinases generated by GPS predication and STRING/BioGRID filtering (**Supplementary Table S2e**). The nodes (1403) represents both substrate and kinase proteins (**Supplementary Table S2e**). The green-, purple-, orange-, beige-, pink- and yellow-colored nodes represent AGC, CK1, CAMK, TKL, CMGC and STE kinase family, respectively.

(B) The bar chart of the number individual kinase proteins in the six kinase families **(Supplementary Table S2d, S2e).**

Figure. S9

GmPLDRP1
XP_003554616.1-S141

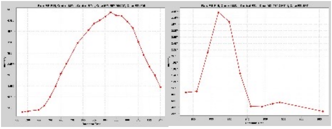

GmPLDRP1
XP_003554616.1-S290

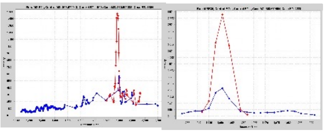

GmPLDRP1
XP_003554616.1-S178

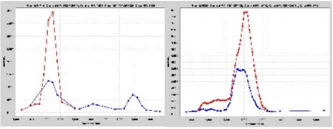

GmLTI65
XP_003556105.1-S513

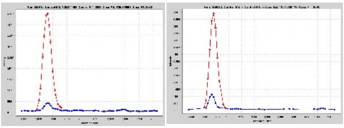

GmLTI65
XP_003556105.1-S123

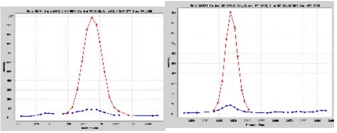

GmLTI65
XP_003556105.1-S344

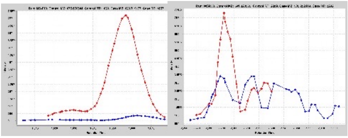

GmLTI65
XP_003556105.1-S423

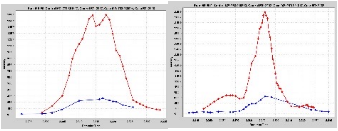

GmLTI65
XP_003556105.1-S400

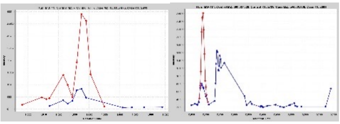

GmLTI65
XP_003556105.1-S183

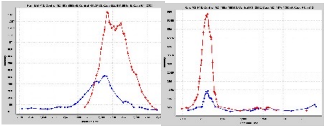

GmLTI65
XP_003556105.1-S494

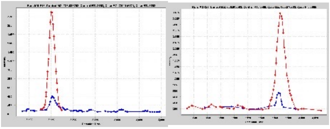

GmLTI65
XP_003556105.1-S333

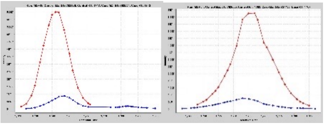

GmLTI65
XP_003556105.1-S231

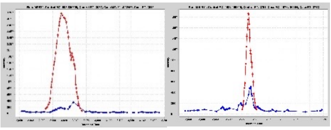

GmLTI65
XP_003556105.1-S442

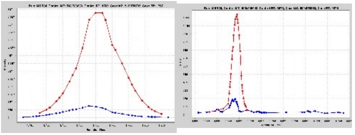

GmLTI65
XP_003556105.1-S446

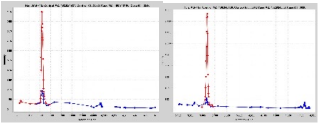

GmLTI65
XP_003556105.1-S529

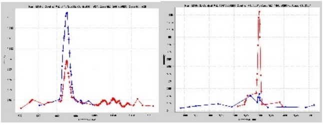

GmDI8
NP_001234972.1-S174

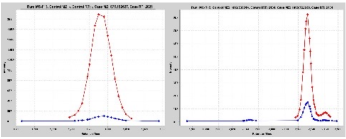

GmDI8
NP_001234972.1-S36

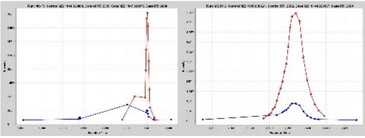

GmDI8
NP_001234972.1-S50

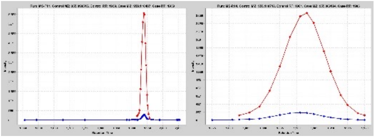

GmAMPD
XP_006576522.1-S50-S51

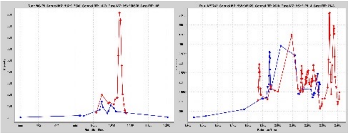

GmLHCB1.3
NP_001241277.1-T36

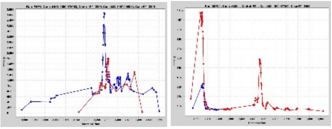

GmCLPP3
XP_003518640.1-T189;
XP_003552706.1-T189

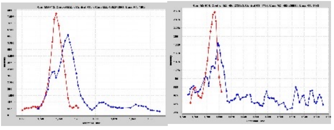

Figure. S9

GmBLUS1
XP_003536176.1-S476;XP_006589235.1-S476;XP_014618721.1-S474

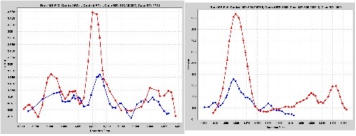

GmMAP2B
NP_001276226.1-S35;XP_003524568.1-S35;XP_006579792.1-S35;XP_006579793.1-S35;XP_006579795.1-S35;XP_014630823.1-S35

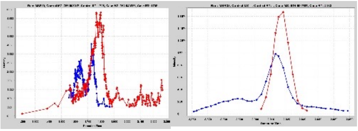

GmVAP2-2
XP_006589156.1-S160;XP_006589157.1-S160;XP_014618680.1-S160

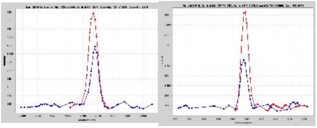

GmPLDRP1
XP_003543859.3-S204;XP_003543859.3-S230;XP_003543859.3-S269;XP_003543859.3-S295

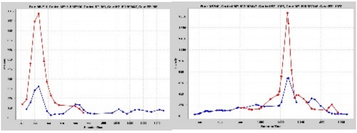

GmPLDRP1
XP_003543859.3-S217;XP_003543859.3-S256

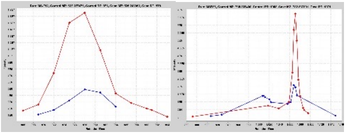

GmPLDRP1
XP_003543859.3-S136;XP_003543859.3-S162

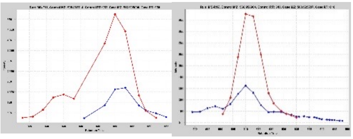

GmPLDRP1
XP_003543859.3-S308

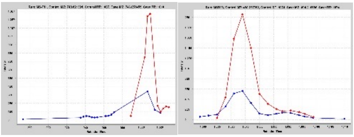

GmPLDRP1
XP_003543859.3-S149;XP_003543859.3-S175

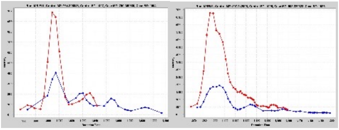

GmSR45a
XP_014620442.1-T156;XP_014623898.1-T154

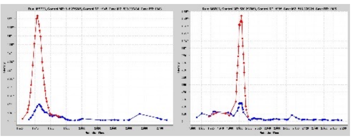

GmATHB-12
XP_003528775.1-S35;XP_003548207.2-S41

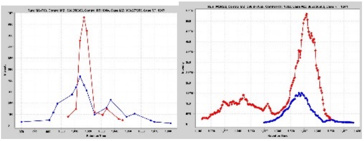

GmDTSP1
XP_003533837.1-S130;XP_014617508.1-S130

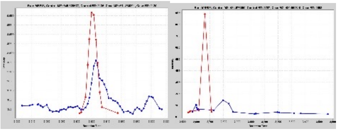

GmCNX1
NP_001242529.1-S440

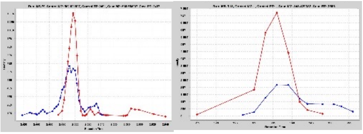

GmILP1
XP_006605552.1-S235

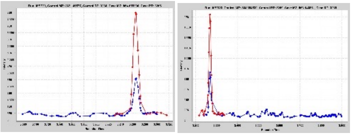

GmSMP28
NP_001237986.1-S31

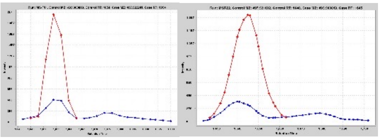

GmPUX5
XP_003545566.1-S208

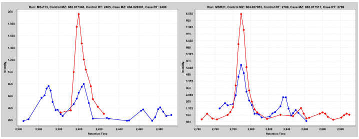

Figure. S9

GmCYS-3A
NP_001276178.1-S313;XP_006576133.1-S313

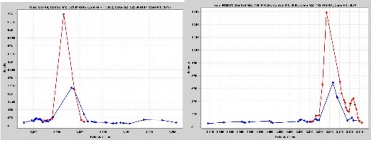

GmUGE5
XP_014620442.1-T156;XP_014623898.1-T154

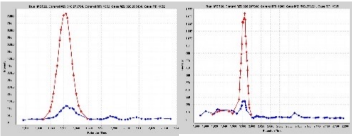

GmCSP2
XP_003522723.1-S182

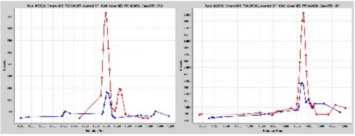

Gmzf-CCHC
XP_003521598.1-S45;XP_003554558.1-S45;XP_006577174.1-S45;XP_006604749.1-S45

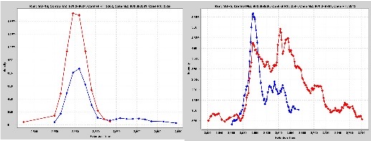

GmGPX6
NP_001236895.1-S128

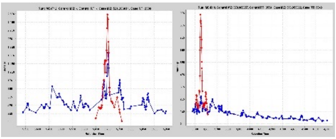

GmASR5
XP_003536424.1-S135

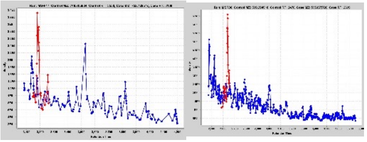

GmCHUP1
XP_006573276.1-S623

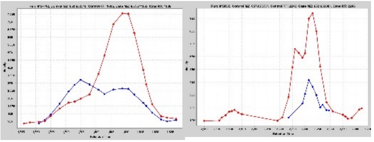

GmCSP4
XP_003537800.1-S189

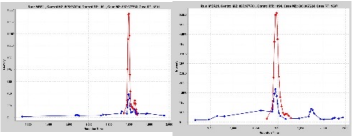

GmTCF25
XP_003547990.1-S21

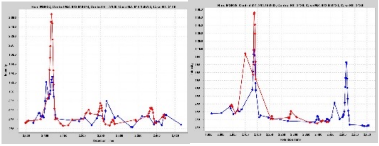

GmREM1.2
XP_003528866.1-S61

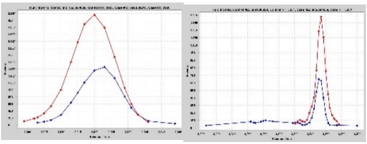

GmREM1.2
XP_003528866.1-S61-S64

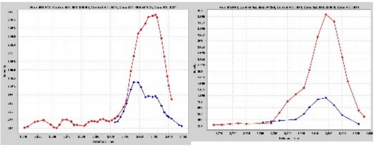

GmTCF25
XP_003547990.1-S21

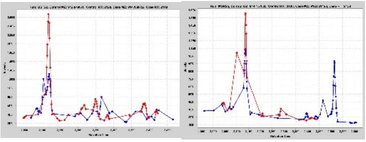

GmPPI1
XP_003543239.1-S198;XP_006594761.1-S198

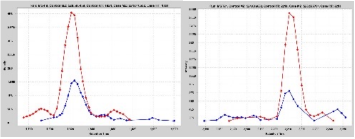

GmP5CS2
XP_003520914.1-S118;XP_006576558.1-S66z

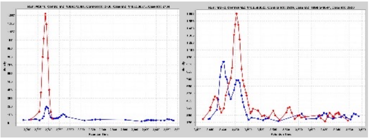

GmIQD1
XP_003521377.1-S447;XP_003537265.1-S445;XP_003541403.1-S441;XP_003554363.1-S447;XP_006577010.1-S447;XP_006577011.1-S447;XP_006594122.1-S441;XP_006604562.1-

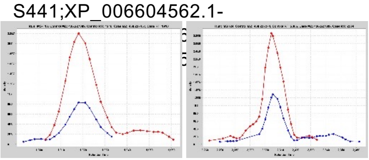

Figure. S9

GmCOR47
NP_001240106.1-
S52

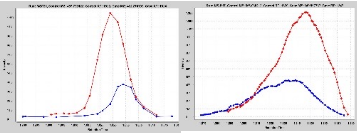

GmCOR47
NP_001240106.1-
S60

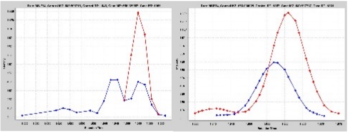

GmLTI65
XP_003535616.2-
S420;XP_014618839.1-
S420;XP_014618840.1-
S415

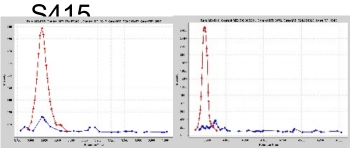

GmXIK
XP_006593940.1-
S1520;XP_006600
449.1-S1520

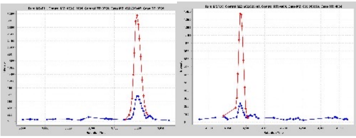

GmPIP2;2
NP_001235204.1-
S10;NP_001240039.1-
S10;XP_003518297.1-
S10

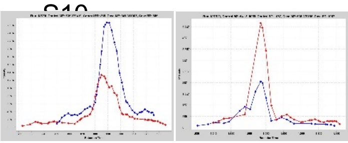

GmMETTL
XP_006583595.1-
S267;XP_006583596.1-
S267

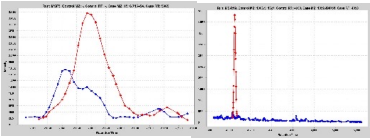

GmPLDRP1
NP_001237487.1-S63

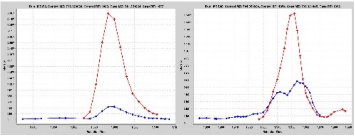

GmPLDRP1
NP_001237487.1-S67-S68

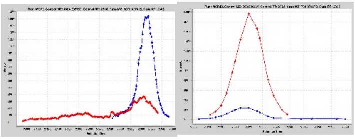

GmPLDRP1
NP_001237487.1-S68

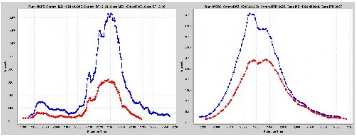

GmAHA11
XP_003554288.
1-
T889;XP_01462
0779.1-T925

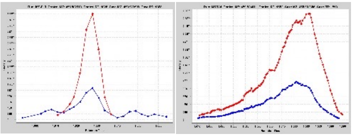

GmSRK2I XP_003517280.1-
S29;XP_003518861.1-
S26;XP_003524577.1-
S31;XP_003529248.1-
S31;XP_003531593.1-
S12;XP_003537362.1-
S29;XP_003540159.1-
S12;XP_003550077.3-
S31;XP_003555707.1-
S31;XP_006575017.1-
S20;XP_006583867.1-
S26;XP_006583868.1-
S26;XP_006585497.1-
S12;XP_014619344.1-
S29;XP_014631577.1-
S29;XP_014633579.1-
S31;XP_014633580.1-S31

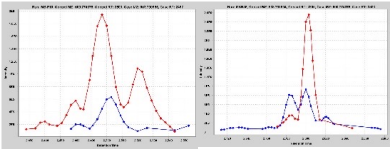

GmPIP2;2
NP_001235204.
1-S278-
S281;XP_00354
8071.1-S278-
S281

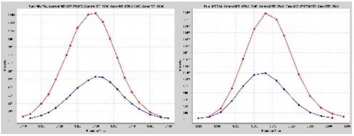

Figure. S9

GmVLN3
XP_003535925.1-S923;XP_006589012.1-S923;XP_006589013.1-S923;XP_006589014.1-S919

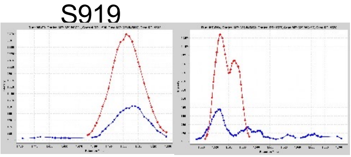

GmGCT
XP_006601868.1-S309;XP_006601869.1-S308;XP_014626292.1-S308

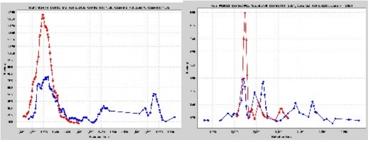

GmSCAMP5
NP_001241214.1-S38

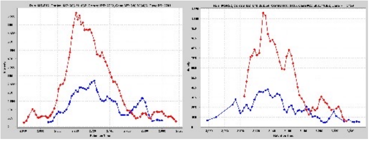

GmFREE1
XP_003527583.1-S480

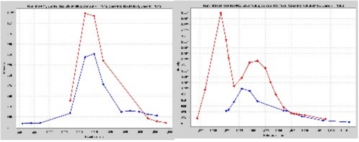

GmAMT
XP_003529643.1-S267;XP_003546157.1-S267;XP_006600302.1-S267

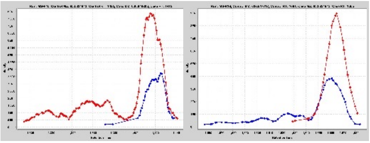

GmNAP1;2
NP_001238314.1-S39;XP_003533760.1-S41;XP_003533761.1-S41;XP_006597072.[illegible]

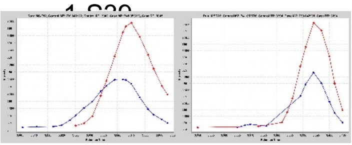

GmDTSP3
XP_006606559.1-S500;XP_006606560.1-S500

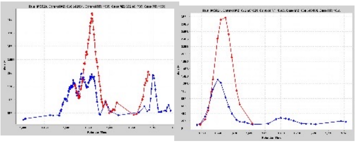

GmEIFISO4G1
XP_003522928.1-S552;XP_003527199.1-S551;XP_003549535.1-S562;XP_006575314.1-S559;XP_014630176.1-S510

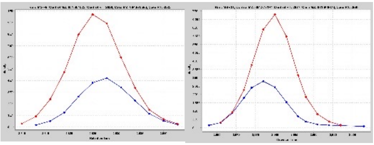

GmLHCB1.3
NP_001235283.1-T36-T38

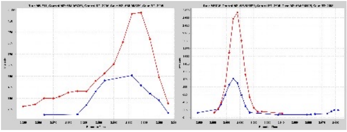

GmCYS6
NP_001237443.1-S141

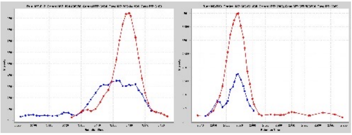

GmG3BP
XP_003521311.1-S270;XP_006576961.1-S269

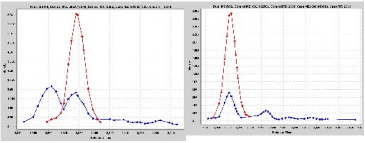

GmNAP1;2
NP_001241118.1-S41;XP_003549222.1-S41

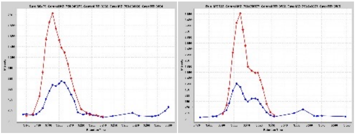

Figure. S9

GmABCG22
XP_003519092.1-S91;XP_003535833.2-S126;XP_006575266.1-S130;XP_006588894.1-S126

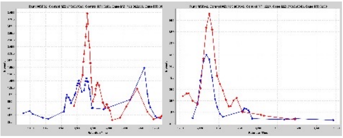

GmIQD2
XP_003521377.1-S378;XP_006577010.1-S378;XP_006577011.1-S378

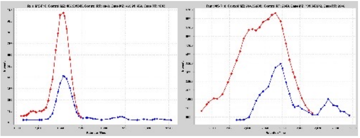

GmTPL
XP_006585625.1-S214;XP_006585626.1-S214;XP_014633159.1-S214;XP_014634634.1-S214;XP_014634635.1-S177

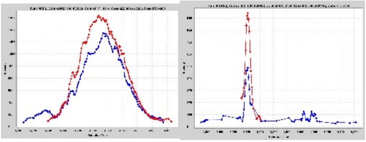

GmDEP1
XP_003540469.1-S263;XP_003543236.1-S263

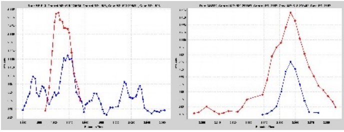

GmDTSP4
NP_001239719.1-S152;XP_003553921.1-S151

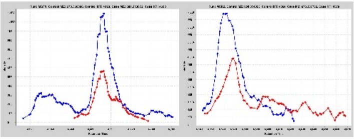

GmPEX5
NP_001237339.1-S133;XP_014631619.1-S133;XP_014631620.1-S133

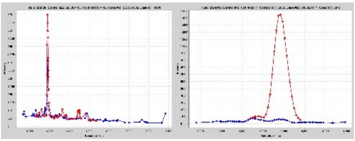

GmTCP11
XP_006585494.1-S871;XP_006592672.1-S871

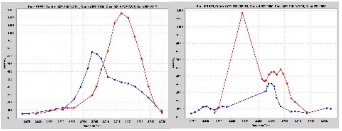

GmSYP51
XP_003532151.1-S125;XP_003551745.1-S126;XP_006586106.1-S125;XP_006602182.1-S148

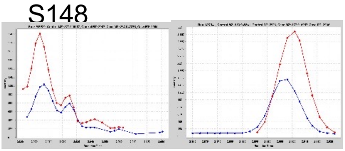

GmSERBP1
XP_003529036.1-S115

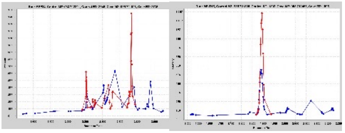

GmBSL1
XP_003518942.1-S839;XP_003536338.1-S839

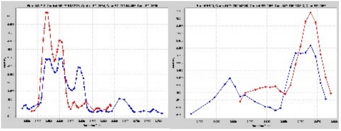

GmPUB4
XP_003551173.1-S490

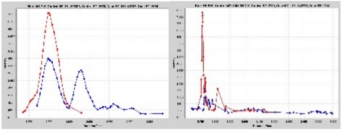

GmMET
NP_001240011.1-S150;XP_006577864.1-S150;XP_006581541.1-S150;XP_014631808.1-S150

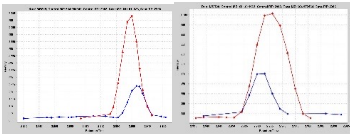

GmGST8
NP_001238675.1-S224

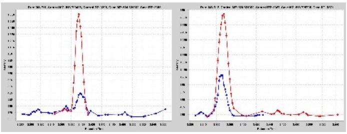

GmABCG22
XP_003535833.2-S91;XP_006588894.1-S91

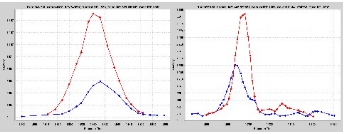

GmEIN2
XP_003542536.1-S643;XP_006588798.1-S660;XP_006588799.1-S644;XP_014618493.1-S644

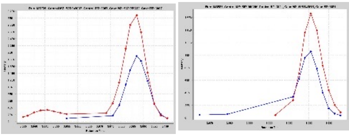

Figure. S9

GmPHDP
XP_006590589.2-
S2538

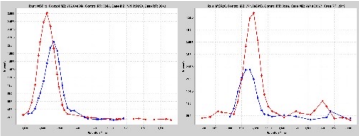

GmPDP5
XP_006589021.1-
S142

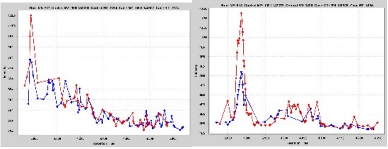

GmIQD14
XP_003518875.1-
S476;XP_003529373.1-
S475;XP_006575031.1-
S477;XP_006575032.1-
S477;XP_006583852.1-
S475;XP_006583853.1-
S474;XP_014622544.1-
S477;XP_014622549.1-
S476;XP_014633627.1-S474

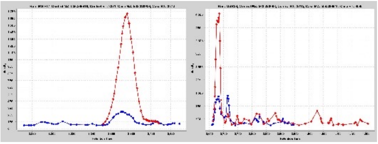

GmIQD13
XP_003518875.1-
S485;XP_003529373.1-
S484;XP_006575031.1-
S486;XP_006575032.1-
S486;XP_006583852.1-
S484;XP_006583853.1-
S483;XP_014622544.1-
S486;XP_014622549.1-
S485;XP_014633627.1-
S483

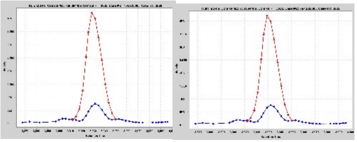

GmPIP2-6
NP_001237286.1-
T9;XP_003540176.1-T9

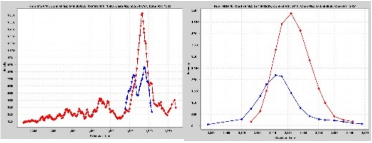

GmHLN
XP_003521066.1-
S115

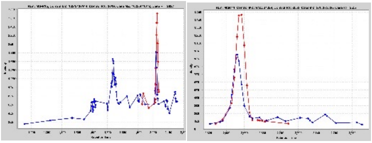

GmGMP
NP_001239631.1-S314

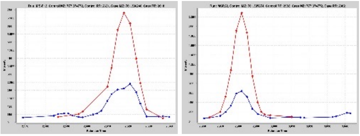

GmRSZ32
NP_001242616.1-
S240;XP_006576250.1-
S240;XP_006576251.1-
S240;XP_006576252.1-
S240;XP_006576253.1-S199

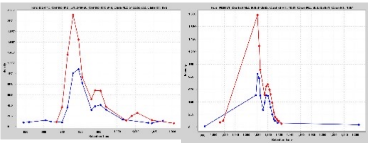

GmNPL41
XP_003521523.1-
S103;XP_00355449
1.1-S103

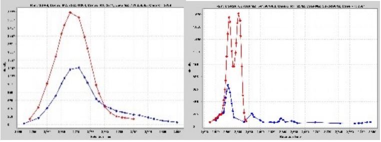

Figure. S9

GmDTSP5
NP_001239816.1-S102

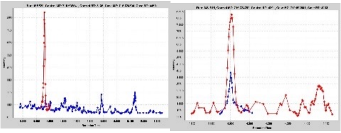

GmGME
NP_001242764.1-S368;NP_001276258.1-S368;XP_003521749.1-S368;XP_003554693.1-S368;XP_003554694.1-S368;XP_003556467.1-S368;XP_006588433.1-S368;XP_006606460.1-S384

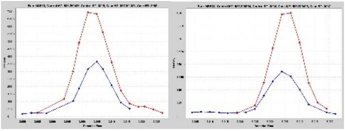

GmLOX1
NP_001235189.1-S37

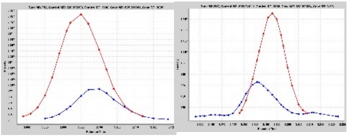

GmKHDRB
XP_003522728.1-S25

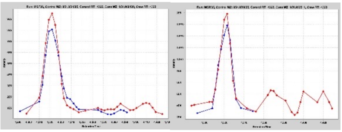

GmFAM188A
XP_003536430.1-S61;XP_003556149.1-S61;XP_006606163.1-S61

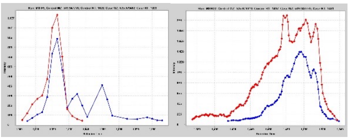

GmF2KP
XP_003552945.1-S256;XP_006591237.1-S256;XP_006602083.1-S256

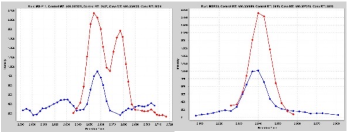

GmC3H17
XP_006580078.1-S326;XP_006585058.1-S323

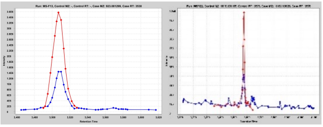

GmMOT2
XP_003531693.1-S453

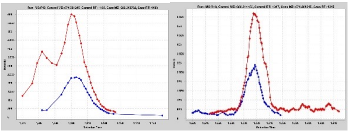

GmLHCB4.2
XP_003516950.1-T111-T113;XP_003520609.2-T111-T113

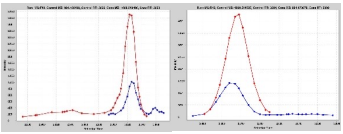

GmHB-1
XP_006583834.2-S121;XP_006583835.2-S121;XP_006583836.2-S121;XP_006594306.1-S122;XP_006594307.1-S122;XP_014621115.1-S122;XP_014621116.1-S122

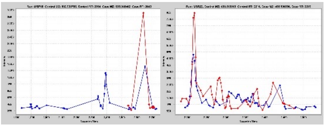

GmRH20
XP_003529884.1-S54;XP_003531619.1-S54;XP_006583016.1-S54

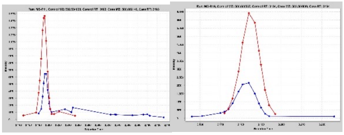

GmAML5
XP_003546575.1-S639

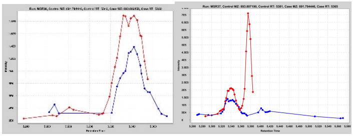

Figure. S9

GmATRX
XP_003531752.1-S468;XP_003538884.1-S297;XP_006585654.1-S469

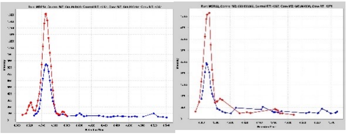

GmPERK1
XP_003529582.1-S489

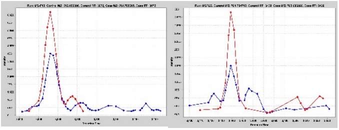

GmAKINβ1
XP_003519491.1-S57;XP_003545472.1-S

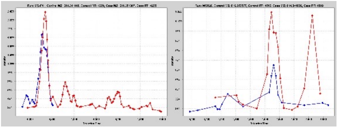

GmNCL
XP_003540477.1-S320

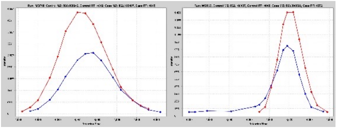

GmPRA1.F2
NP_001235529.1-S177

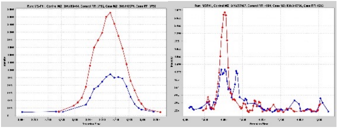

GmPAT1
XP_003532940.1-S86;XP_003545913.2-S85;XP_006585424.1-S86

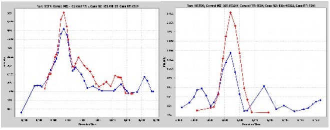

GmKLCR1
XP_003530449.1-S57

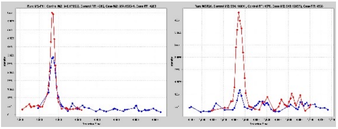

GmDTSP5
NP_001239664.1-S22

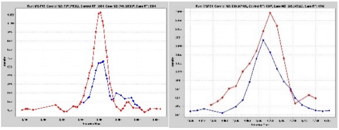

GmARD2
NP_001236651.1-S192

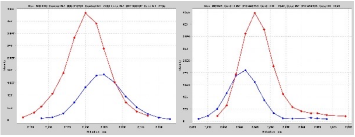

GmSPEN2
XP_003556809.1-S291;XP_014628135.1-S177

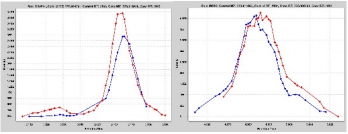

GmTUA5
NP_001237301.1-T349;NP_001242878.1-T349;XP_003554022.1-T349;XP_006579487.1-T349;XP_014624621.1-T349

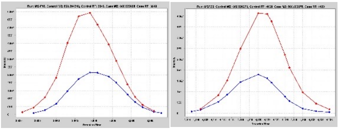

GmCIMS
NP_001235794.1-S602;XP_003522969.1-S640;XP_003525709.1-S602;XP_00355108
S602;XP_003554033.1-S602;XP_0065798
S602;XP_006601025.1-S602;XP_0146248
S602;XP_014627290.1-S602

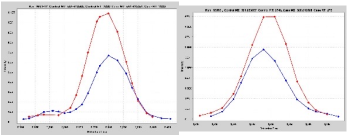

GmREM
NP_001236279.1-S60

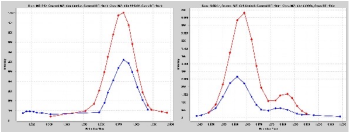

GmLHCB2
NP_001240183.1-S43;NP_001241179.1-S43

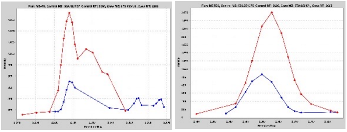

GmTUA2
XP_003517369.1-T349;XP_003522731.1-T349;XP_003524707.1-T349;XP_003536570.1-T349;XP_003539228.1-T349;XP_003556001.1-T349;XP_006581474.1-T349;XP_014625350.1-T349;XP_014631758.1-T349

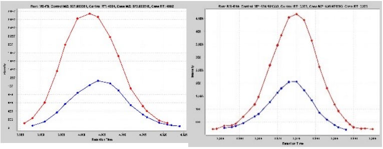

GmMKKK
XP_006592871.1-S415;XP_006594821.1-S415;XP_006594822.1-S385;XP_014620555.1-S415;XP_014620556.1-S385;XP_014621435.1-S383

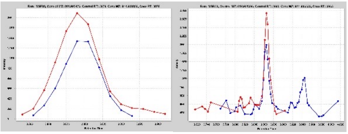

GmBTR1
XP_003536289.1-S21;XP_006589354.1-S21

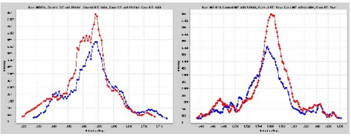

GmPSBR
NP_001235442.1-T96;NP_001236219.1-T95

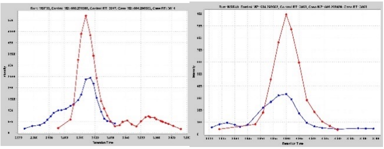

Figure. S9

GmPIP2;1
XP_003532817.1-S16

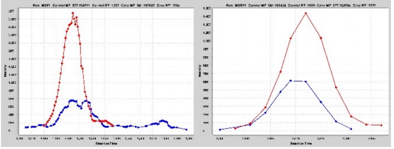

GmPIP2;1
XP_003554376.1-S11

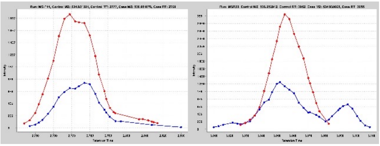

GmPIP2;2
NP_001240223.1-
S15;XP_003538174.1-S15

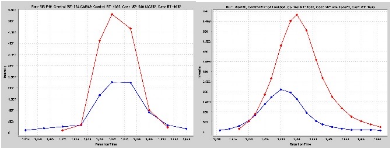

GmSEC15B
XP_006577151.1-
S675;XP_006595636.1-S675

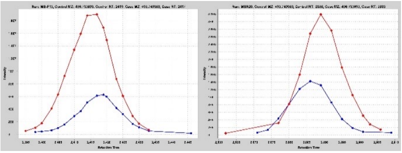

GmSTN7
XP_003516854.1-S541

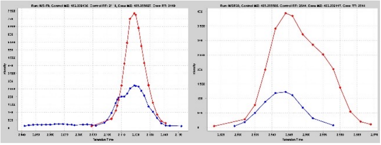

GmMKK4
XP_003529493.1-
T34;XP_003531771.1-T38

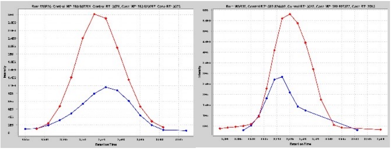

GmPDS5A
XP_006586783.1-S1644

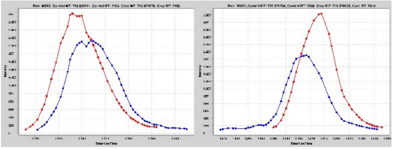

GmPSBS
NP_001276237.1-T173

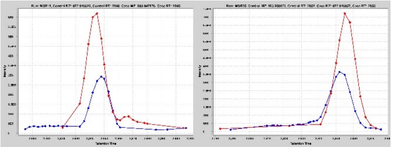

GmEFTs
XP_003522714.1-S9-
S13;XP_003526494.2-S32-
S36;XP_014623824.1-S29-S33

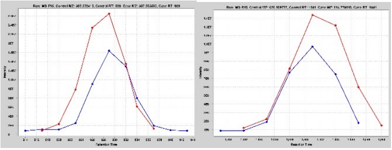

GmGSL07
XP_006586073.1-T27

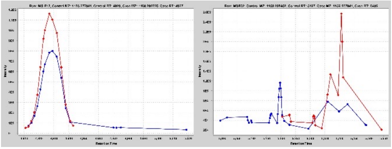

GmPIP2-7
XP_003522619.3-S268-
S271;XP_006581099.1-S673-S676

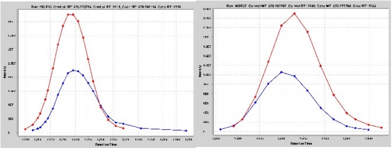

GmWD40R
XP_003524447.1-S1395

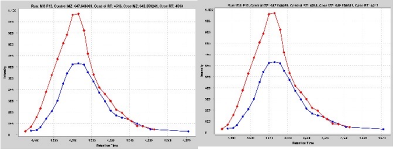

GmPSBQ
NP_001236143.1-S127

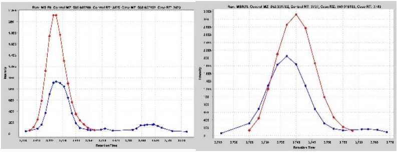

GmUPL1
XP_003527888.2-
S2286;XP_006575360.1-
S2282;XP_006575361.1-
S2282;XP_006596386.1-
S2285;XP_006596387.1-
S2285;XP_014630060.1-
S2283;XP_014630061.1-
S2283;XP_014630062.1-S2283

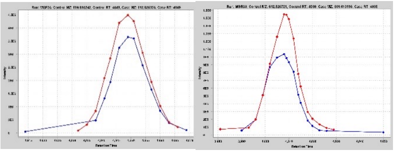

GmLHCB6
NP_001236415.1-T210

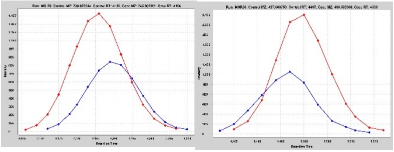

Figure. S9

GmTCP25
XP_006583190.1-S21

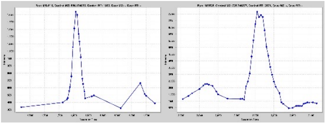

GmLIG1
XP_003550133.1-S239

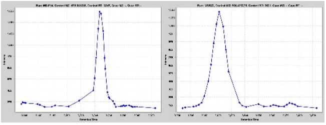

GmNST1
XP_003526458.1-S32

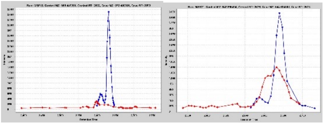

GmCMT2
XP_006599215.1-S609;XP_014624380.1-S609;XP_014624381.1-S533

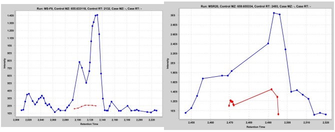

GmPK
XP_003545710.2-S824;XP_006597992.1-S824

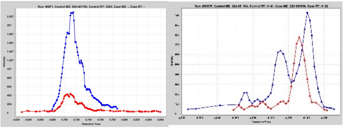

GmDEF1
XP_014634399.1-S308;XP_014634400.1-S308

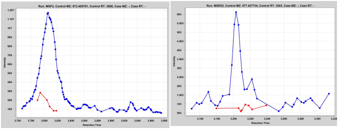

GmMYH2
XP_006590781.1-S600;XP_014619416.1-S600

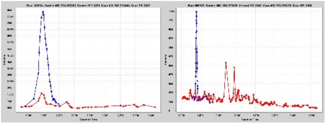

GmDDX31
XP_003548422.1-S249;XP_014624517.1-S232

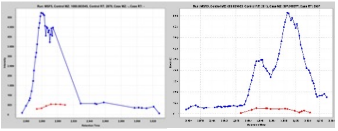

GmBFRUCT1
NP_001236325.1-S150;XP_003531611.1-S150;XP_003544539.1-S152;XP_003549347.2-S136

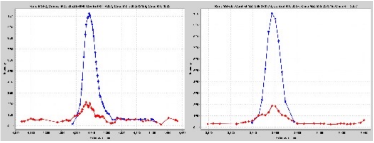

GmDRP
XP_003544933.2-T1244;XP_006596398.1-T1235;XP_006596472.1-T1244;XP_006596475.1-T1244

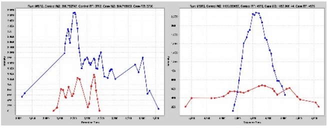

GmUBA2B
XP_006575611.1-S67;XP_014625731.1-S67;XP_014625732.1-S67

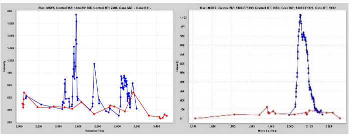

GmDRP
NP_001236212.1-S218;NP_001236910.1-S102;NP_001237902.1-S102;NP_001238254.1-S102;NP_001240935.1-S218;XP_006598718.1-S218

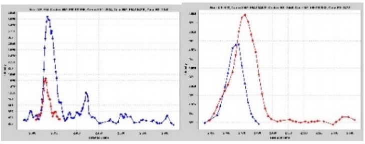

Figure. S9

GmTRP3
XP_014618667.1-T288;XP_014618668.1-T288

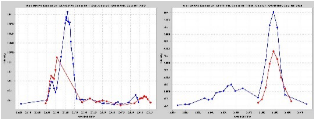

GmTITIN
XP_006589099.1-T174;XP_006589100.1-T174;XP_014618648.1-T198;XP_014618649.1-T197

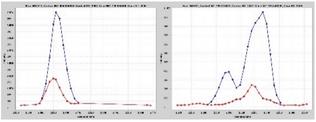

GmSYF1
XP_006594623.1-S858

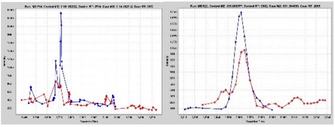

GmPSI1
XP_006578757.1-S606;XP_006581801.1-S603

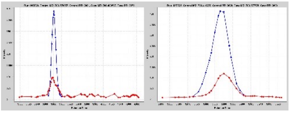

GmYSL7
XP_003533289.1-S383

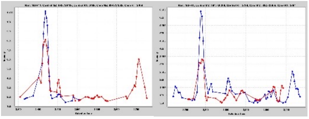

GmASIL2
XP_003523957.1-S67;XP_003539298.1-S55

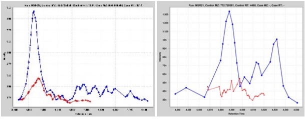

GmSHOU4
XP_003546110.1-S169

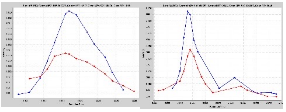

GmRING1
XP_003536735.1-S242

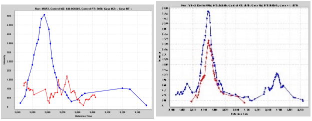

GmSPT6
XP_003521098.1-S6;XP_006604309.1-S6;XP_006604310.1-S6

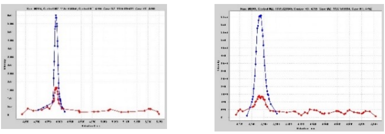

GmAAAP
XP_003523142.1-S650;XP_003526964.1-S654;XP_006579597.1-S640;XP_006579598.1-S612;XP_006581869.1-S659;XP_006581870.1-S659;XP_006581871.1-S656;XP_006581872.1-S626;XP_006581874.1-S592;XP_006600805.1-S640;XP_014630303.1-S655;XP_014630304.1-S655;XP_014630305.1-S622;XP_014630306.1-S590

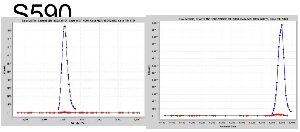

GmPPDK
XP_003550521.1-T530;XP_006600329.1-T530;XP_014633727.1-T533

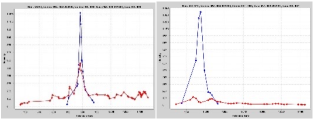

GmYSL7
XP_003548294.1-S382

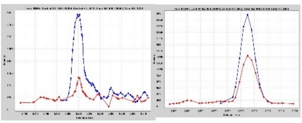

GmCOP1IP
XP_014618747.1-S1232

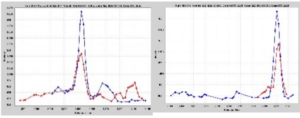

GmLYK4
XP_003518770.1-S321

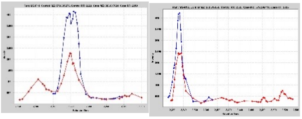

Figure. S9

GmHMT
XP_003528232.1-S1215

GmIDM
XP_006573883.2-S341

GmPK
NP_001235011.1-S325

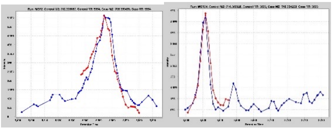

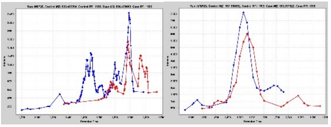

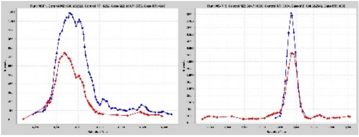

GmAPK18
XP_003523529.1-
T75;XP_003527639.1-
T51;XP_006577933.1-T75

GmPP2C
NP_001242598.1-
S131;XP_01462555
8.1-
S148;XP_01462555
9.1-
S148;XP_01462556

GmHOS1
XP_003538986.3-S933

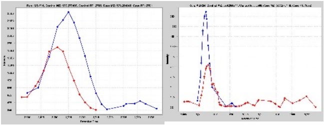

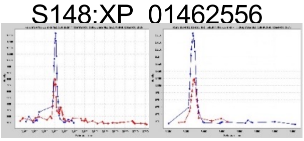

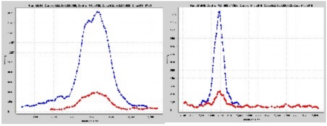

GmRH32
XP_003519572.1-
S533;XP_003544936.1-
S531;XP_006575677.1-S533

GmFRI1
XP_003547330.1-S285

GmDTSP6
XP_003547047.2-S522

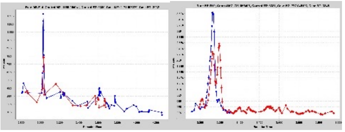

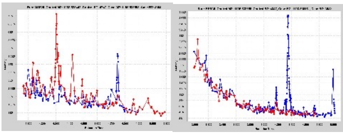

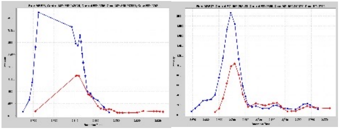

GmZF3C13
XP_003540851.1-
S246;XP_006592382.1-
S248;XP_006592383.1-
S248;XP_006592384.1-
S248;XP_006592385.1-
S246;XP_006592386.1-
S218;XP_006592388.1-
S187;XP_006592389.1-
S187;XP_006592390.1-
S187;XP_006592392.1-S187

GmTSJT1
XP_003543254.1-
S18;XP_014620570.1-S18

GmGBPL3
XP_003529353.1-
S976;XP_003542717.1
-S976

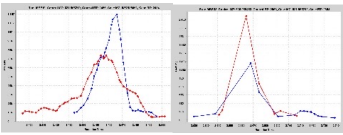

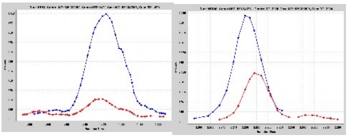

GmCHX20
XP_003520628.2-S443

GmSPT6
XP_003521098.1-
S6;XP_006604309.1-
S6;XP_006604310.1-S6

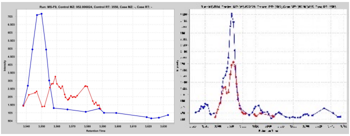

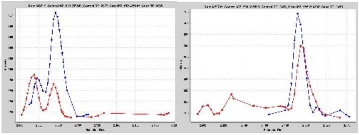

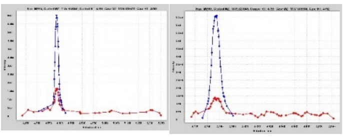

Figure. S9

GmEIF4E1
NP_001237528.1-S22

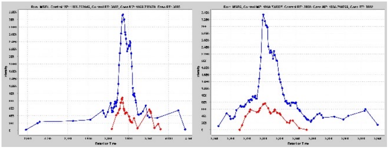

GmMDN
XP_014624660.1-S248

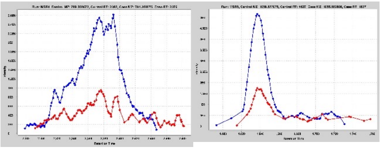

GmRPLP1
XP_003523320.1-S100-M104;XP_003526758.1-S100-M104

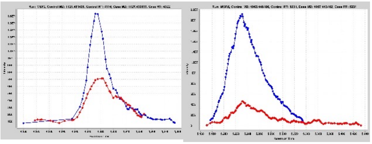

GmRPLP1
XP_003523320.1-S100;XP_003526758.1-S100

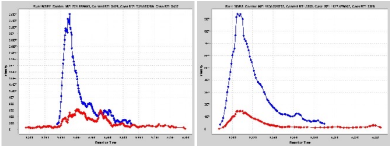

GmRPM1
NP_001235671.1-S141;XP_003534302.1-S143

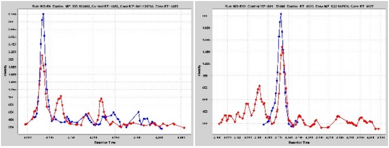

GmI-2
NP_001235846.1-S43;XP_006586619.1-S43;XP_006598709.1-S43;XP_006598710.1-S43;XP_014623900.1-S43;XP_014623901.1-S43;XP_014635028.1-S43

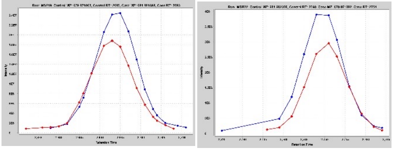

GmF12A12.60
XP_003537994.1-S186

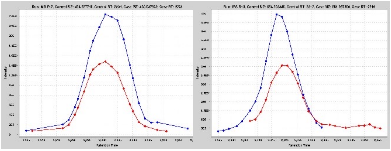

GmRBB1
XP_006599095.2-S1620;XP_006599096.2-S1620;XP_006599097.2-S1620;XP_006599099.2-S1620;XP_006599100.2-S858;XP_014624128.1-S1374

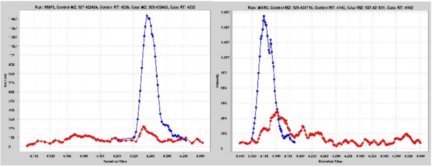

GmDSPP
XP_006579510.1-S858;XP_006579511.1-S858;XP_014630907.1-S858

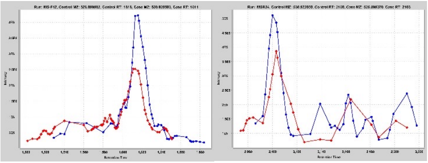

GmAATL1
XP_003537670.1-T89-S93

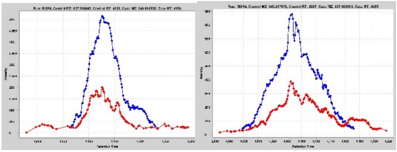

GmAATL1
XP_003537670.1-S93

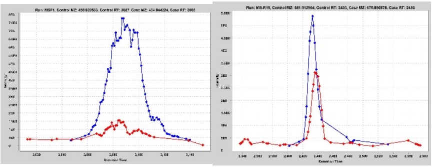

GmLRR-RKs
XP_014620583.1-S1001;XP_014620584.1-S977;XP_014620585.1-S1000;XP_014620586.1-S976

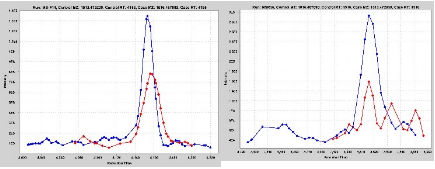

GmIQD28
XP_003517776.1-S106;XP_006573098.1-S106;XP_006573099.1-S81;XP_014628832.1-S106

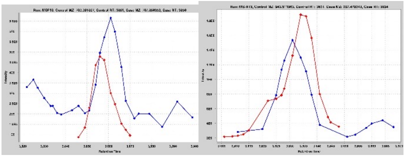

GmCBP
XP_006604331.1-S645

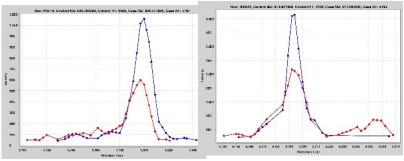

GmWIP1
XP_003526386.2-S227;XP_014630109.1-S225

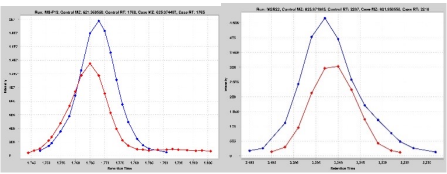

Figure. S9

GmKinX
XP_003531401.1-S65

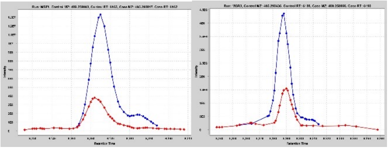

GmCRT1B
NP_001236351.1-S399;XP_003555807.1-S401

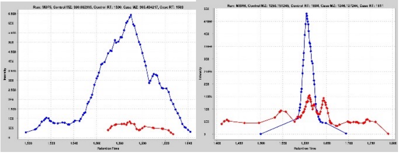

GmABCC3
XP_006575707.1-S846

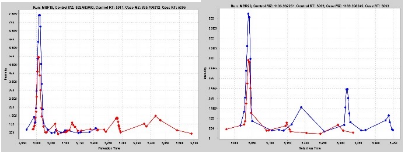

GmRPL10
NP_001238374.1-S310;NP_001239676.1-S309

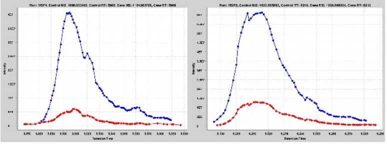

GmRPL10
NP_001238374.1-S310-M314;NP_001239676.1-S309-M313

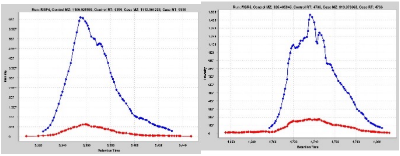

GmEF1B
NP_001237305.1-T116;XP_003526963.1-T116

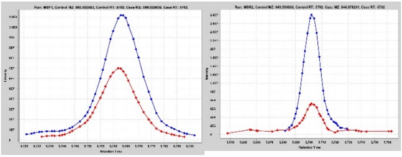

GmAPK1B
NP_001240863.1-S68;NP_001241128.1-S67;NP_001241432.1-S67;NP_001242670.1-S68;XP_006582915.1-S67;XP_006593429.1-S68;XP_006601852.1-S67;XP_006601853.1-S67

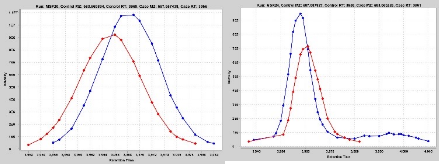

GmRAD21.3
XP_003533578.1-S170;XP_003551693.1-S170;XP_014626325.1-S170

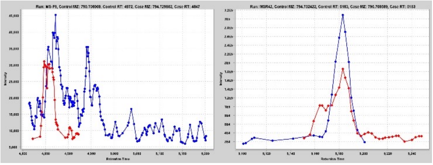

GmRPLP
NP_001236220.1-S101-M105;XP_003543439.2-S139-M143

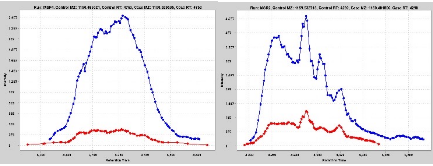

GmSCL30
XP_003518382.1-S5-S10;XP_003545260.1-S5-S10;XP_006575502.1-S5-S10

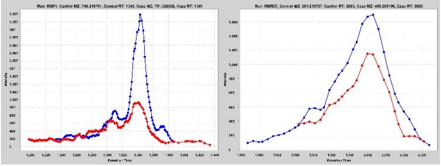

GmPBP
XP_006583789.1-S348;XP_006583790.1-S348

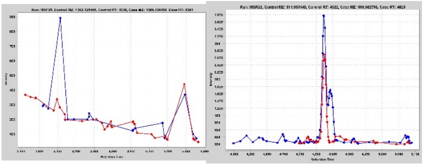

GmDTX40
XP_003540303.1-S34

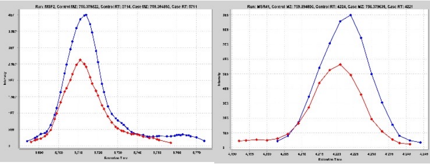

GmTSS
XP_003522940.1-S1621;XP_014630182.1-S1621

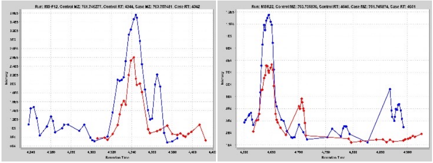

GmMYOB2
XP_006585081.1-S348

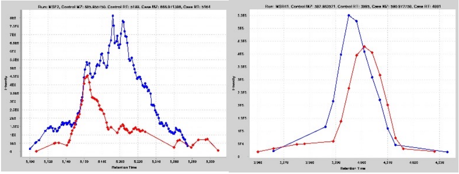

Figure. S9. The extracted ion chromatograms (XIC) of the significantly regulated phosphopeptides.

Left and right panel is an example of XICs identified from the forward mixing data set and reciprocal data set, respectively.

Red line stands for the drought-tolerant cultivar-specific while blue line stands for drought-sensitive cultivar specific. Each pair of XIC panels is marked by soybean gene name and UPSP (unique PTM site pattern) of the phosphopeptide (**Supplementary Table S4b**).

Figure. S10

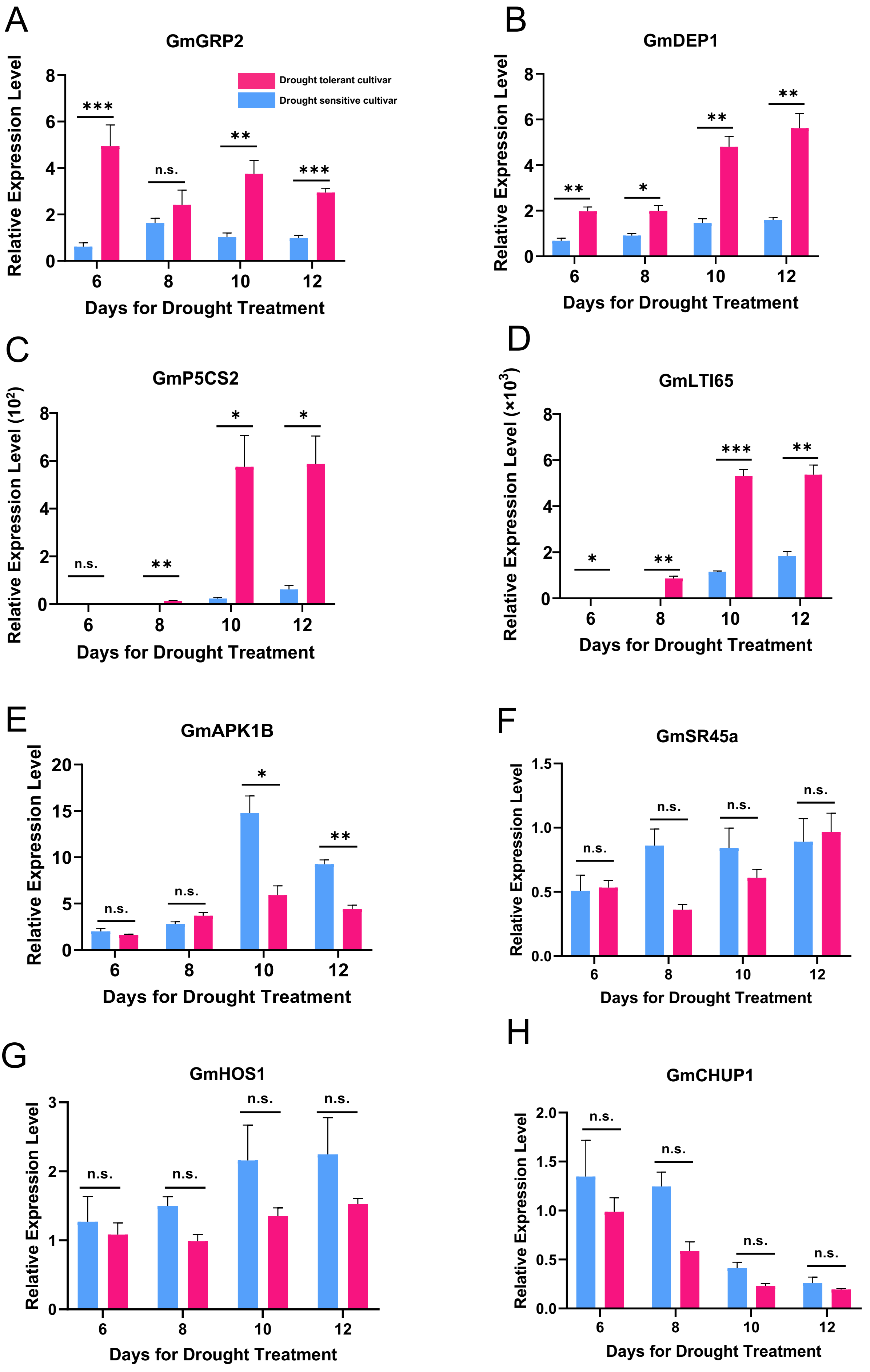
A
GmGRP2
Drought tolerant cultivar
Drought sensitive cultivar
Relative Expression Level
***
n.s.
**
***
Days for Drought Treatment
B
GmDEP1
Relative Expression Level
**
*
**
**
Days for Drought Treatment
C
GmP5CS2
Relative Expression Level (10^2)
n.s.
**
*
*
Days for Drought Treatment
D
GmLTI65
Relative Expression Level (×10^3)
*
**
***
**
Days for Drought Treatment
E
GmAPK1B
Relative Expression Level
n.s.
n.s.
*
**
Days for Drought Treatment
F
GmSR45a
Relative Expression Level
n.s.
n.s.
n.s.
n.s.
Days for Drought Treatment
G
GmHOS1
Relative Expression Level
n.s.
n.s.
n.s.
n.s.
Days for Drought Treatment
H
GmCHUP1
Relative Expression Level
n.s.
n.s.
n.s.
n.s.
Days for Drought Treatment

Figure S10. The RT-qPCR validation of representatives of the drought-tolerant cultivar significantly regulated phosphoprotein groups.

The gene expression of GmGRP2 (A), GmDEP1 (B), GmP5CS2 (C), GmLTI65 (D), GmAPK1B (E), GmSR45a (F), GmHOS1 (G), GmCHUP1 (H), is induced by 0, 6, 8, 10 and 12 days of drought treatment. The mRNA levels are quantified using RT-qPCR (see details in Material and Methods). Means ± SE of three biological replicates are indicated by error bars. Statistical analysis is performed by Student's pairwise *t*-test: *P < 0.05, **P < 0.01, ***P < 0.001, and n.s. (non-significant) P ≥ 0.05. The primers used in the RT-qPCR are listed in the **Supplementary Table S6.**
